# A new tropical Oligocene dolphin from Montañita/Olón, Santa Elena, Ecuador

**DOI:** 10.1371/journal.pone.0188380

**Published:** 2017-12-20

**Authors:** Yoshihiro Tanaka, Juan Abella, Gabriel Aguirre-Fernández, Maria Gregori, R. Ewan Fordyce

**Affiliations:** 1 Osaka Museum of Natural History, Osaka, Japan; 2 Numata Fossil Museum, Hokkaido, Japan; 3 Division of Academic Resources and Specimens, Hokkaido University Museum, Sapporo, Hokkaido, Japan; 4 Facultad de Ciencias del Mar, Universidad Estatal Península de Santa Elena, Santa Elena, Ecuador; 5 Institut Català de Paleontologia Miquel Crusafont, Universitat Autònoma de Barcelona, Edifici ICP, Campus de la UAB, Cerdanyola del Vallès, Barcelona, Spain; 6 Paleontological Institute and Museum, University of Zurich, Zurich, Switzerland; 7 Department of Geology, University of Otago, Dunedin, New Zealand; 8 Departments of Paleobiology and Vertebrate Zoology, National Museum of Natural History, Smithsonian Institution, Washington, DC, United States of America; University of California, UNITED STATES

## Abstract

A new small probable Oligocene dolphin from Ecuador represents a new genus and species, *Urkudelphis chawpipacha*. The new taxon is known from a single juvenile skull and earbones; it differs from other archaic dolphins in features including widely exposed frontals at the vertex, a dorsally wide open vomer at the mesorostral groove, and a strongly projected and pointed lateral tuberosity of the periotic. Phylogenetic analysis places it toward the base of the largely-extinct clade Platanistoidea. The fossil is one of a few records of tropical fossil dolphins.

## Introduction

Extant cetacean groups (Neoceti) originated from basilosaurid ancestors [1] during the Late Eocene [2], radiating to produce some crown family lineages by the end of the Oligocene [3–5]. This radiation involved an early rapid increase in morphological and ecological disparity, although the globally-sparse Early Oligocene record, with its widely mentioned preservational bias [2, 4–6], provides little direct support. Evolutionary patterns have yet to be quantified for Neoceti as a whole; meanwhile, the early increase in diversity and disparity has been shown in a total evidence phylogeny for one of the two main clades, Mysticeti [7].

Consider the advances in understanding the Neoceti radiation since Whitmore and Sanders' [8] historic summary of Oligocene Cetacea in the 1970s; developments have involved both theory (e.g. phylogenetics) and practice (e.g. new methods for preparation and analysis). To focus on odontocetes, historically-long established taxa have been redescribed using modern approaches (e.g. *Xenorophus*, *Agorophius*, *Archaeodelphis*) [9, 10]. Many new taxa have been named, adding to taxonomic and morphological diversity (e.g. *Albertocetus*, *Simocetus*, *Waipatia*, *Otekaikea*) [9, 11–15]. Ecologically-important behavior, such as feeding and echolocation, has been inferred from structure using extant phylogenetic bracketing and functional morphology (e.g. *Echovenator*, *Cotylocara*) [16, 17], with important contributions from functional complexes of living species. To consider directions for research on Oligocene Neoceti, substantial fossil collections from long-recognised productive regions (e.g. South Carolina, coastal Oregon to British Columbia, Hokkaido, New Zealand) include many undescribed taxa. Most fossil Neoceti are from temperate latitudes, with equatorial and polar fossils barely known (e.g. *Arktocara*) [18]. A persistent issue is the difficulty of dating fossil Cetacea; marine biozones or absolute radiometric dates are rarely available or cited.

**Fig 1:**
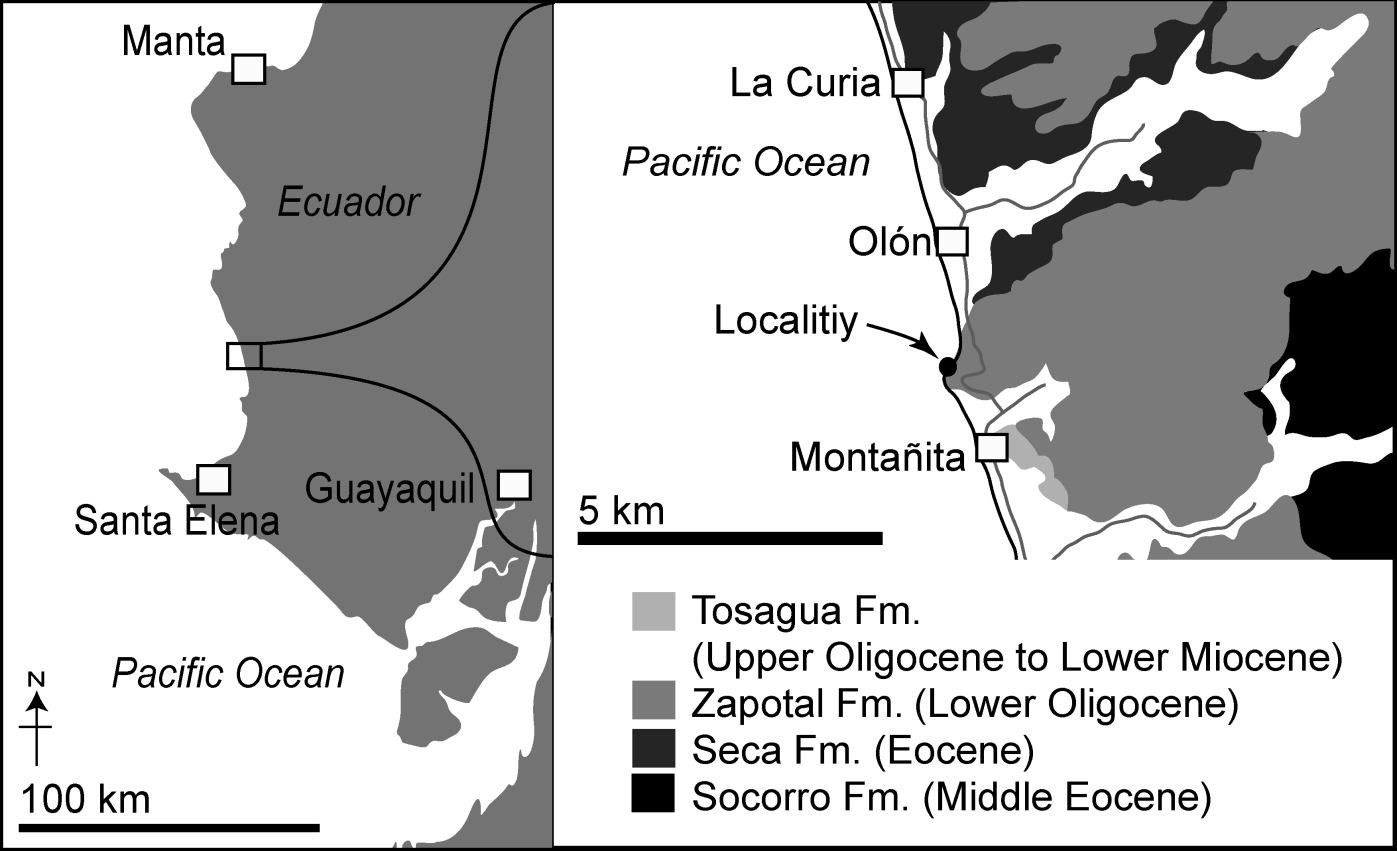
Locality maps of *Urkudelphis chawpipacha* MO-1 (holotype) from Montañita/Olón, Santa Elena, Ecuador, modified from the geological map of Aguilera et al (1974).

Here, we describe a small dolphin skull that represents a young calf from Santa Elena Province, Ecuador (Fig 1), on the margin of the eastern tropical Pacific. Enough detail is preserved, especially for the ear region, to diagnose and name the specimen as a new genus and species. Phylogenetic analysis places it near the base of the almost extinct clade Platanistoidea. The fossil is one of a few cetaceans from the tropical eastern Pacific margin. Hitherto, previously-reported cetacean remains from Ecuador include a fragmentary ziphiid specimen (ear bones and small pieces of mandibles) and an isolated tooth possibly belonging to a ziphiid, from the Early-Middle Miocene [19] and cetacean ribs from the Late Pliocene to Pleistocene [20].

## Materials and methods

### Acronyms

**MO–**Montañita/Olón collection, at Universidad Estatal Peninsula de Santa Elena (**UPSE**), Ecuador; **OU–**Geology Museum, University of Otago, Dunedin, New Zealand.

### Material

Skull MO-1 was prepared by one of the authors (JA) using an electric hand-drilling tool with a fine grinding tip to remove matrix from the bone, viewed with a stereo-microscope. Morphological terminology follows Mead and Fordyce [21] unless stated.

### Ethics statement

The fossils were excavated with permission from the Instituto Nacional de Patrimonio Cultural: permission code N°.040.DR5.INPC.2015.

### Nomenclatural acts

The electronic edition of this article conforms to the requirements of the amended International Code of Zoological Nomenclature, and hence the new names contained herein are available under that Code from the electronic edition of this article. This published work and the nomenclatural acts it contains have been registered in ZooBank, the online registration system for the ICZN. The ZooBank LSIDs (Life Science Identifiers) can be identified and the associated information viewed through any standard web browser by appending the LSID to the prefix "http://zoobank.org/". The LSID for this publication is: urn:lsid:zoobank.org:pub:66F609D8-588D-4622-910E-E8C2A01D317B. The electronic edition of this work was published in a journal with an ISSN, and has been archived and is available from the following digital repositories: PubMed Central, LOCKSS.

### Systematic paleontology

CETACEA Brisson, 1762

NEOCETI Fordyce & de Muizon, 2001

ODONTOCETI Flower, 1867

PLATANISTOIDEA Gray, 1863 sensu Fordyce, 1994

### Comment

*Urkudelphis chawpipacha* shows these synapomorphies of the Platanistoidea (sensu Fordyce, 1994), as recognised previously by Tanaka and Fordyce [22]: periotic with C-shaped parabullary sulcus; small articular rim, which forms a ridge anterolateral to posterior process of periotic and separated from it by a sulcus. Two phylogenies place *Urkudelphis* near the base of the Platanistoidea (sensu lato; including Platanistidae, *Squalodelphis*, *Notocetus*, *Phocageneus*, *Otekaikea*, Waipatiidae, *Awamokoa* and Squalodontidae [22]).

*Urkudelphis* gen. nov.

urn:lsid:zoobank.org:act:F49218A8-3BC0-4C18-A0E6-78B2B39D0A4F

Type species:*Urkudelphis chawpipacha* sp. nov.

Diagnosis. As for the type species, below.

*Urkudelphis chawpipacha* sp. nov.

urn:lsid:zoobank.org:act:40DFB6BF-CDC7-41AD-8FFC-A51016BA6234

(Figs 2–12, Tables 1 and 2)

**Fig 2:**
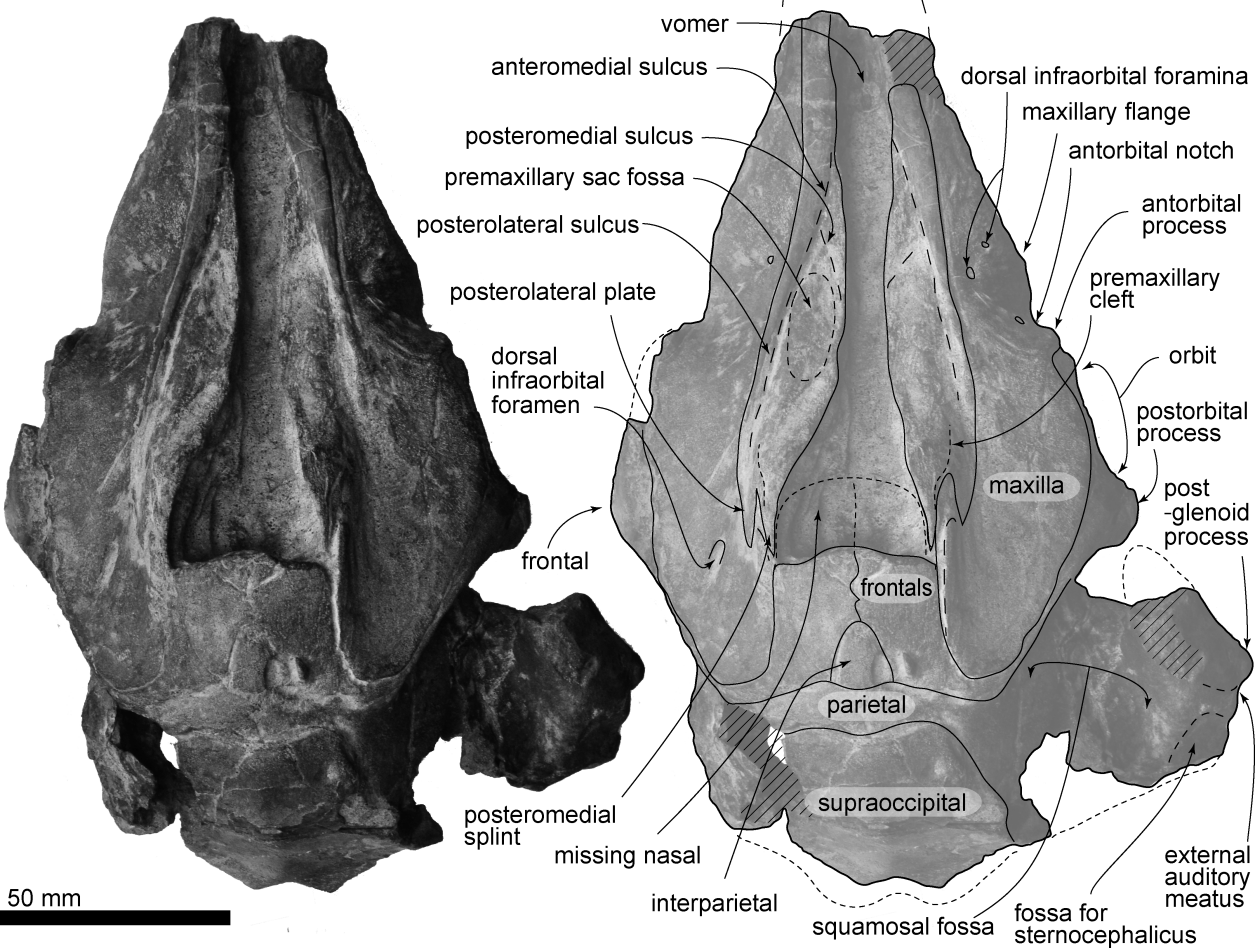
Skull, *Urkudelphis chawpipacha* MO-1 (holotype) in dorsal view. Left, photo, right, line art.

**Fig 3:**
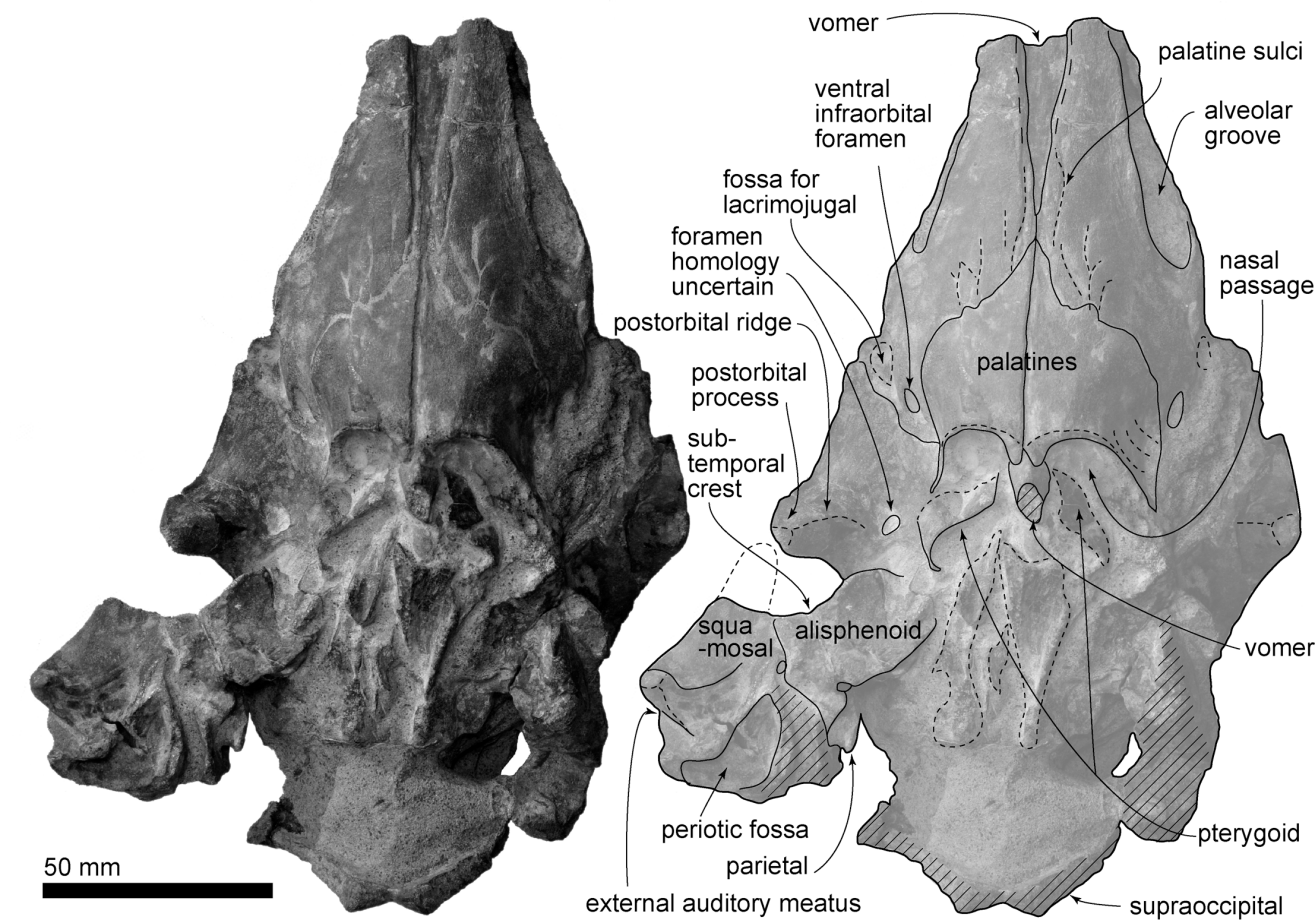
Skull, *Urkudelphis chawpipacha* MO-1 (holotype) in ventral view. Left, photo, right, line art.

**Fig 4:**
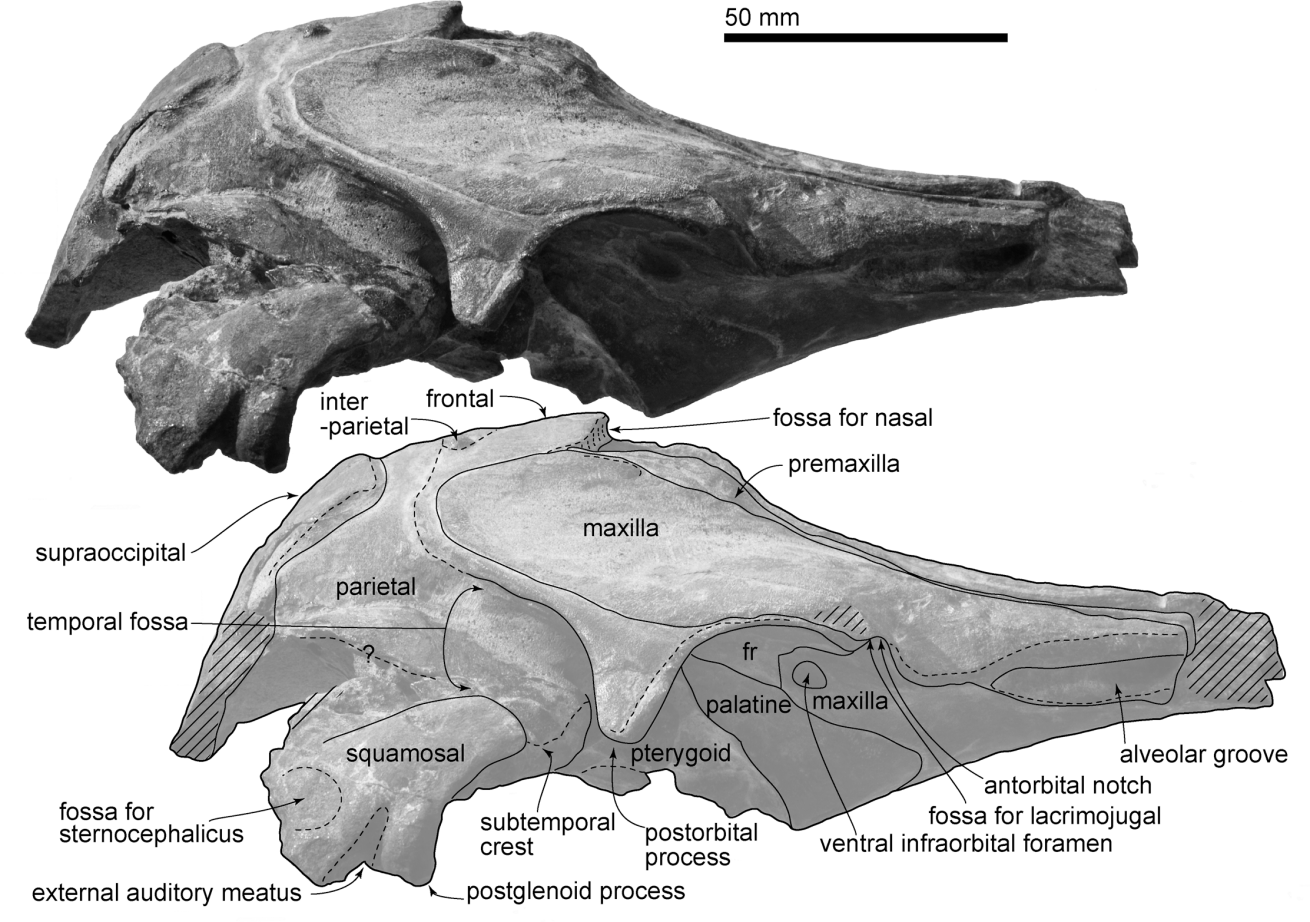
Skull, *Urkudelphis chawpipacha* MO-1 (holotype) in right lateral view. Upper, photo, lower, line art.

**Fig 5:**
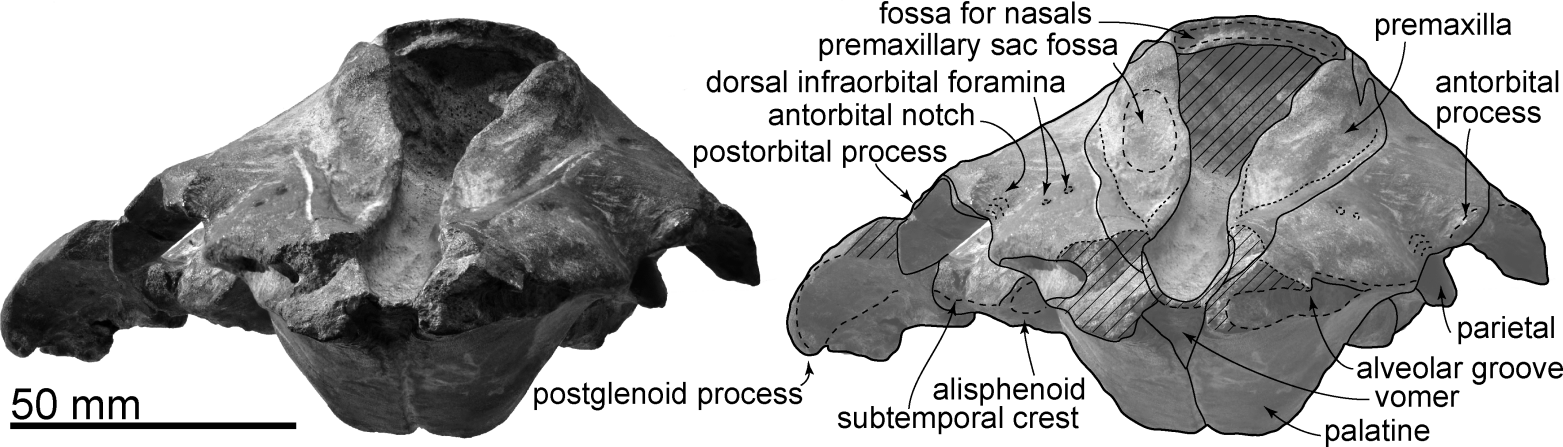
Skull, *Urkudelphis chawpipacha* MO-1 (holotype) in anterior view. Left, photo, right, line art.

**Fig 6:**
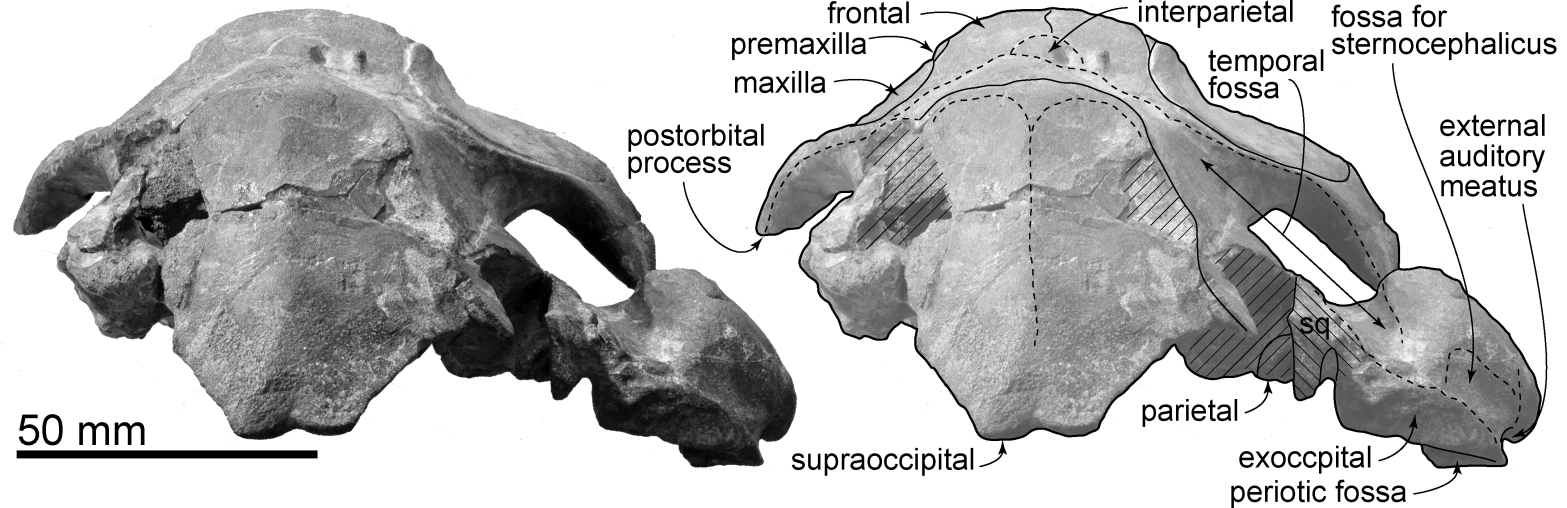
Skull, *Urkudelphis chawpipacha* MO-1 (holotype) in posterior view. Left, photo, right, line art.

**Fig 7:**
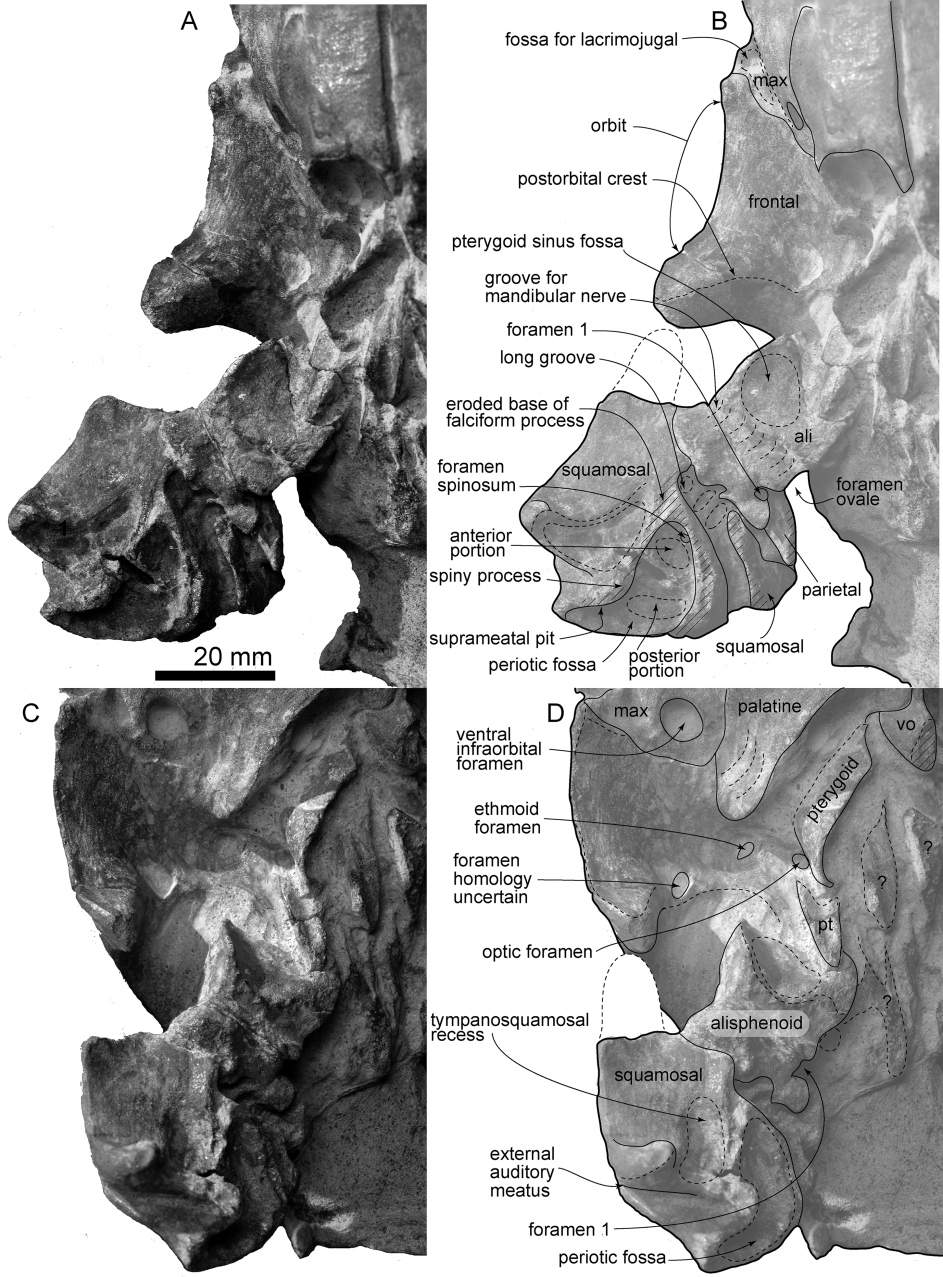
Details of the right basicranium, *Urkudelphis chawpipacha* MO-1 (holotype). (A) and (B) ventral view; (C) and (D) slightly lateral-ventral view. (A) and (C) photo, (B) and (D) line art.

**Fig 8:**
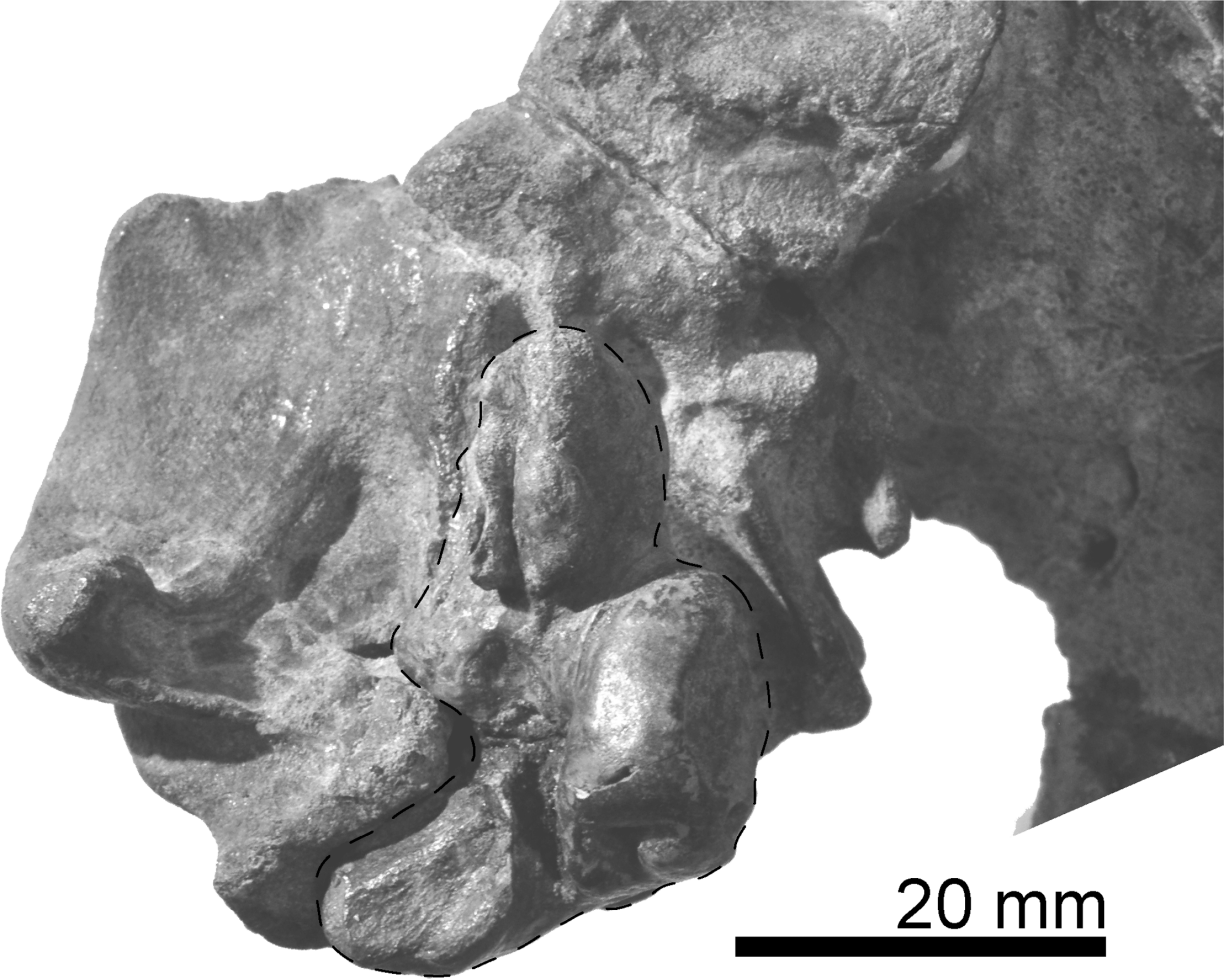
Right periotic, *Urkudelphis chawpipacha* MO-1 (holotype) *in situ*, in ventral view.

**Fig 9:**
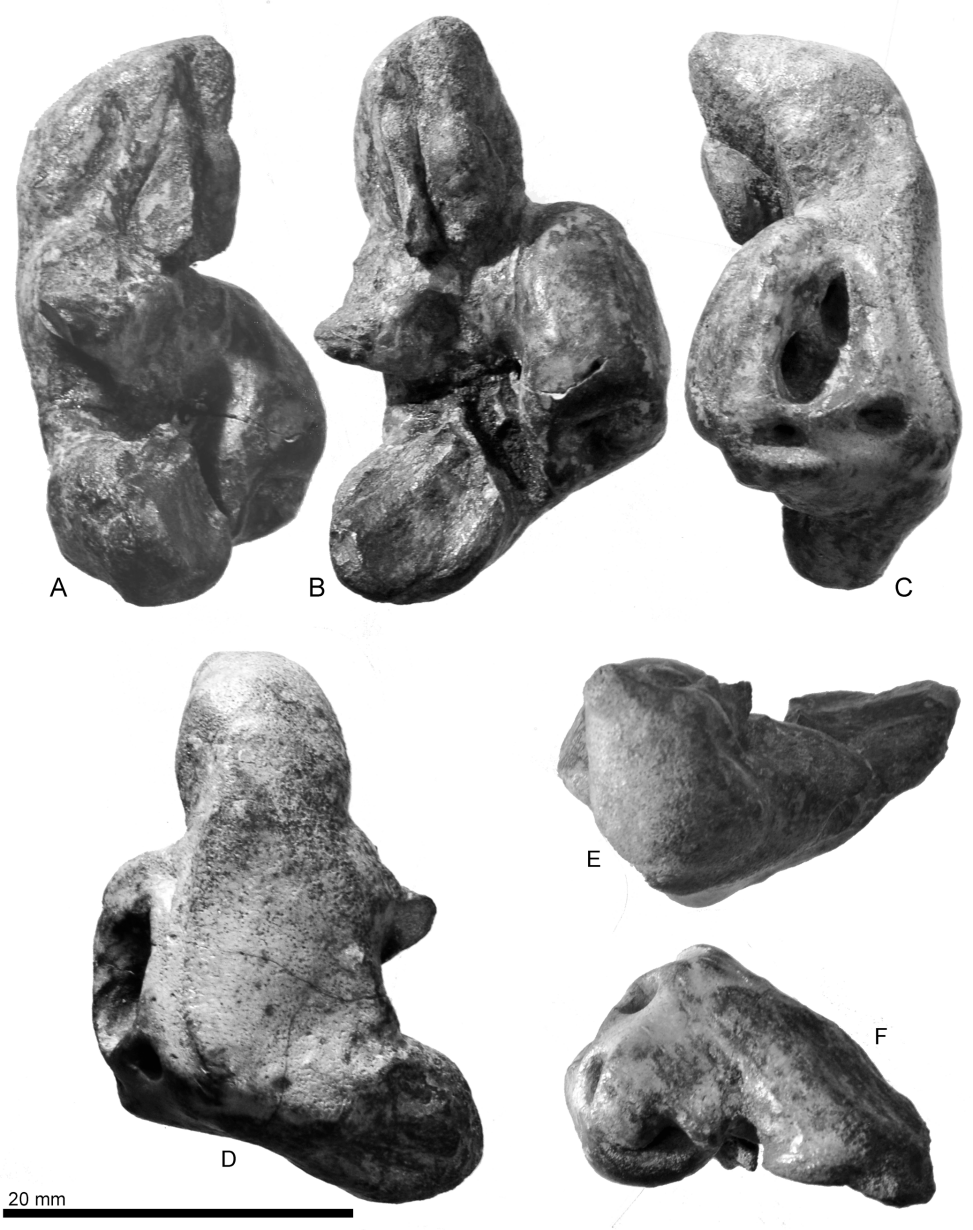
Right periotic of *Urkudelphis chawpipacha*, MO-1 (holotype). (A) Lateral view. (B) Ventral view. (C) Medial view. (D) Dorsal view. (E) Anterior view. (F) Posterior view.

**Fig 10:**
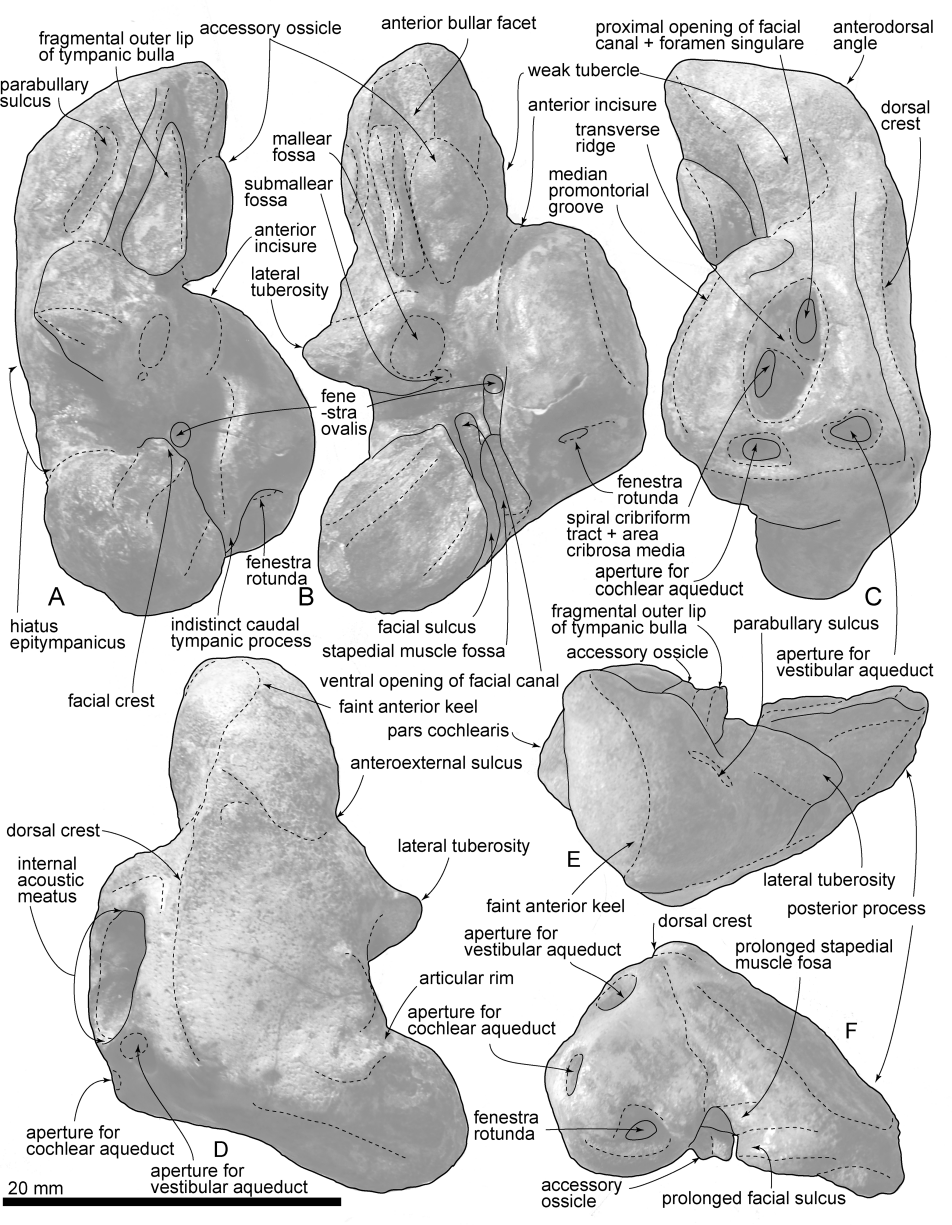
Key features of the right periotic of *Urkudelphis chawpipacha*, MO-1 (holotype). (A) Lateral view. (B) Ventral view. (C) Medial view. (D) Dorsal view. (E) Anterior view. (F) Posterior view.

**Fig 11:**
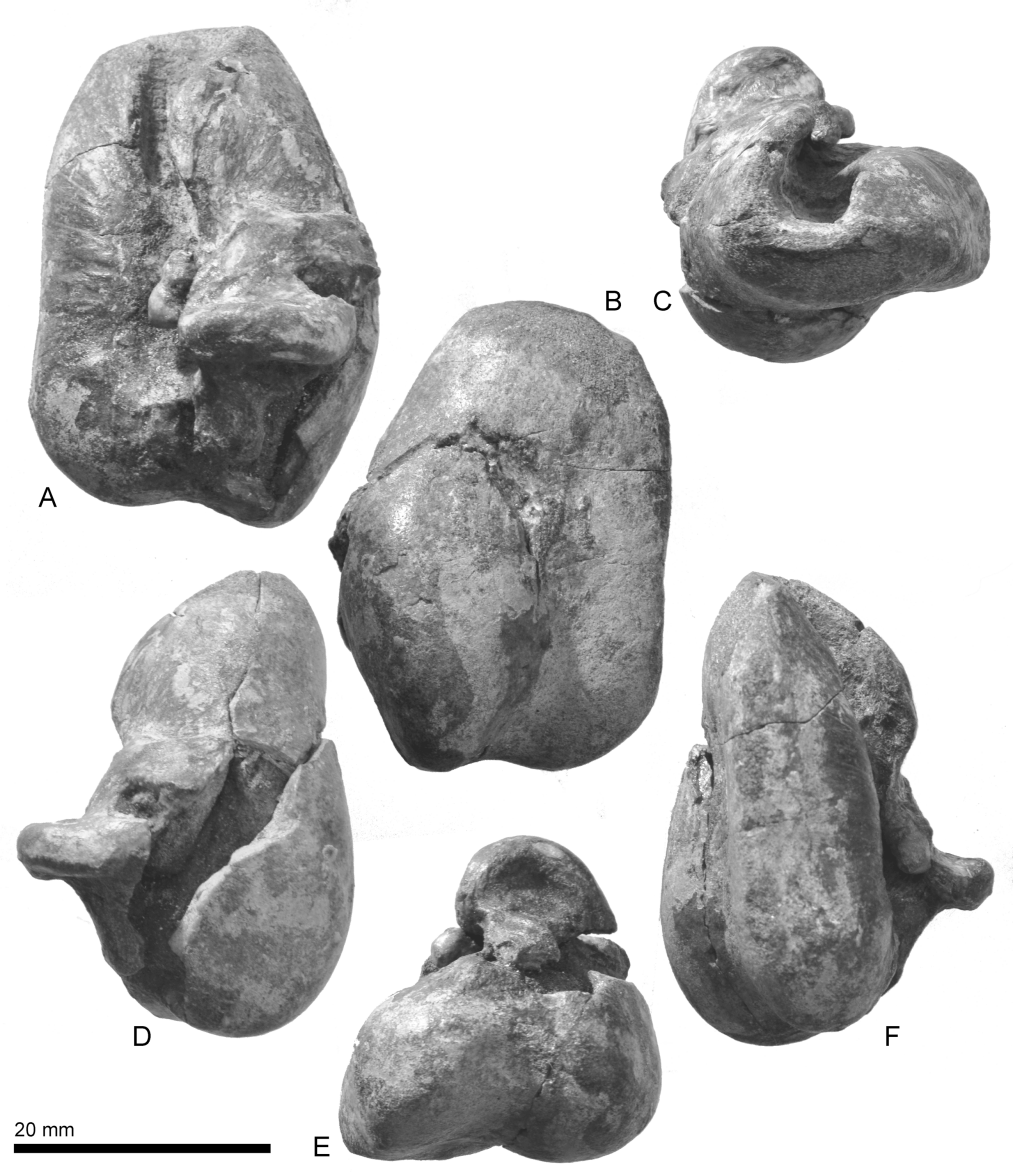
Right tympanic bulla of *Urkudelphis chawpipacha*, MO-1 (holotype). (A) Dorsal view. (B) Ventral view. (C) Anterior view. (D) Lateral view. (E) Posterior view. (F) Medial view.

**Fig 12:**
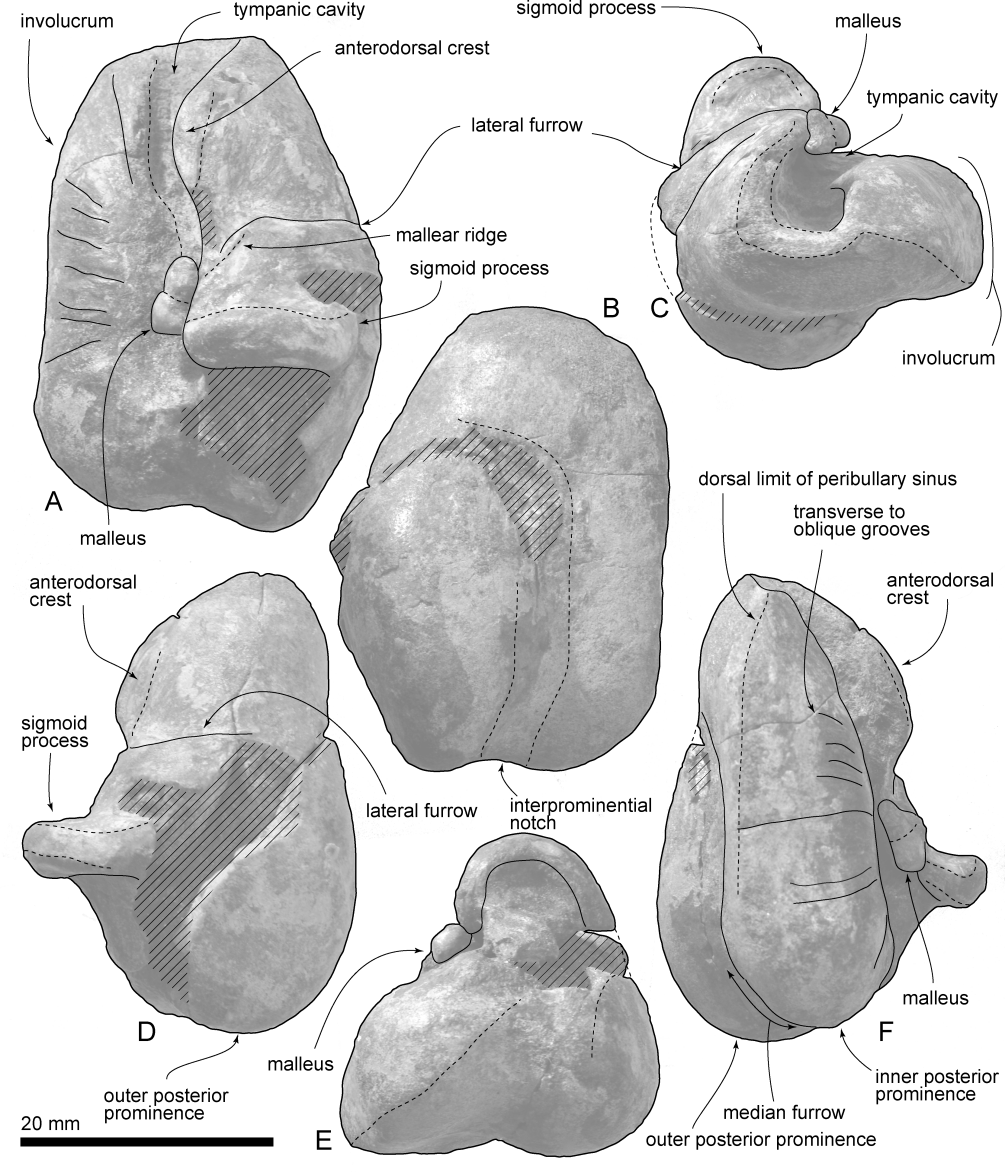
Key features of the right tympanic bulla of *Urkudelphis chawpipacha*, MO-1 (holotype). (A) Dorsal view. (B) Ventral view. (C) Anterior view. (D) Lateral view. (E) Posterior view. (F) Medial view.

**Table 1:** Measurements in mm of *Urkudelphis chawpipacha*, MO-1 (holotype) skull. Dimensions follow [14]. Measurements are rounded to the nearest 0.5 mm. For the skull, distances are either horizontal or vertical.

Skull	Measurement (mm)
Total length, between the most anterior and posterior points	191.5+
Cranial maximum preserved length	129.5+
Width of premaxillae at a line across posterior limits of antorbital notches	41.5
Maximum width of premaxillae about the level with mid-orbit	48.5
Postorbital width, across apices of postorbital processes	113.5
Median length of frontals on vertex	15.0
Anteroposterior diameter of right temporal fossa proper	63.0
Vertical external height of skull, from most ventral part of braincase on basioccipital crest, to dorsal extremity of frontal at vertex	62.0
Zygomatic width from median line	86.0

**Table 2:** Measurements in mm of MO-1 (holotype) right periotic and tympanic bulla. Dimensions follow [14]. Measurements are rounded to the nearest 0.5 mm.

Periotic	Measurement (mm)
Maximum anteroposterior length, from anterior apex of anterior process to apex of posterior process	34.5
Maximum anteroposterior length parallel to dorsal margin	33.5
Maximum dorsoventral depth anterior process, perpendicular to axis of periotic	13.0
Length of anterior process, from anterior apex to level of posterior of mallear fossa	20.5
Length of anterior process, from anterior apex of anterior process to level of anterior of pars cochlearis	13.5
Length of posterior bullar facet, point to point	12.0
Maximum mediolateral width of anterior process at base	10.0
Approximate anteroposterior length of pars cochlearis, from anterior incisure to caudal process	17.5
Approximate transverse width of pars cochlearis, from internal edge to fenestra ovalis	8.5
Transverse width of periotic, internal face of pars cochlearis to apex of lateral tuberosity	20.5
Length of posterior process of periotic	11.0
Length of posterior process parallel to posterior profile/ steeply acute to long axis of body	11.0
**Tympanic bulla**	
Standard length anterior apex to apex of outer posterior prominence	39.5
Length anterior apex to apex of inner posterior prominence	38.5
Maximum width	26.5
Approximate width between inner and outer posterior prominences	13.0
Dorsoventral depth of involucrum immediately in front of posterior pedicle	13.0
Width of sigmoid process	13.5
Height of sigmoid process	8.5

Holotype: MO-1, an incomplete skull (premaxilla, maxilla, vomer, pterygoid, frontal, parietal, interparietal, alisphenoid, squamosal and supraoccipital), including the right periotic, right tympanic bulla and right malleus.

### Etymology

The generic name, *Urkudelphis* originates from Kichwa “urku” meaning mountain, referring to the type locality of Montañita, and Greek “delphis” for dolphin, which has been used widely as a suffix for dolphin generic names. Chawpipacha results from the combination of chawpi, meaning "half" or “middle” and pacha, meaning "the world" representing the equator, and thus Ecuador in Kichwa.

### Type locality

MO-1 was found by one of the authors (JA) and several UPSE students in August 2015 in a boulder that collapsed from a cliff at the coastal locality here named Montañita/Olón (latitude 1°48'50.64" S, longitude 80°45'24.18" W). The Montañita/Olón (MO) locality (Fig 1) lies midway between the towns of Montañita and Olón (Santa Elena Province, Ecuador) and can only be accessed during low tides.

### Type horizon and age

The dolphin-bearing strata at the Montañita/Olón fossil locality are composed of a moderately-sorted, fine to medium sandstone with angular quartzo-feldspathic clasts. Conspicuous rounded green grains are probably glauconite, but berthierine cannot be dismissed. The matrix is micritic and volcanogenic, possibly bentonitic. Bedding is massive to indistinct, suggesting little influence by traction currents or storm waves, and in turn implying a quiet setting; estuarine or mid-shelf is possible. Rare benthic foraminifera, and a lack of planktic foraminifera, suggest an estuarine rather than a shelf environment. The sediments exposed in lower levels of the cliffs of Montañita/Olón are rich in fossil vertebrates (mostly cetaceans and sharks, including teeth of *Carcharocles angustidens*), whereas bivalves and gastropods are more abundant in the upper levels of the section. The fossils are consistent with an Oligocene age, discussed below.

The published litho- and chronostratigraphy of the cetacean-bearing sediments exposed at the Montañita/Olón fossil locality is complicated. Different geologists have used different stratigraphic names derived from several nearby regional basins and/or fault-bounded sequences, and we give only a simple summary here. The Montañita/Olón strata were reported [23] as part of the Zapotal Formation (sensu Olsson [24]). "Zapotal" is one of nine lithostratigraphic names for what may be the same unit ([23]:129). Whittaker [25] noted errors in the key geological map including the Montañita/Olón locality [26], leading him to recognise most of the Zapotal Formation as the Progreso Formation. Whittaker [25] also questioned the stratigraphic relationships of the Manglaralto-Montañita area and the depositionally-separate main Progreso Basin to the south.

To consider the age of the cetacean-bearing site, Olsson [24] reported the benthic mollusks *Thyasira montanita* and *Epitonium* cf. *antiguense* at Montañita/Olón and assigned these sediments a "middle" Oligocene (Rupelian?) age based on the mollusks. Later, Bristow [23] identified the Montañita/Olón strata as part of the Zapotal Member of the Tosagua Formation and of Chattian–Aquitanian age, reporting the presence of the supposedly Miocene nautiloid *Aturia curvilineata*. Nielsen et al. [27], however, listed *A*. *curvilineata* as a junior synonym for the Eocene-Miocene nautiloid *A*. *cubaensis*. Whittaker [25] used the name Dos Bocas Formation, rather than Tosagua Formation, because Dos Bocas was originally used for reportedly Early Miocene strata of the neighboring Manabí Basin. Benítez [28] suggested that the sandstone at Montañita/Olón belongs to the Consuelo Formation, which he interpreted as late Burdigalian to early Langhian in age based on its stratigraphic position between the Villingota and Subibaja Formations. Benítez's description of lithostratigraphy is difficult to follow, and we are unsure which of his formations or ages best fits the cetacean horizon.

Here, we provisionally identify the source horizon for MO-1 as the Zapotal Member of the Dos Bocas Formation, with names following Whittaker [25]. More study is needed to establish the proper terminology of the coastal strata outside, but closely-related to, the Progreso Basin. We did not find foraminiferans that might be used for dating. The presence of *Carcharocles angustidens* is consistent with a late Oligocene age suggested by Bristow. Elsewhere, in the East Pisco basin of Peru, *C*. *angustidens* is not reported from the vertebrate-bearing Chattian to Burdigalian Chilcatay Formation, in which the richest vertebrate-bearing horizon is Burdigalian [29–31]), but occurs in older units (M. Urbina and A. Altamirano (Departamento de Paleontología de Vertebrados, Museo de Historia Natural, Universidad Nacional Mayor de San Marcos), personal communication, fide T.J. DeVries (Burke Museum of Natural History and Culture, University of Washington, Seattle)). Thus, the age of MO-1 is consistent with a probable Chattian age (24 to 26 Ma), as shown by Bristow [23] (his Fig 3, Zapotal Member of Tosagua Formation).

### Diagnosis

*Urkudelphis chawpipacha* is a small archaic odontocete with the following autapomorphic combination of characters: shallow antorbital notch (character 10); anteromedially oriented anterior edge of the supraorbital process (character 37); weakly dorsally convex nuchal crest in dorsoposterior view (character 119); approximately same sized apertures of the vestibular aqueduct and cochlear aqueduct (character 186); dorsoventrally thin pars cochlearis on the periotic (character 192); inner posterior prominence of the tympanic bulla is anterior to the outer posterior prominence (character 218); very strongly projecting and pointed lateral tuberosity; and an anteroposteriorly long accessory ossicle of the periotic. *Urkudelphis chawpipacha* differs from early branching odontocetes, including *Agorophius*, *Ashleycetus*, *Simocetus*, *Mirocetus* and *Xenorophus* in having the frontals on the vertex at a level behind the postorbital process; anteroposteriorly shorter and transversely wider frontals (approaching a square-shape rather than narrow and elongate); and parallel-sided posterior part of the ascending process of each maxilla forming a narrow elongate face. *Urkudelphis* differs from Early Miocene *Papahu taitapu*, *Chilcacetus cavirhinus*, *Arktocara yakataga*, *Allodelphis pratti* and *Ninjadelphis ujiharai*, having the frontals on the vertex flat and longer than the taxa above, which have more nodular and shorter frontals. *Urkudelphis* differs from *Chilcacetus* and *Papahu* in having a narrow premaxillary sac fossa. *Urkudelphis chawpipacha* also notably shows: frontals at the vertex invaded posteriorly by the interparietal; and long anteromedial projection of the palatine on the palate. Other diagnostic features of *U*. *chawpipacha* are shared with more-crownward Waipatiidae: a shallow suprameatal pit of the squamosal (character 152); an abruptly ventrally deflected anterior process of the periotic (character 172); and a nearly flat dorsal surface of the periotic in lateral view (character 181). In addition, *Urkudelphis chawpipacha* shares several characters with more-crownward Platanistoidea: a periotic with C-shaped parabullary sulcus (character 175); and a small articular rim, which forms a ridge anterolateral to the posterior process of the periotic and separated from it by a sulcus (character 195).

### Description

#### Ontogenetic age

Several features indicate that MO-1 is juvenile. Those skull sutures that are cleanly exposed are open and distinct. There is a distinct interparietal [21], and an incomplete supraoccipital with partly developed margins and lacking nuchal crests. A prominent large upper alveolar groove lacks distinct alveoli, as seen in newborn extant species of *Stenella* (Delphinidae) [32], consistent with suckling but not feeding with the aid of large-rooted teeth (teeth were presumably lost post-mortem).

#### Skull and body size

The reconstructed bizygomatic width (172 mm) using the preserved, albeit distorted, right side (86 mm) of MO-1 is about 66% of the size of the well preserved holotype of *Waipatia maerewhenua*, which is an adult or subadult [11]. The preserved cranium is 191.5 mm long, from the antorbital notch to the posterior margin of the supraoccipital (the condyles are lost). The incomplete rostrum is 71.5 mm long from its broken apex to its base at the antorbital notch.

The body size of MO-1 can be estimated using the Pyenson and Sponberg [33] formula for stem Platanistoidea: Log(L) = 0.92* (log(BIZYG)-1.51)+2.49 (see also S1 File). The reconstructed body length of MO-1 is 1.7 m.

#### Cranial topology

The skull preserves most bones on one or both sides, except for the basicranium which is crushed behind the palatine, with a better preserved right margin. There is some distortion from burial (as evident e.g. from the mesorostral groove and outline of the bony nares), but no clear evidence of original directional asymmetry. The skull is triangular in dorsoventral view, with the almost straight right rostral and orbital margins slightly interrupted by a small antorbital notch. The right zygomatic process and ear region are slightly raised by burial deformation.

#### Premaxilla

The broken anterior cross section of each premaxilla of the rostrum shows a dorsally flattened and ventrally pointed triangle. At the level of the antorbital notch, a dorsally convex premaxillary sac fossa (Fig 2) is restricted laterally by a deep groove comprising the shorter anteromedial and longer posterolateral sulcus; there is an indistinct shallow posteromedial sulcus. Surfaces are not preserved well enough here to show a distinct nasal plug muscle fossa, and the premaxillary foramen cannot be identified. Lateral to the anteromedial and posterolateral sulci, the premaxilla has a smooth elevated porcelanous part, parallel-sided anteriorly, and passing back into a narrow ridge. Posterior to the premaxillary sac fossa, the premaxilla rises smoothly then abruptly at the level of the premaxillary sac fossa. The posterior end of the premaxilla has a slightly longer posteromedial splint with a deep premaxillary cleft on the dorsal surface, and a shorter posterolateral plate (Fig 2). In dorsal view, the premaxilla probably sutured with the nasal, which is now missing on MO-1, and the premaxilla does not contact the frontal.

#### Maxilla

The maxilla forms an anteriorly narrow triangular rostral part, and a wider cranial or facial part. The rostrum has a gently convex bilateral maxillary flange, just anterior to a shallow antorbital notch and incipient antorbital process. The surface of the ascending process is a smooth, narrow, long, curved portion of maxilla that rises gradually at the level of the bony nares to cover most of the frontal, but does not cover the margin of the orbit, or antorbital, or postorbital processes. The ascending process rises steeply posteromedially, and does not reach the interparietal, parietal, and supraoccipital. At the level of the antorbital notch, there are small dorsal infraorbital foramina; three on the right and one on the left. The posterior dorsal infraorbital foramen opens lateral to the vertex.

In ventral view (Fig 3), lateral to the alveolar groove, the rostrum margin is blunt. The alveolar groove is widely open (9.0 mm maximum width, 52.5+ mm length), but lacks distinct alveoli. The posterior end of the alveolar groove is at the maxillary flange. Several palatine sulci run anteriorly from the maxillopalatine suture or just anterior to it. Just posterior to the antorbital notch, an L-shaped, small and deep fossa for the lacrimojugal is located along the margin of the maxilla; however, the lacrimojugal is missing. Posteromedial to the fossa for the lacrimojugal, a large elliptical ventral infraorbital foramen (8.8 mm long, 7.0 mm wide) (Figs 3 and 4) opens at the posterior part of the maxilla, without contribution from the frontal.

#### Vomer

The vomer in the mesorostral groove has a widely opened U-shape section in anterior view (Fig 5). Ventrally, the vomer is exposed anteromedially, as a long narrow triangle reaching back to within 7 mm of the palatine. The preserved posterior end of the vomer appears posteromedial to the palatines, with a dorsoventrally long elliptical broken section. The vomer is thickened posteriorly, but is obscured by matrix in the narial area, and lost further posteriorly.

#### Palatine

The ventral surface of the palatine is transversely convex (Fig 3), with a smooth surface and an anteromedial process that overlaps the posterior part of the maxilla. Each palatine forms the anteroventral border of the internal bony naris and a contact with the pterygoid. The internal bony nares are visible where the pterygoids are lost. The lateral lamina has a shallow depression on the surface, possibly for the *m*. *pterygoideus externus* insertion [34]. The sphenopalatine foramen could not be identified.

#### Pterygoid

Both the pterygoids are displaced and mostly lost. Only the medial lamina is visible, as a laterally rounded plate (Fig 3). The hamulus, eustachian notch, medial lamina, and contact if any with the falciform process on the squamosal are lost.

#### Frontal

Dorsally, the frontal is medially covered by the maxilla (Fig 2). The frontal forms the blunt triangular postorbital process, which projects posteroventrally. Posterior to the postorbital process, the frontal forms the roof of the temporal fossa (Figs 3 and 6), and is exposed dorsally where it continues to the vertex. The maxilla and supraoccipital are thus separated by the frontal and the parietal. On the vertex, the dorsal surface of the joined frontals are dorsally flat and large, approaching a square profile. There is a sinuous interfrontal suture (Fig 2). Here, in dorsal view, the anterior border of the frontal at the center is slightly convex anteriorly. The anterior edge of each frontal (Figs 4 and 5) has a narrow groove (30.5 mm bilateral width, 2.5 mm high), forming the suture for the lost nasals. The nasals were, therefore, probably thin plates that roofed the nares, unsupported ventrally by the frontals.

Ventrally (Figs 3 and 4), the frontal forms the roof of the orbit. The orbital region is bounded by a distinct postorbital ridge, but there is no preorbital ridge. The orbital region has a rounded ethmoid foramen medially (3.5 mm diameter; Fig 7). Medial to the ethmoid foramen, there is a rounded optic foramen. Another foramen (see Fig 7 bottom) of uncertain homology opens between the postorbital process and the optic canal. The orbitosphenoid is not distinct. The frontal does not contribute to the lacrimojugal fossa, which is formed only by the maxilla.

#### Parietal and interparietal

Dorsally, the parietal is an anteroposteriorly narrow band, raised medially, just between the frontal and supraoccipital. The rounded triangular interparietal sits anterior to the parietal medially, and projects into the posterior half of the frontals. Laterally, the weakly swollen parietal forms the medial surface of the temporal fossa.

#### Alisphenoid

The square alisphenoid lies posterior to the frontal and anteromedial to the squamosal on the ventral side of the skull (Figs 3 and 7), forming the anteromedial margin of the subtemporal crest. Posterolaterally, the alisphenoid contacts the squamosal. A large round pterygoid sinus fossa (17.0 mm diameter) sits anteromedially and posterior to the subtemporal crest. Lateral to the pterygoid sinus fossa, a faint groove for the mandibular nerve obliquely crosses the alisphenoid from its origin at the semicircular foramen ovale. A small "foramen 1" (3.0 mm diameter) opens lateral to foramen ovale. It is uncertain whether the foramen ovale was separated from the cranial hiatus by a bony ridge.

#### Squamosal

The squamosal has an anteroposteriorly thin postglenoid process, and deeply excavated mandibular fossa and external auditory meatus (Figs 4 and 7). The fossa for the sternocephalicus [35] (= "neck muscle fossa" sensu (Fordyce [36]) is shallow and high (14.5 mm high, 11.5 mm long). The dorsolateral edge of the zygomatic process is angular, with a clear border between the lateral and dorsal surfaces of the squamosal.

Ventrally (Fig 7), the squamosal shows a triangular tympanosquamosal recess, anteromedial to the postglenoid process. Posteromedial and dorsal to the recess, a triangular and deep periotic fossa is divided into anterior and posterior portions, with the anteromedial margin of the periotic fossa showing a small rounded foramen spinosum (1.0 mm diameter). The falciform process is mostly lost, but its narrow sinuous base remains. There is no obvious sigmoid fossa for the sigmoid process of the tympanic bulla. The worn spiny process is preserved just medial to the widely posteroventrally open external auditory meatus. The anterior part of the alisphenoid-squamosal suture is clear at the base of the zygomatic process and subtemporal crest. The posterior part of the suture is a broken and ventrally open long groove with small depressions on the squamosal.

#### Supraoccipital

The supraoccipital is ossified, without evident fontanelles, but has a partly open suture with the parietal, and shows weakly angled margins. In posterior view (Fig 6), the supraoccipital is a dorsally-narrow trapezoid. The median part of the dorsal margin of the supraoccipital is slightly depressed. An incipient external occipital crest is present more posteriorly.

#### Other basicranial elements

The exoccipital is not preserved. The basioccipital and basisphenoid may be preserved as fragments, but cannot be identified.

#### Periotic

The right periotic (Figs 8–10) has a slender anterior process, shorter and more robust posterior process, and a dorsoventrally weakly inflated pars cochlearis. The axes of the anterior and posterior processes make a wide angle (about 120°). The periotic is separated from the skull, but can be inserted neatly into its original position in the periotic fossa (Fig 8).

The slender anterior process has a blunt apex and faint anterior keel, which continues from the apex to the dorsal crest. The anterior process bends ventrally and shows a strong anterodorsal angle, as seen in *Waipatia maerewhenua* and *Otekaikea* spp. The parabullary sulcus [13] is shallow with a weakly curved C-shape. A faint anteroexternal sulcus runs from near the posterior end of the parabullary sulcus to the mediodorsal side of the periotic. The anterior bullar facet is shallow with parallel margins (3.0 mm wide). The fovea epitubaria is occupied by an anteroposteriorly long accessory ossicle (8.3 mm long). Lateral to the ossicle is a thin flange, which is a fragment of the outer lip of the tympanic bulla. The ossicle is elliptical and dorsoventrally flattened. Just posteromedial to the accessory ossicle, an open rounded anterior incisure separates the anterior process from the pars cochlearis.

The anteroposteriorly long elliptical pars cochlearis is anteriorly narrower and posteriorly wider in ventral view. An anteroposteriorly long, elliptical, internal auditory meatus opens medially (8.8 mm maximum length). The anterior region (proximal opening of the facial canal + foramen singulare) is narrower (4.0 mm length, 1.4 mm maximum width), than the more-rounded posterior region (the spiral cribriform tract + area cribrosa media) (4.4 mm length, 3.8 mm maximum width). The fenestra rotunda is a transversely wide elliptical opening (transverse diameter 3.2 mm, 2.2 maximum length). The aperture for the cochlear aqueduct is transversely wider, subcircular, and small (2.3 mm width, 1.4 mm length). The aperture for the vestibular aqueduct is slightly larger (2.6 mm width, 2.0 mm length) and the same shape as the opening for the cochlear aqueduct. A shallow median promontorial groove runs anteroposteriorly just ventral to the internal acoustic meatus. The caudal tympanic process is indistinct.

The lateral tuberosity projects strongly, and its lateral end is pointed. The rounded and large mallear fossa (5.5 mm diameter) is located medial to the lateral tuberosity. At the most posteromedial margin of the mallear fossa, there is a very small submallear fossa [22] (0.7 mm diameter). Posterior to the mallear fossa, a rounded fenestra ovalis (1.8 mm diameter) opens at the edge of the pars cochlearis. Adjacent are the ventral opening of the facial canal and facial sulcus, lateral to the fenestra ovalis. The facial sulcus and stapedial fossa are prolonged posteriorly, and become indistinct on the medial face of the posterior process.

In ventral view, the posterior bullar facet is trapezoidal and wide (10.6 mm long, 10.2 mm wide), with a rounded apex. The articular rim is blunt and locates on the dorsal side (Fig 10D). The ventral surface of the posterior bullar facet has three sections separated by weak ridges.

#### Tympanic bulla

The right tympanic bulla of *Urkudelphis* (Figs 11 and 12) is heart-shaped in ventral view, bilobed posteriorly. Its lateral margin is markedly convex, giving a maximum width level between the lateral furrow and sigmoid process. The medial margin is straight to faintly concave. The anterior margin of the bulla is bluntly rounded, without the anterior spine as in *Platanista* or a spout-like incipient spine as in *Waipatia maerewhenua* (see [11]). The posterior part of the medial margin of the involucrum is straight. The involucrum has transverse to oblique grooves on the ventral to medial surfaces. The ventral surface of the involucrum has a longitudinal weak ridge, interpreted as the ventral limit of the peribullary sinus. The anterodorsal crest is the thick margin of the strongly curved outer lip, which projects medially to roof much of the tympanic cavity (8.6 mm high, 6.4 mm wide). Posterior to the anterodorsal crest, a marked lateral furrow descends vertically, separating the gently curved anterior of the outer lip from the more-inflated base of the sigmoid process. At the dorsal limit of the furrow, an oblique fine ridge is probably the fused anterior process of the malleus. The sigmoid process is anteroposteriorly thick (4.8 mm), and semicircular in anteroposterior views with a rounded lip; there are no obvious facets for a sigmoid fossa or for contact with the lateral tuberosity of the periotic. Posteriorly, a shallow interprominential notch passes forward into a median furrow. The outer posterior prominence is longer and more rounded than the inner posterior prominence.

#### Malleus

**Fig 13:**
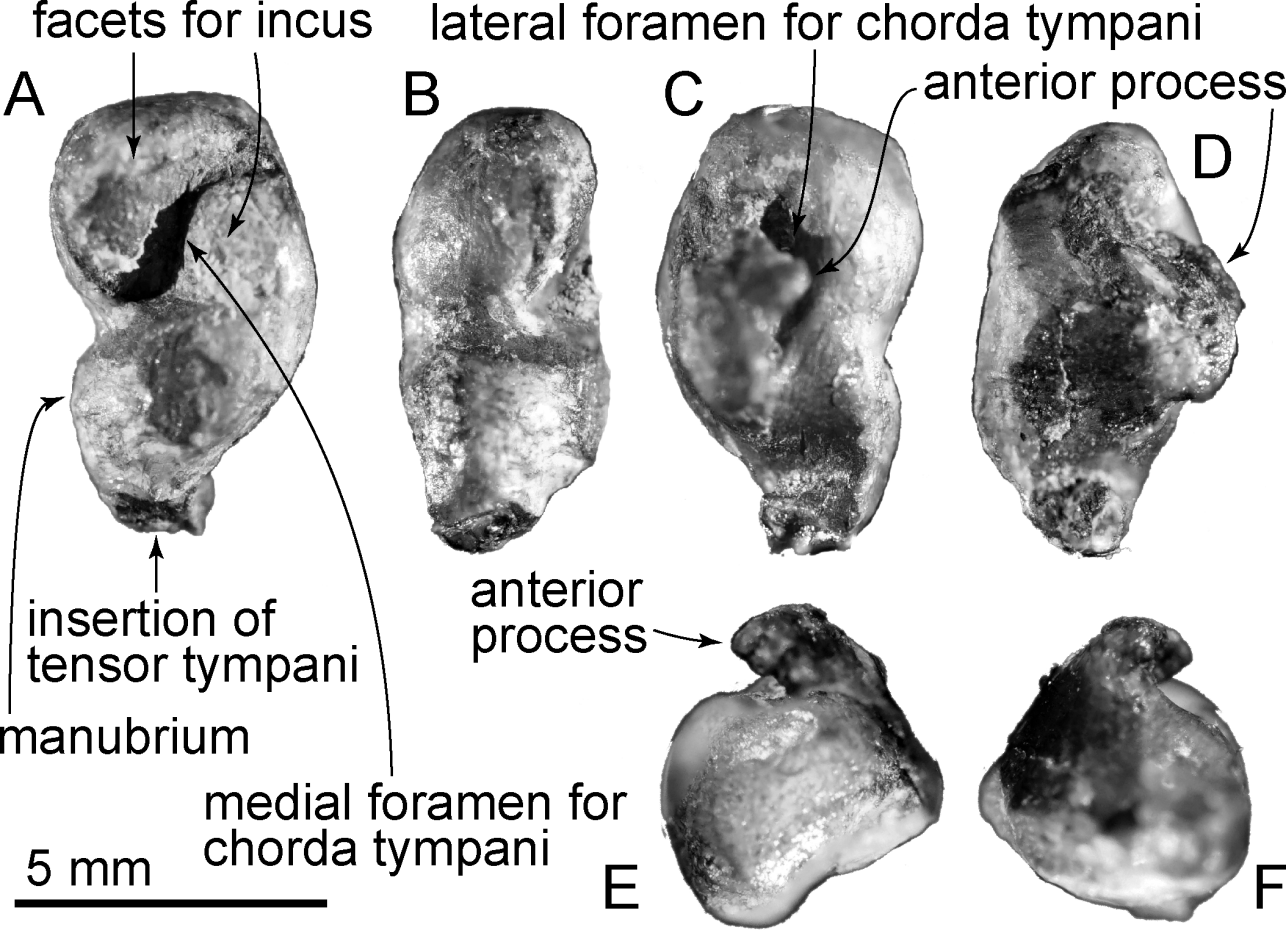
Right malleus of *Urkudelphis chawpipacha*, MO-1 (holotype). (A) Posteromedial view. (B) Posterior view. (C) Lateral view. (D) Anterior view. (E) Dorsal view. (F) Ventral view.

Medial to the sigmoid process of the tympanic bulla, a long *in situ* malleus was removed for study. Preservation is limited and the exact orientations are uncertain. The head has a flattened subspherical profile, and is little-elevated above the incudal facets. A weak constriction at the center separates the manubrium from the incudal facets (Figs 11–13). Ventrally, the blunt curved end on the manubrium forms the insertion of the tympanic ligament; the origin for the tensor tympani is not obvious on the opposite face. On the posteromedial surface (Fig 13A) an irregular depression between the incudal facets may be the medial foramen for chorda tympani or, alternatively, a preservational artifact. On the lateral face, the poorly preserved anterior process arises from the margin of a deep depression, which Kellogg ([37], 193) termed, in Archaeoceti, the “little pit for the chorda tympani.”

### Phylogenetic relationships

The phylogenetic position of MO-1 was analyzed using the matrix of Tanaka and Fordyce [38], which was originally derived from Tanaka and Fordyce [13], slightly modified as below. The expanded Tanaka & Fordyce [38] matrix includes 83 extant and extinct named and described taxa; one taxon, OU 22125, was recently named as *Awamokoa tokarahi*, while another, OU 22670, is not formally described. 284 characters (with 31 soft tissue characters) are cited and/or modified from previous studies [11, 39–55].

The percentage of missing data of MO-1 is 61% (including soft tissue characters) and 56% (excluding soft tissue characters). However, the earbones are well coded; the periotic shows 33 of 34 characters (97%) and the tympanic bulla 10 of 19 characters (53%).

**Fig 14:**
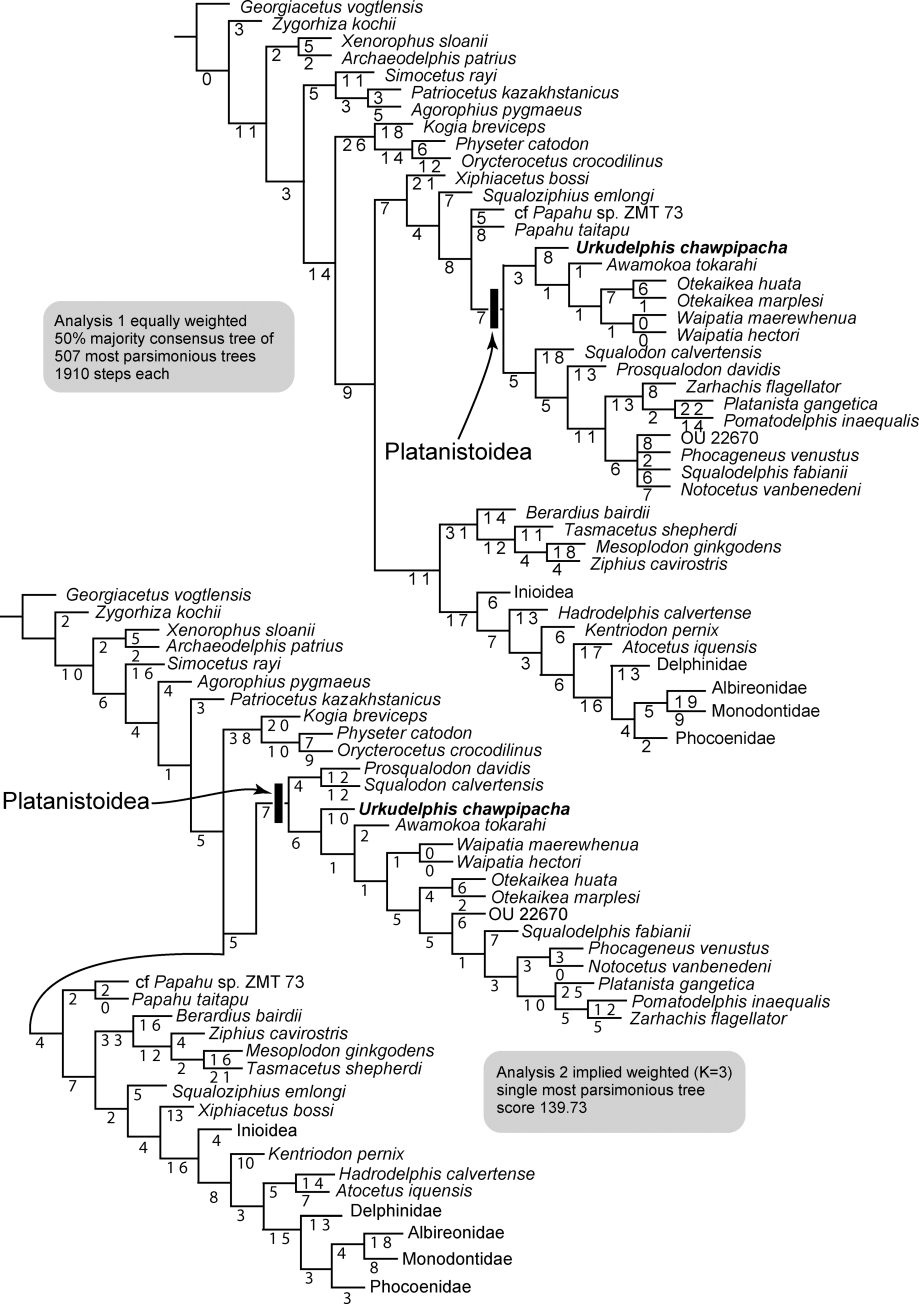
Phylogenetic analyses of *Urkudelphis chawpipacha*. The clades Inioidea, Phocoenidae and Delphinidae are collapsed. (Cladograms with all clades shown are in Supplementary Files, Figs 8 and 9.) Top, 50% majority consensus tree of equally weighted analysis 1 with branch length labeled. Bottom, single shortest tree of implied weighting analysis 2 with branch length labeled.

Data are provided as supporting information. S2 and S3 Files show the data matrix in nexus and TNT formats respectively; S4 File is the character list, and S5 File lists modified codings. S6 and S7 Files are tree files for analyses 1 and 2 respectively. S1 and S2 Figs show the full trees, which were used to produce the trees with collapsed clades of Fig 14.

Character data and tree data were managed using Mesquite version 2.75 [55]. Two different analyses were performed with TNT version 1.5 [56]. All characters were treated either as unweighted and unordered (analysis 1), or with implied weights [57] with K = 3 and unordered (analysis 2). The outgroup was the protocetid *Georgiacetus vogtlensis*. Both analyses used New Technology Search with the setting: recover minimum length trees = 1000 times with a backbone constraint of extant taxa, based on the topology of the McGowen *et al*. [58] molecular phylogeny. For ease of illustrating, species in some taxa among the Delphinida (Inioidea, Phocoenidae and Delphinidae) were collapsed after the analyses.

Both analyses 1 and 2 placed MO-1 in the Platanistoidea [11], and as basal to *Awamokoa tokarahi* (Fig 14). Analysis 1 shows a clade of *Waipatia* spp. + *Otekaikea* spp. + *A*. *tokarahi* and MO-1. Conversely, analysis 2 shows the latter species (*Waipatia* spp., *Otekaikea* spp., *A*. *tokarahi* and MO-1) not forming a clade.

#### Analysis 1; Unweighted and unordered

The phylogenetic analysis shows 507 shortest trees of 1910 steps each. The 50% majority rule consensus tree (Fig 14, top) shows the same topology as for the unweighted and unordered analysis tree of Tanaka & Fordyce [38].

#### Analysis 2; Implied weighting

The analysis recovers a single shortest tree with a score of 139.73. The single tree (Fig 14, bottom) shows the same topology as for the implied weighting analysis tree of Tanaka & Fordyce [38].

## Discussion

### Ontogeny and phylogenetics

Specimen MO-1 is interpreted as a juvenile. Studies on living cetacean species have shown marked ontogenetic change in some parts of the skull, particularly the feeding apparatus (rostrum, temporal fossa), e.g. [33, 59] One might expect comparable ontogenetic change in the size and shape of bones and the skull in fossil cetaceans, with implications for cladistic coding and phylogenetic placement. In living odontocetes, the periotic ossifies early in fetal development [60], and generally shows little change in size or shape after birth [61]. Further, this single element is feature-laden [21], with many characters available for cladistic analysis and/or identification to species level [61]. Here, the *Urkudelphis* periotic provides 34 characters, including apomorphies for major nodes in the Platanistoidea (Fig 14). Where possible, the periotic and/or tympanic bulla should be used particularly in phylogenetic analyses of suspected juvenile specimens.

### Comparison with other Oligocene dolphins

The phylogenetic analyses show that *Urkudelphis* is distinct from other named genera of odontocetes in skull and earbone features, some of which are noted below. *Urkudelphis* differs from Oligocene basal odontocetes (*Ashleycetus*, *Xenorophus*, *Agorophius*, *Squaloziphius*, *Simocetus* and *Mirocetus*), in the structure of the vertex: the large frontals have a nearly-square exposure on the vertex, associated with a parallel-sided ascending process of the maxilla, as in some more-crownward Late Oligocene and Neogene dolphins (e.g. especially *Waipatia*; see also *Otekaikea*, *Iniopsis* [62], and *Eosqualodon* [63]). But, the Late Oligocene *Patriocetus kazakhstanicus* [64] differs in having large frontals with an anteriorly narrower and posteriorly wider exposure on the vertex. MO-1 differs from other named Late Oligocene dolphins in its larger frontals at the vertex, and a dorsally wide open vomer forming a mesorostral groove reminiscent of *Ashleycetus* [65] and *Xenorophus* [9]. Also of note in *Urkudelphis* is the interparietal, which invades the posterior of the frontals at the vertex, and the palatine, which projects forward into the maxilla in the ventral midline. A comparable palatine condition occurs more strongly in *Simocetus*. *Urkudelphis* differs from Early Miocene *Papahu taitapu*, *Chilcacetus cavirhinus* [66], *Arktocara yakataga* [18], *Allodelphis pratti* [67, 68] and *Ninjadelphis ujiharai* [69], having the frontals on the vertex are flatter and longer than the taxa above, which have more nodular and shorter frontals. *Urkudelphis* differs from *Chilcacetus* and *Papahu* in having a narrow premaxillary sac fossa. The antorbital notch of *Urkudelphis* is very shallow, which is different from *Patriocetus*, *Xenorophus*, *Prosqualodon*, *Squaloziphius* [70], *Chilcacetus* and *Ninjadelphis*. *Urkudelphis* does not preserve the nasal, but the frontal shows shallow fossae probably for thin nasals. A comparable condition can be seen in *Chilcacetus*.

The *Urkudelphis* periotic is similar to that of the Late Oligocene *Waipatia maerewhenua*, *Otekaikea* spp. and *Awamokoa tokarahi*, in its slender and ventrally bent anterior process and anteroposteriorly long elliptical internal acoustic meatus. MO-1 differs from *Waipatia* and *Otekaikea* spp. in a weakly curved parabullary sulcus similar to that of *Awamokoa*. Among the Platanistoidea, the *Urkudelphis* periotic uniquely shows approximately the same size of the aperture for vestibular aqueduct and cochlear aqueduct (character 186), and can be diagnosed using a combination of two ear bone characters: a thin pars cochlearis of the periotic (character 192); and a posterior edge of the medial prominence of the involucrum located anterior to the posterior edge of the lateral prominence (character 218). The strong lateral tuberosity in MO-1 is even stronger than the prominent tuberosity in *Awamokoa* and *Allodelphis pratti*. *Allodelphis*, and the other Early Miocene Allodelphinidae (*Ninjadelphis* and *Arktocara*) differ, however, in many aspects of skull morphology; for example, the pterygoid sinus fossa is anteroposteriorly longer, and the posterior portion of the premaxillae dorsoventrally thin and narrow, separated by narrow nasals.

The tympanic bulla of MO-1 lacks a prominent anterior spine, the presence of which has been proposed as a synapomorphy of the Platanistoidea [71]. However, the spine has an uncertain ontogeny, and also may often be damaged; thus, its state is uncertain in early platanistoids. For example, *Waipatia maerewhenua* shows an incipient anterior spine, while *Awamokoa tokarahi* and *Otekaikea* spp. have a damaged bullar apex. Further, the tympanic bulla in some living delphinids has a small spine, which lengthens with ontogeny, although not to the length seen in Platanistidae [61].

The *Urkudelphis* malleus is similar to that of *Huaridelphis* in its slender manubrium, contrasting with the blunt manubrium in *Notocetus vanbenedeni* [71], and *Eurhinodelphis cocheteuxi* [48]. *Zarhachis* [72], *Phocageneus venustus* and *Pomatodelphis* cf. *inaequalis* [72] differ from *Urkudelphis* in their pointed manubrium. The *Urkudelphis* malleus has large facets for the incus compared with other dolphins (such as Delphinida in Muizon [72] and *Notocetus*, *Phocageneus* and *Pomatodelphis* in Muizon [71]), but compared to *Inticetus vertizi* [30], it is smaller.

### Paleoenvironment

During the Late Cretaceous to Eocene, the Ecuador region was tectonically active with substantial crustal translation and rotation. In the later Oligocene to Neogene, Andean tectonics included forearc subsidence and localised basin development, but no great latitudinal displacement of the Montañita/Olón fossil locality (e.g. [73]). Thus, *Urkudelphis* probably lived in a near-equatorial setting. Whether the habitat was estuarine or neritic (mid-shelf waters) is uncertain, so we cannot consider paleoecology; besides, the *Urkudelphis* skull is not preserved well enough to interpret functional complexes such as the feeding and hearing apparatus. During the late Oligocene, the Central American Seaway was open, probably with major equatorial currents ([74] Figs 7 and 10). Other tropical-latitude cetacean fossils have been reported recently from South America, including aforementioned Neogene material from Ecuador [19, 20], Chile [75] and Peru [76–78]. Mio-Pliocene cetaceans, including species of Mysticeti, Iniidae and Platanistidae, were reported recently from Colombia and Venezuela [79], adding to material reported from Costa Rica, Panama, and other South American localities noted by Aguirre-Fernández et al. [79].

## Conclusion

A new small dolphin from probable Oligocene (Chattian?) strata in Santa Elena, Ecuador is described as a new species and genus, *Urkudelphis chawpipacha*. The new taxon is characterized by: an anteromedially oriented anterior edge of the supraorbital process; weakly convex nuchal crest in dorsoposterior view; approximately same sized apertures of vestibular aqueduct and cochlear aqueduct; dorsoventrally thin pars cochlearis on periotic; and inner posterior prominence placed anterior to the outer posterior prominence. *Urkudelphis chawpipacha* differs from other Oligocene dolphins in the combination of: frontals on the vertex at a level posterior to the postorbital process; shorter and wider frontals; and parallel-sided posterior part of the ascending process of the maxilla. Phylogenetic analysis places it near the base of the largely-extinct clade Platanistoidea. The fossil is one of few fossil Neoceti reported from the equator, and is a reminder that Oligocene cetaceans may have ranged widely in tropical waters.

## Supporting information

S1 Fig Full 50% majority consensus tree of analysis 1. (TIF)

S2 Fig Full single tree of analysis 2. (TIF)

S1 File Body restoration formula from Pyenson and Sponberg (2011). (R)

S2 File Cladistic matrix in nex format. (NEX)

S3 File Cladistic matrix in tnt format. (TNT)

S4 File Character list. (DOCX)

S5 File List of modifications for character list. (DOCX)

S6 File Tree file of analysis 1. (TRE)

S7 File Tree file of analysis 2. (TRE)
