# Supplementary figures and images for: A new tropical Oligocene dolphin from Montañita/Olón, Santa Elena, Ecuador
Source: PLoS One. 2017 Dec 20;12(12):e0188380. doi: 10.1371/journal.pone.0188380 (PMC5737981; doi:10.1371/journal.pone.0188380)

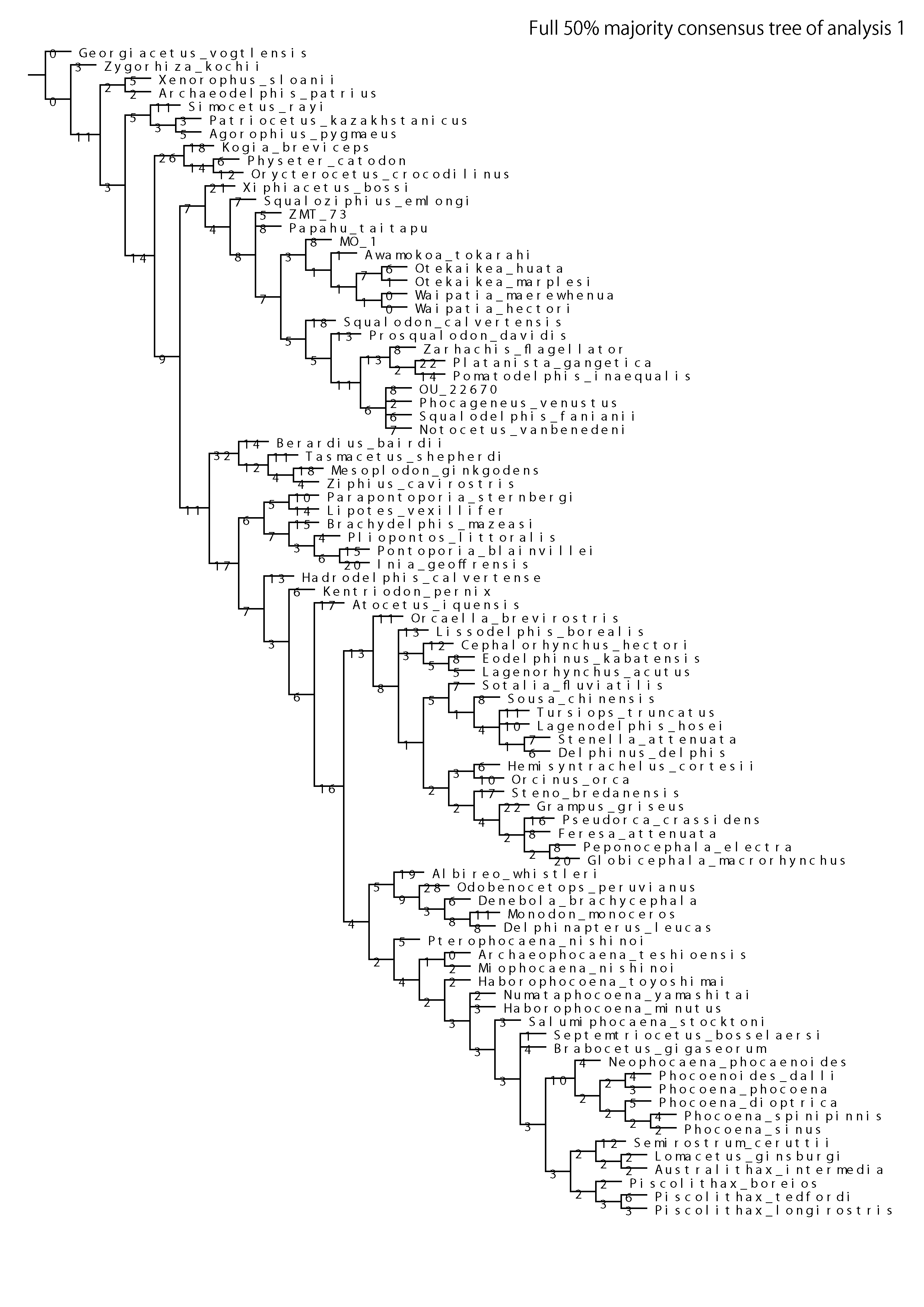

Supplement: S1 Fig — (TIF) [file pone.0188380.s001.tif]

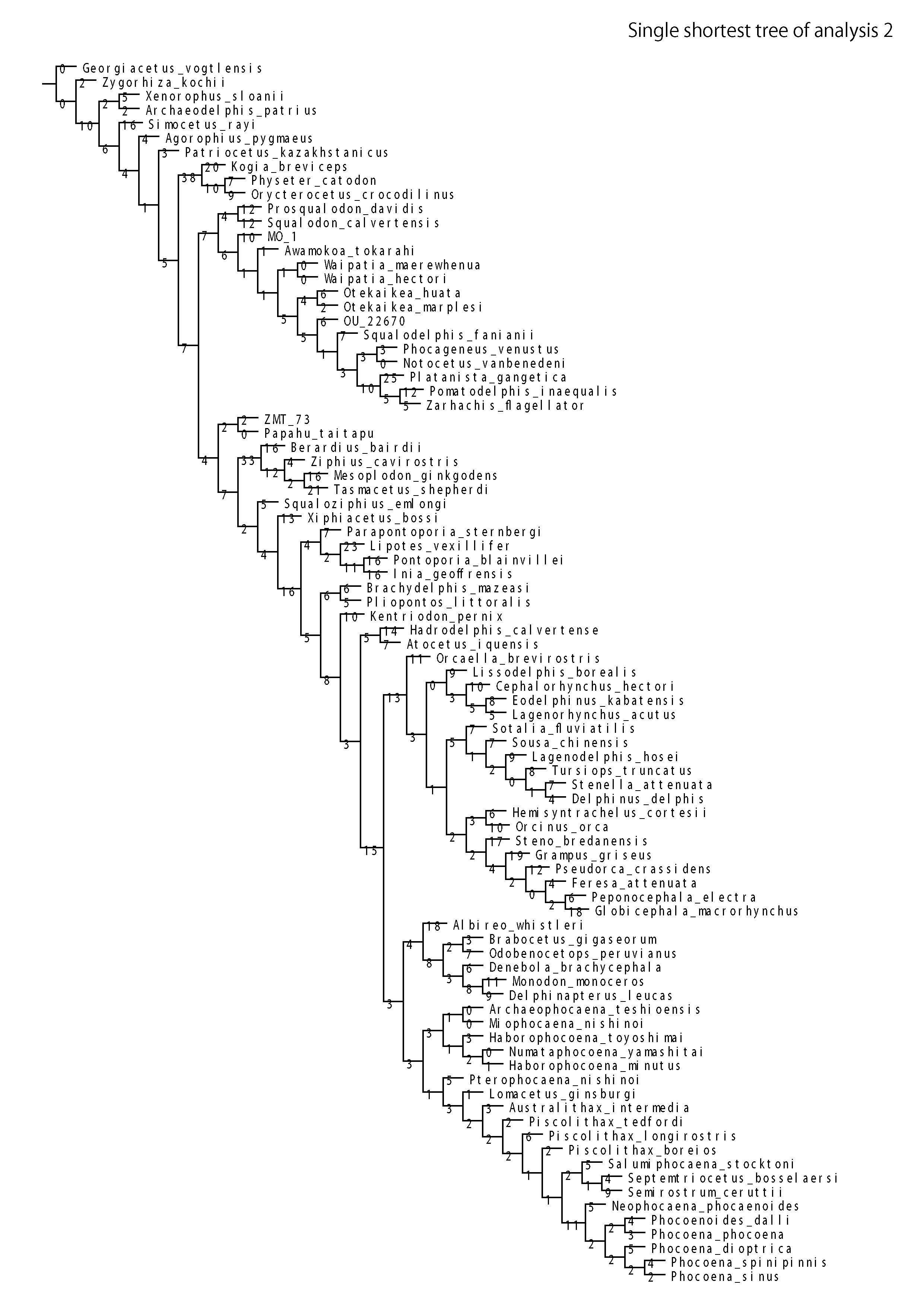

Supplement: S2 Fig — (TIF) [file pone.0188380.s002.tif]
