# Supplementary material for: A new tropical Oligocene dolphin from Montañita/Olón, Santa Elena, Ecuador
Source: PLoS One. 2017 Dec 20;12(12):e0188380. doi: 10.1371/journal.pone.0188380 (PMC5737981; doi:10.1371/journal.pone.0188380)
Supplement: S4 File — (DOCX) [file pone.0188380.s006.docx]

Appendix 4.

Morphological characters used in the phylogenetic analysis.

Terminology generally follows that of the cladistic papers cited, which in a few cases does not agree with the recommended uses of Mead and Fordyce ([2009](#_ENREF_52)). For each character, references are given for the main past uses, with the relevant published character number given with a hatch # thus: Murakami et al. ([2012a](#_ENREF_57)) #1.

**Rostrum, Dental, and Mandibular**

(1) Length of rostrum as percent skull length: moderately long, 50–55% (0); long, 55–60% (1); very long, >60% (2); medium, 50–40% (3); very short, 40–35% (4). (Murakami et al. ([2012a](#_ENREF_57), [2012b](#_ENREF_58)) #1; modified from Arnold and Heinsohn, ([1996](#_ENREF_3)) #8; Bianucci, ([2005](#_ENREF_9)) #1; Lambert, ([2008](#_ENREF_46)) #1; Tanaka and Fordyce, ([2014](#_ENREF_69), [2015](#_ENREF_70)) #1).

(2) Premaxillae transverse proportion: transversely inflated almost entire length of rostrum (0); flat almost entire length of the rostrum (1). (Murakami et al. ([2012a](#_ENREF_57), [2012b](#_ENREF_58)) #2; Tanaka and Fordyce, (2014, 2015) #2).

(3) Premaxillae mediolateral proportion: not compressed mediolaterally (0); compressed mediolaterally at anterior of rostrum (1). (Murakami et al. ([2012a](#_ENREF_57), [2012b](#_ENREF_58)) #3; Tanaka and Fordyce, (2014, 2015) #3).

(4) Premaxillae at apex of rostrum: with lateral margins parallel or diverging (0); narrowing (1). (Murakami et al. ([2012a](#_ENREF_57), [2012b](#_ENREF_58)) #4; modified from Bianucci, ([2005](#_ENREF_9)) #2; Tanaka and Fordyce, (2014, 2015) #4).

(5) Maxilla length as percent rostrum length: short, <85%, tips of maxillae not reaching tip of rostrum, (0); long, >89%, tips of maxillae to within 10% of rostrum tip (1); same as state 1 except lack of alveoli (2). (Murakami et al. ([2012a](#_ENREF_57), [2012b](#_ENREF_58)) #5; modified from Lambert, ([2005](#_ENREF_45)) #1; Tanaka and Fordyce, (2014, 2015) #5).

(6) Mesorostral groove: V-shaped or U-shaped opening (0); partially or completely filled in with vomer, becoming a solid rod of bone (1); absent (2). (Messenger and McGuire, ([1998](#_ENREF_54)) #1429; Geisler and Sanders, ([2003](#_ENREF_34))#5; Geisler et al., ([2011](#_ENREF_33)) ([2012](#_ENREF_31)) #5; Murakami et al. ([2012a](#_ENREF_57), [2012b](#_ENREF_58)) #6; derived from Moore, ([1968](#_ENREF_56)) ; Tanaka and Fordyce, (2014, 2015) #6).

(7) Mesorostral groove constricted posteriorly, anterior to the nares and behind the level of the antorbital notch, then rapidly diverging anteriorly: absent (0); present (1). (modified from Murakami et al. ([2012b](#_ENREF_58)) #279; Tanaka and Fordyce, (2014, 2015) #7).

(8) Lateral margin of rostrum anterior to maxillary flange: concave (0); straight (1); convex (2); absent (3) (Murakami et al. ([2012a](#_ENREF_57), [2012b](#_ENREF_58)) #7; modified from Bianucci, ([2005](#_ENREF_9)) #3; Tanaka and Fordyce, (2014, 2015) #8).

(9) Rostral constriction: absent (0); constriction anterior to antorbital notch (1); constriction anterior to maxillary flange (2). (Murakami et al. ([2012a](#_ENREF_57), [2012b](#_ENREF_58)) #8; modified from Muizon, ([1984](#_ENREF_14)); Barnes, ([1985b](#_ENREF_7)); Messenger and McGuire, ([1998](#_ENREF_54)) #1424; Geisler and Sanders, ([2003](#_ENREF_34)) #6; Geisler et al., ([2012](#_ENREF_31); [2011](#_ENREF_33)) #6; Tanaka and Fordyce, (2014, 2015) #9).

(10) Antorbital notch: absent or weakly developed (0); well developed (1). (Messenger and McGuire, ([1998](#_ENREF_54)) #1426; Fajardo-Mellor et al., (2006) #6; Murakami et al. ([2012a](#_ENREF_57), [2012b](#_ENREF_58)) #9; Tanaka and Fordyce, (2014, 2015) #10).

(11) Width of premaxillae at mid-rostrum as percent greatest width of maxillae at level of postorbital processes: wide, >25% (0); medium, 25–15% (1); narrow, <15% (2). (Murakami et al. ([2012a](#_ENREF_57), [2012b](#_ENREF_58)) #10; modified from Aguirre-Fernandez et al., ([2009](#_ENREF_1)) #4) ; Tanaka and Fordyce, (2014, 2015) #11)

(12) Width of rostrum at mid-length as percent greatest width of maxillae at level of postorbital processes: wide, >35% (0); medium, 35–30% (1); narrow, <30% (2). (Murakami et al. ([2012a](#_ENREF_57), [2012b](#_ENREF_58)) #11; modified from Aguirre-Fernandez et al., ([2009](#_ENREF_1)) #6; Tanaka and Fordyce, (2014, 2015) #12).

(13) Width of rostrum at antorbital notch as percent greatest width of maxillae at level of postorbital processes: wide, >68% (0); medium, 68–45% (1); narrow, <45% (2). (Murakami et al. ([2012a](#_ENREF_57), [2012b](#_ENREF_58)) #12; modified from Geisler and Sanders, ([2003](#_ENREF_34)) #7; Geisler et al., ([2012](#_ENREF_31); [2011](#_ENREF_33)) #7; Tanaka and Fordyce, (2014, 2015) #13).

(14) Premaxillae in dorsal view: contacting along midline for less than half length of rostrum (0); widely separated by mesorostral groove in rostrum (1); narrowly separated by mesorostral groove in rostrum (2); contacting along midline for approximately half the entire length or more than of rostrum but not fused (3); contacting along midline for approximately half the entire length or more than of rostrum and partially fused (4); converging (either contacting and separating) in mid-rostrum (5). (modified from Murakami et al. ([2012a](#_ENREF_57), [2012b](#_ENREF_58)) #13; modified from Muizon, ([1988](#_ENREF_17)); Fordyce, ([1994](#_ENREF_28)) #52; Messenger and McGuire, ([1998](#_ENREF_54)) #1405; Geisler and Sanders, ([2003](#_ENREF_34)) (9); Geisler et al., ([2011](#_ENREF_33)) ([2012](#_ENREF_31)) #9; Tanaka and Fordyce, (2014, 2015) #14).

(15) Suture between maxilla and premaxilla on rostrum: unfused except distal tip of rostrum (0); fused partly or along most of rostrum (1). (Murakami et al. ([2012a](#_ENREF_57), [2012b](#_ENREF_58)) #14; modified from Fordyce, ([1994](#_ENREF_28)) #36; Messenger and McGuire #1418, ([1998](#_ENREF_54)); Geisler and Sanders, ([2003](#_ENREF_34)) #10; Lambert, ([2005](#_ENREF_45)) #2; Geisler et al., ([2011](#_ENREF_33)) ([2012](#_ENREF_31)) #10; Tanaka and Fordyce, (2014, 2015) #15).

(16) Posterior wall of antorbital notch: maxilla (0); lacrimal and jugal, or maxilla appeared in small area posterior to antorbital notch parallel with lacrimal and jugal (1); no notch but horizontal groove inferred to be for the facial nerve in the maxilla laterally on the face well above the margin of the rostrum (2). (Murakami et al. ([2012a](#_ENREF_57), [2012b](#_ENREF_58)) #15; modified from Geisler and Sanders, ([2003](#_ENREF_34)) (15); Geisler et al., ([2011](#_ENREF_33)) ([2012](#_ENREF_31)) #15; Tanaka and Fordyce, (2014, 2015) #16).

(17) Anterior sinus fossa: absent (0); between anterior extremity of pterygoid sinus and posterior extremity of upper tooth row (1); between posterior extremity of upper tooth row and midpoint of rostrum (2); beyond midpoint of rostrum (3). (Murakami et al. ([2012a](#_ENREF_57), [2012b](#_ENREF_58)) #17; modified from Muizon, ([1988](#_ENREF_17)); Barnes, ([1990](#_ENREF_8)); Bianucci, ([2005](#_ENREF_9)) #13; Arnold and Heinsohn, ([1996](#_ENREF_3)) #21; Geisler and Sanders, ([2003](#_ENREF_34)) #157; Aguirre-Fernandez et al., ([2009](#_ENREF_1)) #18; Geisler et al., ([2011](#_ENREF_33)) ([2012](#_ENREF_31)) #157; derived from Fraser and Purves, ([1960](#_ENREF_30)) ; Tanaka and Fordyce, (2014, 2015) #18).

**Teeth**

(18) Number of double-rooted teeth in maxilla: 6–8 (0); 0 (1). (modified from Geisler and Sanders, ([2003](#_ENREF_34)) #23; Geisler et al., ([2011](#_ENREF_33)) ([2012](#_ENREF_31)) #23; Murakami et al. ([2012a](#_ENREF_57), [2012b](#_ENREF_58)) #18; Tanaka and Fordyce, (2014, 2015) #19).

(19) Tooth enamel: reticulating striae (0); smooth (1); nodular (2); absent (3). (Murakami et al. ([2012a](#_ENREF_57), [2012b](#_ENREF_58)) #20; modified from Messenger and McGuire, ([1998](#_ENREF_54)) #1469; Geisler and Sanders, ([2003](#_ENREF_34)) #26; Geisler et al., ([2012](#_ENREF_31); [2011](#_ENREF_33)) #26; derived from Zhou, ([1982](#_ENREF_76)) ; Tanaka and Fordyce, (2014, 2015) #20).

(20) Teeth: heterodont and some teeth with denticle (0); conical, with or without accessory cusp (1); spatulate (2); laterally compressed (3). (Murakami et al. ([2012a](#_ENREF_57), [2012b](#_ENREF_58)) #21; modified from Heyning, ([1989](#_ENREF_36)) #40, ([1997](#_ENREF_37)) #72; Arnold and Heinsohn,([1996](#_ENREF_3)) #25; Messenger and McGuire, ([1998](#_ENREF_54)) #1470; Geisler and Sanders, ([2003](#_ENREF_34)) #27, ([2012](#_ENREF_31)) #27; Lambert, ([2008](#_ENREF_46)) #16; Geisler et al., ([2011](#_ENREF_33)) #27; derived from Barnes, ([1984a](#_ENREF_4)) ; Tanaka and Fordyce, (2014, 2015) #21).

(21) Upper anterior teeth: about same size as upper posterior teeth (0); greatly enlarged (1); clearly smaller than upper posterior teeth or absent (2). (modified from Murakami et al. ([2012a](#_ENREF_57), [2012b](#_ENREF_58)) #22; Tanaka and Fordyce, (2014, 2015) #22).

(22) Incisors relatively delicate and procumbent: no (0); yes (1). (Fordyce ([1994](#_ENREF_28)) #42;Tanaka and Fordyce, (2014, 2015) #285)

(23) Crown of heterodont teeth: long (>10 mm) (0); short (<10mm) (1). (Fordyce ([1994](#_ENREF_28)) #58; Tanaka and Fordyce, (2014, 2015) #286)

(24) Large incisor tusk: absent (0); present (1). (Tanaka and Fordyce, (2014, 2015) #287)

(25) Cheek teeth ectocingulum: present (0); absent (1). (Murakami et al. ([2012a](#_ENREF_57), [2012b](#_ENREF_58)) #23; modified from Geisler and Sanders, ([2003](#_ENREF_34)) #31; Geisler et al., ([2012](#_ENREF_31); [2011](#_ENREF_33)) #31; Tanaka and Fordyce, (2014, 2015) #23).

(26) Cheek teeth entocingulum: present (0); absent (1). (Geisler and Sanders, ([2003](#_ENREF_34)) #32; Geisler et al., ([2012](#_ENREF_31); [2011](#_ENREF_33)) #32; Murakami et al. ([2012a](#_ENREF_57), [2012b](#_ENREF_58)) #24; Tanaka and Fordyce, (2014, 2015) #24).

(27) Greatest diameter of largest functional tooth as percent of greatest width of maxillae at the level of the postorbital processes: large, >5% (0); medium, 5–3% (1); small, <3% (2). (Murakami et al. ([2012a](#_ENREF_57), [2012b](#_ENREF_58)) #25; modified from Aguirre-Fernandez et al., ([2009](#_ENREF_1)) #15; Tanaka and Fordyce, (2014, 2015) #25).

**Mandible**

(28) Anterior mandibular teeth: deeply rooted, root >50% of tooth (0); not deeply rooted, root <50% of tooth (1). (Messenger and McGuire, ([1998](#_ENREF_54)) #1471; Geisler and Sanders, ([2003](#_ENREF_34)) #28; Geisler et al., ([2012](#_ENREF_31); [2011](#_ENREF_33)) #28; Murakami et al. ([2012a](#_ENREF_57), [2012b](#_ENREF_58)) #26; derived from Flower, ([1872](#_ENREF_25)); Moore, ([1968](#_ENREF_56)) ; Tanaka and Fordyce, (2014, 2015) #26).

(29) Anterior-most mandibular "tooth": about same size as posterior teeth (0); smaller than posterior teeth (1); greatly enlarged (2); forming a tusk (3). (Murakami et al. ([2012a](#_ENREF_57), [2012b](#_ENREF_58)) #27; modified from Muizon, ([1991](#_ENREF_18)); Geisler and Sanders, ([2003](#_ENREF_34)) #36; Messenger and McGuire, ([1998](#_ENREF_54)) #1477; Geisler et al., ([2012](#_ENREF_31); [2011](#_ENREF_33)) #36; derived from Flower, ([1872](#_ENREF_25)) ; Tanaka and Fordyce, (2014, 2015) #27).

(30) Number of teeth in mandible: 16–11 (0); 9–8 (1); 2 (2); 1 (3); 17–23 (4); 24–27 (5); 28–39 (6); >40 (7). (Murakami et al. ([2012a](#_ENREF_57)) #28; modified from Messenger and McGuire, ([1998](#_ENREF_54)) #1468; Geisler and Sanders, ([2003](#_ENREF_34)) #37; Geisler et al., ([2012](#_ENREF_31); [2011](#_ENREF_33)) #37; Tanaka and Fordyce, (2014, 2015) #28).

(31) Length of mandibular symphysis as percent of mandible length: long, >20% (0); short, <20% (1). (Murakami et al. ([2012a](#_ENREF_57), [2012b](#_ENREF_58)) #29; modified from Messenger and McGuire, ([1998](#_ENREF_54)) #1465; Arnold and Heinsohn,([1996](#_ENREF_3)) #7; Bianucci, ([2005](#_ENREF_9)) #26; Tanaka and Fordyce, (2014, 2015) #29).

(32) Mandibular symphysis: sutured but unfused (0); fused (1). (Fordyce, ([1994](#_ENREF_28)) #5; Messenger and McGuire, ([1998](#_ENREF_54)) #1466; Geisler and Sanders, ([2003](#_ENREF_34)) #40; Geisler et al., ([2011](#_ENREF_33)) ([2012](#_ENREF_31)) #40; Murakami et al. ([2012a](#_ENREF_57), [2012b](#_ENREF_58)) #30; derived from Flower, ([1885](#_ENREF_27)) ; Tanaka and Fordyce, (2014, 2015) #30).

(33) Longitudinal groove on underside of mandibles: absent (0); present (1). (Geisler and Sanders, ([2003](#_ENREF_34)) #41; Geisler et al., ([2012](#_ENREF_31); [2011](#_ENREF_33)) #41; Murakami et al. ([2012a](#_ENREF_57), [2012b](#_ENREF_58)) #31; derived from Miller, ([1923](#_ENREF_55)) ; Tanaka and Fordyce, (2014, 2015) #31).

(34) Mandible: bowed medially (0); straight (1); slightly bowed laterally (2). (Sanders and Barnes, 2002; Geisler and Sanders, ([2003](#_ENREF_34)) #42; Geisler et al., ([2011](#_ENREF_33)) ([2012](#_ENREF_31)) #42; Murakami et al. ([2012a](#_ENREF_57), [2012b](#_ENREF_58)) #32; derived from Miller, ([1923](#_ENREF_55)) ; Tanaka and Fordyce, (2014, 2015) #32).

(35) Elevation of coronoid process: very high (0); moderate (1); low (2). (Murakami et al. ([2012a](#_ENREF_57), [2012b](#_ENREF_58)) #33; modified from Geisler and Sanders, ([2003](#_ENREF_34)) #44; Bianucci, ([2005](#_ENREF_9)) #27; Geisler et al, ([2012](#_ENREF_31); [2011](#_ENREF_33)) #44; Tanaka and Fordyce, (2014, 2015) #33).

**Orbit**

(36) Antorbital process of maxilla in dorsal view: triangular (0); robust and globose or rectilinear (1); absent (2). (Bianucci, ([2005](#_ENREF_9)) #4; Murakami et al. ([2012a](#_ENREF_57), [2012b](#_ENREF_58)) #34; Tanaka and Fordyce, (2014, 2015) #34).

(37) Angle of anterior edge of supraorbital process and the median line: oriented slightly anterolaterally, at an angle <30° (±) with sagittal plane (0) oriented anteromedially (1). (Murakami et al. ([2012a](#_ENREF_57), [2012b](#_ENREF_58)) #35; modified from Geisler and Sanders, ([2003](#_ENREF_34)) #49; Geisler et al., ([2011](#_ENREF_33)) ([2012](#_ENREF_31)) #49; Tanaka and Fordyce, (2014, 2015) #35).

(38) Ratio of length of antorbital process of lacrimal to length of the orbit: <0.6 (0); ≥0.6 (1). (Murakami et al. ([2012a](#_ENREF_57), [2012b](#_ENREF_58)) #36; Tanaka and Fordyce, (2014, 2015) #36).

(39) Lacrimal: wrapping around anterior edge of supraorbital process of frontal and slightly overlying its anterior end (0); appearing dorsally and forming most of antorbital process (1); appearing dorsally but not prominently in antorbital process (2); restricted to below the supraorbital process of maxilla (3). (Murakami et al. ([2012a](#_ENREF_57), [2012b](#_ENREF_58)) #37; modified from Geisler and Sanders, ([2003](#_ENREF_34)) #51; Geisler et al., ([2012](#_ENREF_31); [2011](#_ENREF_33)) #51; derived from Kellogg, ([1923](#_ENREF_41)); Miller, ([1923](#_ENREF_55)) ; Tanaka and Fordyce, (2014, 2015) #37).

(40) Lacrimal foramen or groove: present (0); absent (1). (Geisler and Sanders, ([2003](#_ENREF_34)) #52; Geisler et al., ([2012](#_ENREF_31); [2011](#_ENREF_33)) #52; Murakami et al. ([2012a](#_ENREF_57), [2012b](#_ENREF_58)) #38; Tanaka and Fordyce, (2014, 2015) #38).

(41) Lacrimal and jugal fusion: separated (0); fused (1). (Heyning, ([1989](#_ENREF_36)) #7, ([1997](#_ENREF_37)) #39; Geisler and Sanders, ([2003](#_ENREF_34)) #53; Geisler et al., ([2012](#_ENREF_31); [2011](#_ENREF_33)) #53; Murakami et al. ([2012a](#_ENREF_57), [2012b](#_ENREF_58)) #39; derived from Flower, ([1868](#_ENREF_24)); Schulte, ([1917](#_ENREF_68)); Miller, ([1923](#_ENREF_55)) ; Tanaka and Fordyce, (2014, 2015) #39).

(42) Lacrimal and jugal contact: contacting each other externally (0); lacrimal excluded from edge of skull, jugal directly contacting anterior edge of frontal (1). (Geisler and Sanders, ([2003](#_ENREF_34)) #54; Geisler et al., ([2012](#_ENREF_31); [2011](#_ENREF_33)) #54; Murakami et al. ([2012a](#_ENREF_57), [2012b](#_ENREF_58)) #40; derived from Miller, ([1923](#_ENREF_55)) ; Tanaka and Fordyce, (2014, 2015) #40).

(43) Jugal: thick and sturdy (0); thin, splint, or absent (1). (Geisler and Sanders, ([2003](#_ENREF_34)) #56; Lambert, ([2005](#_ENREF_45)) #21; Geisler et al., ([2012](#_ENREF_31); [2011](#_ENREF_33)) #56; Murakami et al. ([2012a](#_ENREF_57), [2012b](#_ENREF_58)) #41; derived from Miller, ([1923](#_ENREF_55)); Barnes, ([1990](#_ENREF_8)) ; Tanaka and Fordyce, (2014, 2015) #41).

(44) Combined anteroposterior length of the lacrimal and jugal exposure that is posterior to antorbital notch: with skull in ventral view, exposure is small and combined length forms <50% of anteroposterior distance from antorbital notch to postorbital ridge (0); intermediate, forms between 50 and 62% of that distance (1); large, forms between 62 and 69% that distance (2); very large, forms >69% of that distance (3). (Murakami et al. in ([2012a](#_ENREF_57), [2012b](#_ENREF_58)) #42; modified from Geisler and Sanders, ([2003](#_ENREF_34)) #55; Geisler et al., ([2012](#_ENREF_31); [2011](#_ENREF_33)) #55; Tanaka and Fordyce, (2014, 2015) #42).

(45) Dorsolateral edge of internal opening of infraorbital foramen: formed by maxilla (0); formed by maxilla and lacrimal and/or jugal (l); formed by lacrimal and/or jugal (2); formed by frontal (3). (Geisler and Sanders, ([2003](#_ENREF_34)) #57; Geisler et al., ([2011](#_ENREF_33)) ([2012](#_ENREF_31)) #57; Murakami et al. ([2012a](#_ENREF_57), [2012b](#_ENREF_58)) #43; derived from Miller, ([1923](#_ENREF_55)) ; Tanaka and Fordyce, (2014, 2015) #43).

(46) Ventromedial edge of internal opening of infraorbital foramen: formed by maxilla (0); formed by maxilla and palatine and/or pterygoid (1); formed by palatine and/or pterygoid (2). (Geisler and Sanders, ([2003](#_ENREF_34)) #58; Geisler et al., ([2012](#_ENREF_31); [2011](#_ENREF_33)) #58; Murakami et al. ([2012a](#_ENREF_57), [2012b](#_ENREF_58)) #44; derived from Miller, ([1923](#_ENREF_55)) ; Tanaka and Fordyce, (2014, 2015) #44).

(47) Maxillary tuberosity: present (0); absent (1). (Geisler and Sanders, ([2003](#_ENREF_34)) #59; Geisler et al., ([2011](#_ENREF_33)) #59, ([2012](#_ENREF_31)) #59; modified from Murakami et al. ([2012a](#_ENREF_57)) #45; derived from Miller, ([1923](#_ENREF_55)) ; Tanaka and Fordyce, (2014, 2015) #45).

(48) Direction of apex of postorbital process of frontal: projected posterolaterally and slightly ventrally (0); directed ventrally (1); not clear because of extremely reduced process (2). (modified from Murakami et al. ([2012a](#_ENREF_57), [2012b](#_ENREF_58)) #46; Geisler and Sanders, ([2003](#_ENREF_34)) #61; Geisler et al., ([2012](#_ENREF_31); [2011](#_ENREF_33)) #61; Tanaka and Fordyce, (2014, 2015) #46).

(49) Shape of postorbital process of frontal: robust, blunt descending posteriorly (0); pointed, attenuated, or acute triangular (1); triangular, trapezoidal, or an anteroposteriorly widened falciform (2); dorsoventrally long falciform (3). (modified from Murakami et al. ([2012a](#_ENREF_57), [2012b](#_ENREF_58)) #47; Tanaka and Fordyce, (2014, 2015) #47).

(50) Frontal**-**maxilla suture angled posterodorsally at an angle of 50–70° (±) from axis of rostrum, with lateral exposure of frontal thickening posteriorly: absent (0); present (1). (Geisler and Sanders, ([2003](#_ENREF_34)) #48; Geisler et al., ([2011](#_ENREF_33)) #48, ([2012](#_ENREF_31)) #48; Murakami et al. ([2012a](#_ENREF_57), [2012b](#_ENREF_58)) #48; derived from Miller, ([1923](#_ENREF_55)) ; Tanaka and Fordyce, (2014, 2015) #48).

**Facial Region**

(51) Anterior dorsal infraorbital foramina: one (0); two (1); three or more (2). (Murakami et al. ([2012a](#_ENREF_57), [2012b](#_ENREF_58)) #49; modified from Barnes, ([1984b](#_ENREF_5)); Geisler and Sanders, ([2003](#_ENREF_34)) #64; Geisler et al., ([2011](#_ENREF_33)) #64, ([2012](#_ENREF_31)) #64; Tanaka and Fordyce, (2014, 2015) #49).

(52) Rostral basin: absent or poorly defined (0); present, situated medial to antorbital notch and anterior to supraorbital process of frontal (1). (Geisler and Sanders, ([2003](#_ENREF_34)) #65; Geisler et al., ([2011](#_ENREF_33)) #65, ([2012](#_ENREF_31)) #65; Murakami et al. ([2012a](#_ENREF_57), [2012b](#_ENREF_58)) #50; Tanaka and Fordyce, (2014, 2015) #50).

(53) Width of premaxillae at antorbital notches as percent width of rostrum at antorbital notch: narrow, <49% (0); moderate, 50–64% (1); wide, >65% (2); antorbital notch absent (3). (Geisler and Sanders, ([2003](#_ENREF_34)) #66; Geisler et al., ([2011](#_ENREF_33))#66, ([2012](#_ENREF_31)) #66; modified from Murakami et al. ([2012a](#_ENREF_57), [2012b](#_ENREF_58)) #51; Tanaka and Fordyce, (2014, 2015) #51).

(54) Size of premaxillary foramen: right and left subequal (0); right much larger than left (1); premaxillary foramen absent (2). (Messenger and McGuire, ([1998](#_ENREF_54)) #1415; Murakami et al. ([2012a](#_ENREF_57), [2012b](#_ENREF_58)) #53; modified from Geisler and Sanders, ([2003](#_ENREF_34)) #70; Geisler et al., ([2011](#_ENREF_33)) #70, ([2012](#_ENREF_31)) #70; Tanaka and Fordyce, (2014, 2015) #53).

(55) Position of premaxillary foramen: anterior of antorbital notch and anterior edge of supraorbital process (0); approximately medial to or posterior to antorbital notch region (1); premaxillary foramen absent (2). (Geisler and Sanders, ([2003](#_ENREF_34)) #71; Geisler et al., ([2011](#_ENREF_33)) #71, ([2012](#_ENREF_31)) #71; Murakami et al. ([2012a](#_ENREF_57), [2012b](#_ENREF_58)) #54; Tanaka and Fordyce, (2014, 2015) #54).

(56) Premaxillary foramen locating: medial (0); midpoint to lateral (1) absent (2). (modified from Murakami et al. ([Murakami et al., 2014](#_ENREF_59)): Murakami et al. ([2012b](#_ENREF_58)) #280; Tanaka and Fordyce, (2014, 2015) #55).

(57) Lateral margin of the right premaxilla posterior to premaxillary foramen: widen posteriorly (0); straight (1). (Murakami et al. ([2012b](#_ENREF_58)) #281; Tanaka and Fordyce, (2014, 2015) #56).

(58) Posterolateral sulcus: deep (0); shallow or absent (1); presence of additional posterolateral sulcus (longitudinal striation) (2). (Murakami et al. ([2012a](#_ENREF_57), [2012b](#_ENREF_58)) #55; modified from Muizon, ([1984](#_ENREF_14), [1988](#_ENREF_17)); Lambert, ([2008](#_ENREF_46)) #6; Geisler and Sanders, ([2003](#_ENREF_34)) #72; Geisler et al. ([2011](#_ENREF_33)) ([2012](#_ENREF_31)) #72; Tanaka and Fordyce, (2014, 2015) #57).

(59) Posterior projections of premaxillae: both premaxillae extending posterior to anterior tip of nasals (0); both premaxillae extending posterior to nasals (1); only right premaxillae extended posterior to nasal (2); neither premaxillae extending posterior to external nares, and narrow posterior end of premaxillae adjacent to external nares (3); neither premaxillae extending beyond external nares, and premaxillae displaced laterally by medial projection of maxilla (4); only right premaxilla extending beyond or in line with anterior-most portion of nasals (5). (Murakami et al. ([2012a](#_ENREF_57), [2012b](#_ENREF_58)) #76; modified from Muizon ([1984](#_ENREF_14)); Barnes, ([1985a](#_ENREF_6)); Heyning, ([1989](#_ENREF_36)) #39, 42, ([1997](#_ENREF_37)) #63, 71, 74; Arnold and Heinsohn,([1996](#_ENREF_3)) #35; Messenger and McGuire, ([1998](#_ENREF_54)) #1407, 1408; Fajardo-Mellor et al., (2006) #3; Lambert, ([2008](#_ENREF_46)) #5; Fordyce, ([1994](#_ENREF_28)) #27; Tanaka and Fordyce, (2014, 2015) #58).

(60) A posterior dorsal infraorbital foramen placed posteromedially, near posterior extremity of premaxilla: absent (0); present (1). (Fordyce, ([1994](#_ENREF_28)) #62; Lambert, ([2005](#_ENREF_45)) #13; Murakami et al. ([2012a](#_ENREF_57), [2012b](#_ENREF_58)) #58; Tanaka and Fordyce, (2014, 2015) #60).

(61) Premaxillary sac fossae: absent (0); present (1). (Messenger and McGuire, ([1998](#_ENREF_54)) #1411; Lambert, ([2005](#_ENREF_45)) #4; Murakami et al. ([2012a](#_ENREF_57), [2012b](#_ENREF_58)) #59; Tanaka and Fordyce, (2014, 2015) #61).

(62) Maxilla on dorsal surface of skull: does not contact supraoccipital posteriorly, maxilla separated by frontal and/or parietal (0); contact present (1). (Geisler and Sanders, ([2003](#_ENREF_34)) #129; Geisler et al., ([2011](#_ENREF_33)) #129, ([2012](#_ENREF_31)) #129, modified from Muizon, ([1991](#_ENREF_18)) ([1994](#_ENREF_19)); Murakami et al., ([2012a](#_ENREF_57), [2012b](#_ENREF_58)) #60; Tanaka and Fordyce, (2014, 2015) #62).

(63) Maxillae at anterior edge of supraorbital processes: abutting anterior edge of supraorbital processes of frontals (0); covering partially or almost completely surface of supraorbital processes (1). (Murakami et al. ([2012a](#_ENREF_57), [2012b](#_ENREF_58)) #61; modified from Fordyce, ([1994](#_ENREF_28)) #3; Messenger and McGuire, ([1998](#_ENREF_54)) #1419; Geisler and Sanders, ([2003](#_ENREF_34)) #76; Geisler et al., ([2011](#_ENREF_33)) #76, ([2012](#_ENREF_31)) #76; derived from Miller, ([1923](#_ENREF_55)) ; Tanaka and Fordyce, (2014, 2015) #63).

(64) Anterolateral corner of maxilla overlying supraorbital process of frontal: thin and equal in thickness to parts posteromedial (0); thickened with thinner maxilla in posteromedial direction (1). (Geisler and Sanders, ([2003](#_ENREF_34)) #78; Geisler et al., ([2011](#_ENREF_33)) #78, ([2012](#_ENREF_31)) #78; Murakami et al. ([2012a](#_ENREF_57), [2012b](#_ENREF_58)) #62; Tanaka and Fordyce, (2014, 2015) #64).

(65) Pneumatic maxillary crest overhanging medially: absent (0); present (1). (Zhou, ([1982](#_ENREF_76)); Heyning, ([1989](#_ENREF_36)) #26, ([1997](#_ENREF_37)) #58; Fordyce, ([1994](#_ENREF_28)) #66; Arnold and Heinsohn,([1996](#_ENREF_3)) #14; Messenger and McGuire, ([1998](#_ENREF_54)) #1421; Murakami et al. ([2012a](#_ENREF_57), [2012b](#_ENREF_58)) #63; Tanaka and Fordyce, (2014, 2015) #65).

(66) Maxillary crest on supraorbital process of maxilla: longitudinal ridges absent except at lateral edge of antorbital process (0); presence of longitudinal ridge except at lateral edge of antorbital process (1); longitudinal ridge present and joined with maxillary flange (2); presence of transversely compressed high crest, except at lateral edge of antorbital process (3); absent (4). (Murakami et al. ([2012a](#_ENREF_57), [2012b](#_ENREF_58)) #64; modified from Muizon, ([1984](#_ENREF_14)) ([1987](#_ENREF_16)); Barnes, ([1985b](#_ENREF_7)); Messenger and McGuire, ([1998](#_ENREF_54)) #1420; Geisler and Sanders, ([2003](#_ENREF_34)) #79; Geisler et al., ([2011](#_ENREF_33)) #79, ([2012](#_ENREF_31)) #79; derived from Miller, ([1923](#_ENREF_55)) ; Tanaka and Fordyce, (2014, 2015) #66).

(67) Anterior edge of nasals: anterior to, or in line with, anterior edges of supraorbital processes of frontals (0); posterior to anterior edges of supraorbital processes of frontals (1). (Murakami et al. ([2012a](#_ENREF_57), [2012b](#_ENREF_58)) #65; modified from Geisler and Sanders, ([2003](#_ENREF_34)) #80; Geisler et al., ([2011](#_ENREF_33)) #80, ([2012](#_ENREF_31)) #80; Tanaka and Fordyce, (2014, 2015) #67).

(68) Premaxillae in dorsal view: separated anterior to bony nares, exposing mesethmoid (0); joined premaxillae (or maxillae) closing at least posterior part of mesorostral groove (1). (Lambert, ([2005](#_ENREF_45)) #3; Murakami et al. ([2012a](#_ENREF_57), [2012b](#_ENREF_58)) #66; Tanaka and Fordyce, (2014, 2015) #68).

(69) Anterior edge of bony nares: inverted V-shaped, premaxillae gradually converging anteriorly to midline (0); inverted U-shaped, premaxillae abruptly converging anteriorly to midline (1). (Muizon, ([1988](#_ENREF_17)); Geisler and Sanders, ([2003](#_ENREF_34)) #81; Geisler et al., ([2011](#_ENREF_33)) #81, ([2012](#_ENREF_31)) #81; Murakami et al. ([2012a](#_ENREF_57), [2012b](#_ENREF_58)) #67; Tanaka and Fordyce, (2014, 2015) #69).

(70) Fossa for inferior vestibule on maxilla lateral to external nares or lateral to premaxilla: absent (0); present (1). (Muizon, ([1988](#_ENREF_17)); Murakami et al. ([2012a](#_ENREF_57), [2012b](#_ENREF_58)) #68; derived from Curry, ([1992](#_ENREF_13)) ; Tanaka and Fordyce, (2014, 2015) #70).

(71) Maxillary intrusion, anterior to external nares and encroaching the posteromedial or medial face of each premaxilla: absent (0); maxilla visible within opened mesorostral canal as small exposure medially (1); exposure of maxilla reaches dorsally to level of premaxilla and forms a square, rectangular to triangular plate (2); exposure of maxilla reaches dorsally and forms a small subcircular to polygonal ossicle (3). (Muizon, ([1984](#_ENREF_14)) ([1988](#_ENREF_17)); Arnold and Heinsohn,([1996](#_ENREF_3)) #24; Messenger and McGuire, ([1998](#_ENREF_54)) #1422; Murakami et al. ([2012a](#_ENREF_57), [2012b](#_ENREF_58)) #69; Tanaka and Fordyce, (2014, 2015) #71).

(72) Premaxillary crest or posterior maxillary crest adjacent to nasal: absent (0); present (1). (transverse premaxillary crest, sensu Lambert, ([2005](#_ENREF_45)) #6; Murakami et al. ([2012a](#_ENREF_57), [2012b](#_ENREF_58)) #70; Tanaka and Fordyce, (2014, 2015) #72).

(73) Premaxilla: not overhanging itself or maxilla laterally (0); overhanging itself or maxilla laterally, from anterior to midpoint of external nares (1). (Murakami et al. ([2012a](#_ENREF_57), [2012b](#_ENREF_58)) #71; Tanaka and Fordyce, (2014, 2015) #73).

(74) Premaxillary sac fossa: smooth (0); rugose (1). (Messenger and McGuire, ([1998](#_ENREF_54)) #1551; Murakami et al. ([2012a](#_ENREF_57), [2012b](#_ENREF_58)) #72; Tanaka and Fordyce, (2014, 2015) #74).

(75) Ratio of width of right premaxilla to width of left premaxilla in line with midpoint of external nares: 0.90–1.19 (0); 1.20–1.50 (1); 1.50> (2). (Murakami et al. ([2012a](#_ENREF_57), [2012b](#_ENREF_58)) #73; Tanaka and Fordyce, (2014, 2015) #75).

(76) Ratio of greatest width of premaxillae to greatest width of maxillae at level of postorbital processes: ≥0.50 (0); 0.49–0.38 (1); <0.38 (2). (Murakami et al. ([2012a](#_ENREF_57), [2012b](#_ENREF_58)) #74; Tanaka and Fordyce, (2014, 2015) #76).

(77) Premaxillary eminence: absent (0); present but low (1); present and high (2). (Lambert, ([2008](#_ENREF_46)) #4; Murakami et al. ([2012a](#_ENREF_57), [2012b](#_ENREF_58)) #75; modified from Muizon, ([1984](#_ENREF_14)); Barnes, ([1985a](#_ENREF_6)); Heyning, ([1989](#_ENREF_36)) #36, ([1997](#_ENREF_37)) #68; Arnold and Heinsohn,([1996](#_ENREF_3)) #12; Messenger and McGuire, ([1998](#_ENREF_54)) #1410; Geisler and Sanders, ([2003](#_ENREF_34)); #68; Fajardo-Mellor et al., (2006) #2; Geisler et al., ([2011](#_ENREF_33)) #68, ([2012](#_ENREF_31)) #69; derived from Flower, ([1867](#_ENREF_23)); Noble and Fraser, ([1971](#_ENREF_60)) ; Tanaka and Fordyce, (2014, 2015) #77).

(78) Intra-premaxillary foramen on posterior dorsal surface of skull, which is bounded by premaxilla and maxilla: absent (0); present (1) (Tanaka and Fordyce, (2014, 2015) #279)

(79) Right premaxilla: portion anterior to nasal opening wider than portion posterior to opening, with nasal septum angled anteriorly and to left (0); portion posterior to nasal opening wider than portion anterior to opening, with nasal septum angled anteriorly and to right (1). (modified from Geisler and Sanders, ([2003](#_ENREF_34)); #86; Geisler et al., ([2011](#_ENREF_33)) #86, ([2012](#_ENREF_31)) #86; Murakami et al. ([2012a](#_ENREF_57), [2012b](#_ENREF_58)) #77; Tanaka and Fordyce, (2014, 2015) #78).

(80) Left bony naris: same size or slightly larger than right bony naris (0); at least twice the size of right bony naris (1). (Barnes, ([1990](#_ENREF_8)); Geisler and Sanders, ([2003](#_ENREF_34)) #87; Geisler et al., ([2011](#_ENREF_33)) #87, ([2012](#_ENREF_31)) #87; Murakami et al. ([2012a](#_ENREF_57), [2012b](#_ENREF_58)) #78; Tanaka and Fordyce, (2014, 2015) #79).

(81) Supracranial basin: absent (0); present (1). (Heyning, ([1989](#_ENREF_36)) #8, ([1997](#_ENREF_37)) #40; Fordyce, ([1994](#_ENREF_28)) #18; Messenger and McGuire, ([1998](#_ENREF_54)) #1400; Geisler and Sanders, ([2003](#_ENREF_34)) #88; Lambert, ([2005](#_ENREF_45)) #10; Geisler et al., ([2011](#_ENREF_33)) #88, ([2012](#_ENREF_31)) #88; Murakami et al. ([2012a](#_ENREF_57), [2012b](#_ENREF_58)) #79; Tanaka and Fordyce, (2014, 2015) #80).

(82) Proximal ethmoid region: not visible in dorsal view, roofed over by nasals (0); exposed dorsally (1). (Messenger and McGuire, ([1998](#_ENREF_54)) #1455; Geisler and Sanders, ([2003](#_ENREF_34)) #92; Geisler et al., ([2011](#_ENREF_33)) #92, ([2012](#_ENREF_31)) #92; Murakami et al. ([2012a](#_ENREF_57), [2012b](#_ENREF_58)) #80; derived from Miller, ([1923](#_ENREF_55)) ; Tanaka and Fordyce, (2014, 2015) #81).

(83) Mesethmoid: not expanded posterodorsally (0); extended posterodorsally but narrow (1); expanded posterodorsally and visible in lateral view (2). (Murakami et al. ([2012a](#_ENREF_57), [2012b](#_ENREF_58)) #81; modified from Muizon, ([1984](#_ENREF_14), [1988](#_ENREF_17)); Messenger and McGuire, ([1998](#_ENREF_54)) #1454; Bianucci, ([2005](#_ENREF_9)) #9; Tanaka and Fordyce, (2014, 2015) #82).

**Vertex and Area Adjacent to the Nares**

(84) Inflections of ascending processes of premaxillae: gradual (0); vertical (1). (Geisler and Sanders, ([2003](#_ENREF_34)) #107; Geisler et al., ([2011](#_ENREF_33)) #107, ([2012](#_ENREF_31)) #107; modified from Murakami et al. ([2012a](#_ENREF_57), [2012b](#_ENREF_58)) #82; derived from Fordyce, ([1994](#_ENREF_28)) ; Tanaka and Fordyce, (2014, 2015) #83).

(85) Inflections of premaxillae just anterior to, or in line with, anterior edge of supraorbital processes of frontals: absent (0); present (1) (Murakami et al. ([2012a](#_ENREF_57), [2012b](#_ENREF_58)) #83; modified from Geisler and Sanders, ([2003](#_ENREF_34)) #108; Geisler et al., ([2011](#_ENREF_33)) #108, ([2012](#_ENREF_31)) #108; Tanaka and Fordyce, (2014, 2015) #84).

(86) Premaxillary cleft: absent (0); present, posterior part of ascending processes of premaxillae bearing a distinct cleft, originating at posterior edge of premaxillae and continuing anteriorly, dividing each premaxilla into two (1); present, with shallow cleft (2). (Geisler and Sanders, ([2003](#_ENREF_34)) #109; Geisler et al., ([2011](#_ENREF_33)) #109, ([2012](#_ENREF_31)) #109; Murakami et al. ([2012a](#_ENREF_57), [2012b](#_ENREF_58)) #84; Tanaka and Fordyce, (2014, 2015) #85).

(87) Nasal bones: two (0); one or zero (1). (Heyning, ([1989](#_ENREF_36)) #9, ([1997](#_ENREF_37)) #41; Murakami et al. ([2012a](#_ENREF_57), [2012b](#_ENREF_58)) #85; modified from Messenger and McGuire, ([1998](#_ENREF_54)) #1431; Geisler and Sanders, ([2003](#_ENREF_34)) #113; Geisler et al., ([2011](#_ENREF_33)) #113, ([2012](#_ENREF_31)) #113; derived from Kuzmin, ([1976](#_ENREF_44)) ; Tanaka and Fordyce, (2014, 2015) #86).

(88) Nasals: lower than frontals (0); nearly same height as frontals (1); clearly higher than frontals (2). (Muizon, ([1988](#_ENREF_17)); Messenger and McGuire, ([1998](#_ENREF_54)) #1434; Geisler and Sanders, ([2003](#_ENREF_34)); #124; Geisler et al., ([2011](#_ENREF_33)) #124, ([2012](#_ENREF_31)) #124; Murakami et al. ([2012a](#_ENREF_57)) #86; Tanaka and Fordyce, (2014, 2015) #87).

(89) Nasal protuberance: absent (0); present (1). (Muizon, ([1988](#_ENREF_17)); Messenger and McGuire, ([1998](#_ENREF_54)) #1433; Fajardo-Mellor et al., (2006) #7; Lambert, ([2008](#_ENREF_46)) #8; Murakami et al. ([2012a](#_ENREF_57), [2012b](#_ENREF_58)) #87; Tanaka and Fordyce, (2014, 2015) #88).

(90) Both nasals: straight anterior edges in one transverse plane (0); with point on midline and gap on each side between premaxilla and nasal (1); concave posteriorly on midline and gap on each side between premaxilla and nasal (2); concave posteriorly on midline (3). (Murakami et al. ([2012a](#_ENREF_57), [2012b](#_ENREF_58)) #88; modified from Geisler and Sanders, ([2003](#_ENREF_34)) #116; Geisler et al., ([2011](#_ENREF_33)) #116, ([2012](#_ENREF_31)) #116; derived from Moore, ([1968](#_ENREF_56)) ; Tanaka and Fordyce, (2014, 2015) #89).

(91) Nasals: fossae on nasals absent (0); smooth-surfaced fossa on anterior to anterolateral surface (1). (Messenger and McGuire, ([1998](#_ENREF_54)) #1437; Murakami et al. ([2012a](#_ENREF_57), [2012b](#_ENREF_58)) #89; Tanaka and Fordyce, (2014, 2015) #90).

(92) Transverse width of either of nasals as percent maximum length of nasals: very narrow, <20% (0); narrow, 21–69% (1); wide, >70% (2). (Murakami et al. ([2012a](#_ENREF_57)) #90; modified from Muizon, ([1988](#_ENREF_17)); Messenger and McGuire, ([1998](#_ENREF_54)) #1432; Geisler and Sanders, ([2003](#_ENREF_34)); #119; Geisler et al., ([2011](#_ENREF_33)) #119, ([2012](#_ENREF_31)) #119; Tanaka and Fordyce, (2014, 2015) #91).

(93) Nasals: medial portions roughly in same horizontal plane as, or higher than, lateral portions (0); medial portions depressed, forming a median trough immediately posterior to nasal openings (1). (Muizon, ([1988](#_ENREF_17), [1991](#_ENREF_18)); Geisler and Sanders, ([2003](#_ENREF_34)); #118; Geisler et al., ([2011](#_ENREF_33)) #118, ([2012](#_ENREF_31)) #118; Murakami et al. ([2012a](#_ENREF_57), [2012b](#_ENREF_58)) #91; Tanaka and Fordyce, (2014, 2015) #92).

(94) Lateral edges of nasals: not overhanging or covering maxillae or premaxillae (0); overhanging or partly covering maxillae or premaxillae (1). (Murakami et al. ([2012a](#_ENREF_57), [2012b](#_ENREF_58)) #92; Tanaka and Fordyce, (2014, 2015) #93).

(95) Nasal**-**frontal suture: approximately straight transversely (0); anterior wedge (narial process) between frontal posterior ends of nasals (1); W or reversed U suture line (2). (Murakami et al. ([2012a](#_ENREF_57), [2012b](#_ENREF_58)) #93; modified from Muizon, ([1988](#_ENREF_17)); Geisler and Sanders, ([2003](#_ENREF_34)) #121; Geisler et al., ([2011](#_ENREF_33)) #121, ([2012](#_ENREF_31)) #121; Tanaka and Fordyce, (2014, 2015) #94).

(96) Frontals posterior to nasals and between premaxillae: wider than maximum transverse width across nasals (0); same as transverse width of nasals (1); narrower than transverse width of nasals, maxillae expanded medially posterior to nasals (2). (Geisler and Sanders, ([2003](#_ENREF_34))#125; Geisler et al., ([2011](#_ENREF_33)) #125, ([2012](#_ENREF_31)) #125; Murakami et al. ([2012a](#_ENREF_57), [2012b](#_ENREF_58)) #94; modified from Messenger and McGuire, ([1998](#_ENREF_54)) #1457; Tanaka and Fordyce, (2014, 2015) #95).

(97) Frontal boss on vertex: absent (0); present (1). (Muizon, ([1984](#_ENREF_14), [1988](#_ENREF_17)); Messenger and McGuire, ([1998](#_ENREF_54)) #1461; Fajardo-Mellor et al., (2006) #12; Murakami et al. ([2012a](#_ENREF_57), [2012b](#_ENREF_58)) #95; modified from Lambert, ([2008](#_ENREF_46)) #9; Tanaka and Fordyce, (2014, 2015) #96).

(98) Vertex: absent (0); present (1); highly developed (2). (Murakami et al. ([2012a](#_ENREF_57)) #96; Muizon, ([1991](#_ENREF_18)); Messenger and McGuire, ([1998](#_ENREF_54)) #1404; Lambert, ([2005](#_ENREF_45)) #7; Tanaka and Fordyce, (2014, 2015) #97).

(99) Cranial vertex skewed asymmetrically to left side: absent (0); present (1). (Barnes, ([1990](#_ENREF_8)); Bianucci, ([2005](#_ENREF_9)) #7; Aguirre-Fernandez et al., ([2009](#_ENREF_1)) #18; Murakami et al. ([2012a](#_ENREF_57), [2012b](#_ENREF_58)) #97; Tanaka and Fordyce, (2014, 2015) #98).

(100) Anterodorsal wall of braincase: formed by frontals (0); mostly formed by maxillae (1). (Geisler and Sanders, ([2003](#_ENREF_34)) #127; Geisler et al., ([2011](#_ENREF_33)) #127, ([2012](#_ENREF_31)) #127; Murakami et al. ([2012a](#_ENREF_57), [2012b](#_ENREF_58)) #98; derived from Schulte, ([1917](#_ENREF_68)); Miller, ([1923](#_ENREF_55)) ; Tanaka and Fordyce, (2014, 2015) #99).

(101) Nuchal crest: higher than frontals and/or nasals (0); at same level as frontals and/or nasals (1); below frontals and/or nasals (2). (Murakami et al. ([2012a](#_ENREF_57), [2012b](#_ENREF_58)) #99; modified from Geisler and Sanders, ([2003](#_ENREF_34)) #128; derived from Moore, ([1968](#_ENREF_56)) ; Tanaka and Fordyce, (2014, 2015) #100).

**Temporal Fossae, Zygomatic Arch, and Occipitals**

(102) Temporal fossa shape in lateral view: height lower than anteroposterior length (0); higher (1); nearly equilateral square (2); lower and its posterior end is rounded (3). (Tanaka and Fordyce, (2014, 2015) #281)

(103) Temporal fossa: not roofed over by lateral expansion of maxillae (0); roofed over by lateral expansion of maxillae (1). (Muizon, ([1988](#_ENREF_17)); Heyning, ([1989](#_ENREF_36)) #22, ([1997](#_ENREF_37)) #54; Arnold and Heinsohn,([1996](#_ENREF_3)) #39; Messenger and McGuire, ([1998](#_ENREF_54)) #1453; Murakami et al. ([2012a](#_ENREF_57), [2012b](#_ENREF_58)) #100; Tanaka and Fordyce, (2014, 2015) #101).

(104) Roof of temporal fossa formed by: frontals (0); frontals, but with large opening through maxillae and/or premaxillae exposing margins of window formed by a frontal ring (1). (Geisler and Sanders, ([2003](#_ENREF_34)) #132; Geisler et al., ([2011](#_ENREF_33)) #132, ([2012](#_ENREF_31)) #132; Murakami et al. ([2012a](#_ENREF_57), [2012b](#_ENREF_58)) #101; Tanaka and Fordyce, (2014, 2015) #102).

(105) Position and orientation of origin for temporal muscle on supraorbital process of frontal: origin laying on posterior face of supraorbital process and directed roughly posteriorly (0); origin lying on posteroventral face of supraorbital process and directed roughly ventrally (1). (Fordyce, ([1994](#_ENREF_28)) #8; Lambert, ([2005](#_ENREF_45)) #23; Murakami et al. ([2012a](#_ENREF_57), [2012b](#_ENREF_58)) #102; Tanaka and Fordyce, (2014, 2015) #103).

(106) Parietal dorsally: not fused to frontal or supraoccipital (0); completely fused to, and indistinguishable from, frontal or supraoccipital (1). (Murakami et al. ([2012a](#_ENREF_57), [2012b](#_ENREF_58)) #103; Tanaka and Fordyce, (2014, 2015) #104).

(107) Parietals in dorsal view: contacting each other on the midline or separated by interparietal (0); in skull roof but visible only as small triangular areas at edges of intertemporal constriction, with supraoccipital overlapping and obscuring median portions (1); completely absent in skull roof (2); visible only as triangular areas, dorsolateral to supraoccipital, with non-overlapping supraoccipital separated from and contacting parietals along irregular suture (3). (Geisler and Sanders, ([2003](#_ENREF_34)) #134; Geisler et al., ([2011](#_ENREF_33)) #134, ([2012](#_ENREF_31)) #134; Murakami et al. ([2012a](#_ENREF_57), [2012b](#_ENREF_58)) #104; derived from Whitmore and Sanders, ([1977](#_ENREF_73)); Barnes, ([1990](#_ENREF_8)); modified from Lambert, ([2005](#_ENREF_45)) #15; Tanaka and Fordyce, (2014, 2015) #105).

(108) Interparietal: present (0); absent or fused and therefore not distinguishable from parietals and frontals (1). (Geisler and Sanders, ([2003](#_ENREF_34)) #135; Geisler et al., ([2011](#_ENREF_33)) #135, ([2012](#_ENREF_31)) #135; Murakami et al. ([2012a](#_ENREF_57)) #105; Tanaka and Fordyce, (2014, 2015) #106).

(109) Sagittal crest for temporal muscle: present (0); absent (1). (Murakami et al. ([2012a](#_ENREF_57), [2012b](#_ENREF_58)) #106; modified from Geisler and Sanders, ([2003](#_ENREF_34)) #136; Geisler et al., ([2011](#_ENREF_33)) #136, ([2012](#_ENREF_31)) #136; Tanaka and Fordyce, (2014, 2015) #107).

(110) Alisphenoid: broadly exposed laterally in temporal fossa (0); lateral surface broadly overlapped by parietal, with a narrow strip visible or invisible on ventral edge of temporal fossa in lateral view (1). (Geisler and Sanders, ([2003](#_ENREF_34)) #141; Geisler et al., ([2011](#_ENREF_33)) #141, ([2012](#_ENREF_31)) #141; Murakami et al. ([2012a](#_ENREF_57)) #107; Tanaka and Fordyce, (2014, 2015) #108).

(111) Anterior zygomatic process end of squmosal in lateral view: taipered (0); squared (1). (Tanaka and Fordyce, (2014, 2015) #282)

(112) Zygomatic process of squamosal: directed anterolaterally (0); directed anteriorly (1). (Sanders and Barnes, 2002; Geisler and Sanders, ([2003](#_ENREF_34)) #142; Geisler et al., ([2011](#_ENREF_33)) #142, ([2012](#_ENREF_31)) #142; Murakami et al. ([2012a](#_ENREF_57), [2012b](#_ENREF_58)) #108; Tanaka and Fordyce, (2014, 2015) #109).

(113) Zygomatic process of squamosal in lateral view: part of dorsal face visible (0); entire dorsal surface of squamosal visible (1). (Murakami et al. ([2012a](#_ENREF_57), [2012b](#_ENREF_58)) #109; Tanaka and Fordyce, (2014, 2015) #110).

(114) Emargination of posterior edge of zygomatic process by neck muscle fossa, skull in lateral view: absent, posterior edge forming nearly right angle with dorsal edge of zygomatic process of squamosal (0); shallow emargination (1); deep emargination (2). (Geisler and Sanders, ([2003](#_ENREF_34)) #144; Geisler et al., ([2011](#_ENREF_33)) #144, ([2012](#_ENREF_31)) #144; Murakami et al. ([2012a](#_ENREF_57), [2012b](#_ENREF_58)) #110; Tanaka and Fordyce, (2014, 2015) #111).

(115) Width of squamosal lateral to exoccipital as percent greatest width of exoccipitals, skull in posterior view: exposed portion of squamosal narrow, <15% (0); moderate, 16–35% (1). (modified from Geisler and Sanders, ([2003](#_ENREF_34)) #145; Geisler et al., ([2011](#_ENREF_33)) #145, ([2012](#_ENREF_31)) #145; Murakami et al. ([2012a](#_ENREF_57), [2012b](#_ENREF_58)) #111; Tanaka and Fordyce, (2014, 2015) #112).

(116) Postglenoid process of squamosal: not reduced (0); greatly reduced (1). (Murakami et al. ([2012a](#_ENREF_57), [2012b](#_ENREF_58)) #113; Tanaka and Fordyce, (2014, 2015) #114).

(117) Postglenoid process in lateral view: tapering ventrally (0); squared off ventrally (1); same as state 1 except very wide anteroposterior diameter of process (2). (Geisler and Sanders, ([2003](#_ENREF_34)) #151; Lambert, ([2005](#_ENREF_45)) #24; Geisler et al., ([2011](#_ENREF_33)) #151, ([2012](#_ENREF_31)) #151; Murakami et al. ([2012a](#_ENREF_57), [2012b](#_ENREF_58)) #114; derived from Muizon, ([1991](#_ENREF_18)) ; Tanaka and Fordyce, (2014, 2015) #115).

(118) Relative ventral projections of postglenoid and post-tympanic processes of squamosal: postglenoid process more ventral or at same level as post-tympanic process (0); apex of postglenoid process dorsally higher than post-tympanic process (1). (Lambert, ([2005](#_ENREF_45)) #25; Murakami et al. ([2012a](#_ENREF_57)). b #115; Tanaka and Fordyce, (2014, 2015) #116).

(119) Nuchal crest in dorsoposterior view: semicircular, pointed anteriorly (0); rectangular or weakly convex anteriorly or posteriorly (1); convex posteriorly and/or midpoint convex triangular and pointed anteriorly (2); prominently convex anteriorly (3); strongly convex posteriorly (4). (Murakami et al. ([2012a](#_ENREF_57), [2012b](#_ENREF_58)) #116; modified from Geisler and Sanders, ([2003](#_ENREF_34)); #152; Geisler et al., ([2011](#_ENREF_33)) #152, ([2012](#_ENREF_31)) #152; derived from Barnes, ([1985b](#_ENREF_7)) ; Tanaka and Fordyce, (2014, 2015) #117).

(120) Occipital shield: smoothly convex or concave (0); bearing distinct sagittal crest (1). (Sanders and Barnes, ([2002](#_ENREF_66)); Geisler and Sanders, ([2003](#_ENREF_34)) #155; Geisler et al., ([2011](#_ENREF_33)) #155, ([2012](#_ENREF_31)) #155; Murakami et al. ([2012a](#_ENREF_57), [2012b](#_ENREF_58)) #117; Tanaka and Fordyce, (2014, 2015) #118).

(121) Dorsal condyloid fossa: absent (0); present, situated anterodorsal to dorsal edge of condyle (1); present and forming deep pit (2). (Geisler and Sanders, ([2003](#_ENREF_34)) #156; Geisler et al., ([2011](#_ENREF_33)) #156, ([2012](#_ENREF_31)) #156; Murakami et al. ([2012a](#_ENREF_57), [2012b](#_ENREF_58)) #118; derived from Sanders and Barnes, ([2002](#_ENREF_66)) ; Tanaka and Fordyce, (2014, 2015) #119).

**Anterior Basicranium**

(122) Palatine in nasal passage: thin, forming posterior part of nasal passage (0); thick, forming part of anterior wall of nasal cavities (1); palatine does not join anterior wall of nasal passage (2). (Murakami et al. ([2012a](#_ENREF_57), [2012b](#_ENREF_58)) #119; modified from Geisler and Sanders, ([2003](#_ENREF_34)); #158; Geisler et al., ([2011](#_ENREF_33)) #158, ([2012](#_ENREF_31)) #158; derived from Miller, ([1923](#_ENREF_55)) ; Tanaka and Fordyce, (2014, 2015) #120).

(123) Palatine exposure: exposed ventrally (0); partially covered by pterygoid, which divides it into medial and lateral exposures (1); ventral surfaces completely covered by pterygoids (2). (Muizon, ([1987](#_ENREF_16)); Arnold and Heinsohn,([1996](#_ENREF_3)) #15; Messenger and McGuire, ([1998](#_ENREF_54)) #1440; Geisler and Sanders, ([2003](#_ENREF_34)) #159; Lambert, ([2005](#_ENREF_45)) #27; Geisler et al., ([2011](#_ENREF_33)) #159, ([2012](#_ENREF_31)) #159; Murakami et al. ([2012a](#_ENREF_57), [2012b](#_ENREF_58)) #120; derived from Miller, ([1923](#_ENREF_55)) ; Tanaka and Fordyce, (2014, 2015) #121).

(124) Lateral lamina of palatine: absent (0); present (1). (Muizon, ([1984](#_ENREF_14), [1988](#_ENREF_17)), ([1991](#_ENREF_18)); Arnold and Heinsohn,([1996](#_ENREF_3)) #16; Messenger and McGuire, ([1998](#_ENREF_54)) #1443; Murakami et al. ([2012a](#_ENREF_57), [2012b](#_ENREF_58)) #121; Tanaka and Fordyce, (2014, 2015) #122).

(125) Lateral lamina of palatine relationship with maxilla: free from or sutured to maxilla (0); fused to maxilla (1). (Muizon, ([1988](#_ENREF_17)); Messenger and McGuire, ([1998](#_ENREF_54)) #1439; Geisler and Sanders, ([2003](#_ENREF_34)) #161; Geisler et al., ([2011](#_ENREF_33)) #161, ([2012](#_ENREF_31)) #161; Murakami et al. ([2012a](#_ENREF_57), [2012b](#_ENREF_58)) #122; Tanaka and Fordyce, (2014, 2015) #123).

(126) Lateral lamina of palatine relationship with orbit: does not form bony bridge “over” (= ventral to) orbit (0); does form bony bridge “over” (= ventral to) orbit (1). (Muizon, ([1984](#_ENREF_14)); Messenger and McGuire, ([1998](#_ENREF_54)) #1444; Murakami et al. ([2012a](#_ENREF_57), [2012b](#_ENREF_58)) #123; Tanaka and Fordyce, (2014, 2015) #124).

(127) Pterygoids in anteroventral view: separated from each other by posteroventrally elongated palatines and/or vomer (0); contacting entire length of hamular process (1); contacting each other partially (2). (Murakami et al. ([2012a](#_ENREF_57), [2012b](#_ENREF_58)) #124; modified from Arnold and Heinsohn,([1996](#_ENREF_3)) #5; Messenger and McGuire, ([1998](#_ENREF_54)) #1445; Fajardo-Mellor et al., (2006) #9; derived from Flower, ([1884](#_ENREF_26)); Barnes, ([1985a](#_ENREF_6)); Marsh et al., ([1989](#_ENREF_49)) ; Tanaka and Fordyce, (2014, 2015) #125).

(128) Medial pterygoid**-**palatine suture in ventral view: angled anteromedially (0); nearly transverse (1); angled anterolaterally (2); angled anteroposteriorly (3). (Murakami et al. ([2012a](#_ENREF_57), [2012b](#_ENREF_58)) #125; modified from Geisler and Sanders, ([2003](#_ENREF_34)) #162; Geisler et al., ([2011](#_ENREF_33)) #162, ([2012](#_ENREF_31)) #162; Tanaka and Fordyce, (2014, 2015) #126).

(129) Lateral lamina of pterygoid: absent (0); present and articulated with alisphenoid (1); partial, restricted to region lateral to hamular process (2). (Murakami et al. ([2012a](#_ENREF_57)) #126; modified from Arnold and Heinsohn,([1996](#_ENREF_3)) #121; Messenger and McGuire, ([1998](#_ENREF_54)) #1446; Geisler and Sanders, ([2003](#_ENREF_34)) #164; Lambert, ([2005](#_ENREF_45)) #32; Geisler et al., ([2011](#_ENREF_33)) #164, ([2012](#_ENREF_31)) #164; derived from Miller, ([1923](#_ENREF_55)); Kellogg, ([1936](#_ENREF_42)); Fraser and Purves, ([1960](#_ENREF_30)) ; Tanaka and Fordyce, (2014, 2015) #127).

(130) Subtemporal crest: present (0); present but reduced, or absent (1). (modified from Geisler and Sanders, ([2003](#_ENREF_34)) #165; Geisler et al., ([2011](#_ENREF_33)) #165, ([2012](#_ENREF_31)) #165; Murakami et al. ([2012a](#_ENREF_57), [2012b](#_ENREF_58)) #127; Tanaka and Fordyce, (2014, 2015) #128).

(131) Superior lamina of pterygoid: absent from sphenoidal region but present in orbital region (0); present and covers most of ventral exposure of alisphenoid (1); partially absent from orbital region (2); completely absent from orbital region (3). (Murakami et al. ([2012a](#_ENREF_57), [2012b](#_ENREF_58)) #128; modified from Arnold and Heinsohn,([1996](#_ENREF_3)) #16; Geisler and Sanders, ([2003](#_ENREF_34)) #167; Geisler et al., ([2011](#_ENREF_33)) #167, ([2012](#_ENREF_31)) #167; derived from Miller ([1923](#_ENREF_55)); Fraser and Purves, ([1960](#_ENREF_30))) ; Tanaka and Fordyce, (2014, 2015) #129.

(132) Pterygoids excavated anterior to choanae by the pterygoid sinuses, with distinct anterior fossa clearly limited forwards by rounded wall: absent (0); present (1). (Lambert, ([2005](#_ENREF_45)) #28; Murakami et al. ([2012a](#_ENREF_57), [2012b](#_ENREF_58)) #129; Tanaka and Fordyce, (2014, 2015) #130).

(133) Depth of pterygoid sinus fossa in basicranium: shallow or partially excavated (0); deep, excavated dorsally to level of cranial foramen oval (1); deep, and extended dorsally into orbit (2). (modified from Fordyce, ([1994](#_ENREF_28)) #6; Lambert, ([2005](#_ENREF_45)) #30; Murakami et al. ([2012a](#_ENREF_57), [2012b](#_ENREF_58)) #130; Tanaka and Fordyce, (2014, 2015) #131).

(134) Anterior level of pterygoid sinus fossa: interrupted posterior to, or the level of, antorbital notch (0); extending beyond the level of the antorbital notch (1). (Lambert, ([2005](#_ENREF_45)) #29; Murakami et al. ([2012a](#_ENREF_57), [2012b](#_ENREF_58)) #131; Tanaka and Fordyce, (2014, 2015) #132).

(135) Preorbital and postorbital fossae of pterygoid sinuses: widened apices of preorbital and postorbital fossae of pterygoid sinuses present but fossae not merged or fused (0); widened apices of preorbital and postorbital fossae of pterygoid sinuses merged or fused dorsal to path of optic nerve (1). (Murakami et al. ([2012a](#_ENREF_57), [2012b](#_ENREF_58)) #132; modified from Muizon, ([1988](#_ENREF_17)); Arnold and Heinsohn,([1996](#_ENREF_3)) #19; Bianucci, ([2005](#_ENREF_9)) #10; Aguirre-Fernandez et al., ([2009](#_ENREF_1)) #19; Tanaka and Fordyce, (2014, 2015) #133).

(136) Fossa for preorbital lobe of pterygoid sinus in orbit: absent (0); present (1). (Fraser and Purves, ([1960](#_ENREF_30)); Arnold and Heinsohn,([1996](#_ENREF_3)) #18; Murakami et al. ([2012a](#_ENREF_57), [2012b](#_ENREF_58)) #133; Tanaka and Fordyce, (2014, 2015) #134).

(137) Dorsal development of fossa for preorbital lobe of pterygoid sinus toward the frontal**-**maxilla suture: absent (0); present (1). (Muizon, ([1984](#_ENREF_14), [1988](#_ENREF_17)); Heyning, ([1989](#_ENREF_36)) #37, ([1997](#_ENREF_37)) #69; Messenger and McGuire, ([1998](#_ENREF_54)) #1460; Arnold and Heinsohn,([1996](#_ENREF_3)) #20; Lambert, ([2008](#_ENREF_46)) #13; Murakami et al. ([2012a](#_ENREF_57), [2012b](#_ENREF_58)) #134; modified from Fajardo-Mellor et al., (2006) #13; derived from Fraser and Purves, ([1960](#_ENREF_30)) ; Tanaka and Fordyce, (2014, 2015) #135).

(138) Postorbital lobe of pterygoid sinus fossa: absent (0); present (1). (Arnold and Heinsohn,([1996](#_ENREF_3)) #18; Geisler and Sanders, ([2003](#_ENREF_34)) #170; Geisler et al., ([2011](#_ENREF_33)) #170, ([2012](#_ENREF_31)) #170; Murakami et al. ([2012a](#_ENREF_57), [2012b](#_ENREF_58)) #135; derived from Fraser and Purves, ([1960](#_ENREF_30)) ; Tanaka and Fordyce, (2014, 2015) #136).

(139) Anteroposteriorly elongated pterygoid sinus fossa, at level of orbit, bordered by mediolaterally compressed subtemporal crest of frontal: absent (0); present (1). (Murakami et al. ([2012a](#_ENREF_57), [2012b](#_ENREF_58)) #136; Tanaka and Fordyce, (2014, 2015) #137).

(140) Orbitosphenoid: not contacting lacrimal or lacrimojugal (0); contacting lacrimal or lacrimojugal (1). (Murakami et al. ([2012a](#_ENREF_57), [2012b](#_ENREF_58)) #137; Tanaka and Fordyce, (2014, 2015) #138).

(141) Ratio of length of hamular process of pterygoid to cranium length: <0.30 (0); 0.30–0.44 (1); 0.45–0.59 (2); >0.60 (3). The length of the hamular process of the pterygoid is measured from anterior edge of the pterygoid to posterior edge of the hamular process. The cranium length is measured from anterior edge of the antorbital process to posterior edge of occipital condyles. (Murakami et al. ([2012a](#_ENREF_57), [2012b](#_ENREF_58)) #138; modified from Heyning, ([1989](#_ENREF_36)) #18, ([1997](#_ENREF_37)) #50; Muizon, ([1991](#_ENREF_18)); Messenger and McGuire, ([1998](#_ENREF_54)) #1447; Lambert, ([2005](#_ENREF_45)) #31; Tanaka and Fordyce, (2014, 2015) #139).

(142) Keel affecting ventral surfaces of hamular processes: absent (0); present (1). (Muizon, ([1988](#_ENREF_17)); Messenger and McGuire, ([1998](#_ENREF_54)) #1449; Bianucci, ([2005](#_ENREF_9)) #14; Murakami et al. ([2012a](#_ENREF_57), [2012b](#_ENREF_58)) #139; modified from Fajardo-Mellor et al., (2006) #10; Tanaka and Fordyce, (2014, 2015) #140).

(143) Exposure of medial lamina of pterygoid hamuli in lateral view: complete or broad exposure due to extreme reduction of lateral lamina of pterygoid hamuli (0); no exposure due to a posterior extension of lateral lamina extending posterior to medial lamina (1); medial lamina of pterygoid hamuli exposing lateral lamina through ovoid window in lateral view (2). (Muizon, ([1988](#_ENREF_17)); Fajardo-Mellor et al., (2006) #11; Murakami et al. ([2012a](#_ENREF_57), [2012b](#_ENREF_58)) #140; derived from Noble and Fraser, ([1971](#_ENREF_60)) ; Tanaka and Fordyce, (2014, 2015) #141).

(144) Shape of restricted area between postorbital ridge of frontal and subtemporal crest from ventral view: anteroposteriorly long elliptical (0); wide fan-shape (1); narrow fan-shape (2), rhombus (3). (Tanaka and Fordyce, (2014, 2015) #280)

**Posterior Basicranium**

(145) Falciform process of squamosal: plate-like with anteroposteriorly wide base (0); rod-like with narrow base (1); poorly developed or absent (2). (Geisler and Sanders, ([2003](#_ENREF_34)) #176; Geisler et al., ([2011](#_ENREF_33)) #176, ([2012](#_ENREF_31)) #176; Murakami et al. ([2012a](#_ENREF_57), [2012b](#_ENREF_58)) #141; modified from Lambert, ([2005](#_ENREF_45)) #36; Tanaka and Fordyce, (2014, 2015) #142).

(146) Falciform process of squamosal: medial surface not sutured to lateral lamina of pterygoid (0); medial surface sutured to lateral lamina of pterygoid (1). (Murakami et al. ([2012a](#_ENREF_57), [2012b](#_ENREF_58)) #142; modified from Geisler and Sanders, ([2003](#_ENREF_34)) #177; Geisler et al., ([2011](#_ENREF_33)) #177, ([2012](#_ENREF_31)) #177; Tanaka and Fordyce, (2014, 2015) #143).

(147) Tympanosquamosal recess: absent, with anterior transverse ridge present (0); anterior transverse ridge absent and middle sinus inferred to be present without a large tympanosquamosal recess (1); present and enlarged, forming triangular fossa medial and anteromedial to postglenoid process (2); very large, forming large fossa bordering entire medial edge of glenoid fossa (3). (Geisler and Sanders, ([2003](#_ENREF_34)) #178; Geisler et al., ([2011](#_ENREF_33)) #178, ([2012](#_ENREF_31)) #178; Murakami et al. ([2012a](#_ENREF_57), [2012b](#_ENREF_58)) #143; modified from Lambert, ([2005](#_ENREF_45)) #35; derived from Fraser and Purves, ([1960](#_ENREF_30)), and Fordyce, ([2002](#_ENREF_29)) ; Tanaka and Fordyce, (2014, 2015) #144).

(148) Bifurcation of tympanosquamosal recess: absent, almost undeveloped, elliptic (0); present, with a clear expansion anteriorly, invasion of mandibular fossa medially, and a depression (expansion) at the postglenoid process posteriorly (1). (Murakami et al. ([2012a](#_ENREF_57), [2012b](#_ENREF_58)) #144; modified from Muizon, ([1988](#_ENREF_17)); Bianucci, ([2005](#_ENREF_9)) #11; Aguirre-Fernandez et al., ([2009](#_ENREF_1)) #20; Tanaka and Fordyce, (2014, 2015) #145).

(149) Fossa for the basisphenoidal sinus: absent (0); present (1). (Fraser and Purves, ([1960](#_ENREF_30)); Mead and Fordyce, ([2009](#_ENREF_52)); Murakami et al. ([2012a](#_ENREF_57), [2012b](#_ENREF_58)) #145; Tanaka and Fordyce, (2014, 2015) #146).

(150) Position of more-distal part of alisphenoid**-**squamosal suture, with skull in ventral view: anterior to external opening of foramen oval or a homologous groove (0); courses along groove for mandibular branch of trigeminal nerve, or just posterior to it (1); just medial to anterior edge of floor of squamosal fossa, foramen ovale, and/or groove situated entirely on alisphenoid (2). (Geisler and Sanders, ([2003](#_ENREF_34)) #180; Geisler et al., ([2011](#_ENREF_33)) #180, ([2012](#_ENREF_31)) #180; Murakami et al. ([2012a](#_ENREF_57), [2012b](#_ENREF_58)) #146; Tanaka and Fordyce, (2014, 2015) #147).

(151) Groove for mandibular branch of trigeminal nerve: lateral end of groove wrapping laterally around posterior end of pterygoid sinus fossa and opening primarily anteriorly (0); directed laterally and located entirely posterior to pterygoid sinus fossa (1). (Murakami et al. ([2012a](#_ENREF_57), [2012b](#_ENREF_58)) #147; modified from Geisler and Sanders, ([2003](#_ENREF_34)) #181; Geisler et al., ([2011](#_ENREF_33)) #181, ([2012](#_ENREF_31)) #181; Tanaka and Fordyce, (2014, 2015) #148).

(152) Suprameatal pit of squamosal: absent (0); present but shallow, situated dorsolateral to spiny process of squamosal (1); forming deep dorsolateral excavation into squamosal (2). (Geisler and Sanders, ([2003](#_ENREF_34)) #185; Geisler et al., ([2011](#_ENREF_33)) #185, ([2012](#_ENREF_31)) #185; Murakami et al. ([2012a](#_ENREF_57), [2012b](#_ENREF_58)) #149; Tanaka and Fordyce, (2014, 2015) #149).

(153) Foramen spinosum: absent (0); present, located in anteromedial corner of anterior part of periotic fossa near or on squamosal**-**parietal suture (1). (Muizon, ([1994](#_ENREF_19)); Geisler and Sanders, ([2003](#_ENREF_34))#186; Geisler et al., ([2011](#_ENREF_33)) #186, ([2012](#_ENREF_31)) #186; Murakami et al. ([2012a](#_ENREF_57), [2012b](#_ENREF_58)) #150; Tanaka and Fordyce, (2014, 2015) #150).

(154) Posterior portion of periotic fossa of squamosal: fossa absent (0); fossa present but shallow (1); highly compressed fossa forming narrow slit or small blind foramen (2); posteromedial portion contains large deep fossa (3). (Geisler and Sanders, ([2003](#_ENREF_34)) #187; Geisler et al., ([2011](#_ENREF_33)) #187, ([2012](#_ENREF_31)) #187; Murakami et al. ([2012a](#_ENREF_57), [2012b](#_ENREF_58)) #149 and #151; Tanaka and Fordyce, (2014, 2015) #151).

(155) Length of zygomatic process of squamosal as percent of greatest width of maxilla at postorbital process: >31% (0); ≤30% (1). (Murakami et al. ([2012a](#_ENREF_57), [2012b](#_ENREF_58)) #152; modified from Heyning, ([1989](#_ENREF_36)) #33, 35, #65, 67; Geisler and Sanders, ([2003](#_ENREF_34)) #188; Geisler et al., ([2011](#_ENREF_33)) #188, ([2012](#_ENREF_31)) #188; Tanaka and Fordyce, (2014, 2015) #152)

(156) External auditory meatus: wide (0); narrow (1). (Fordyce, ([1994](#_ENREF_28)) #10; Geisler and Sanders, ([2003](#_ENREF_34)) #189, ([2012](#_ENREF_31)) #189; Lambert, ([2005](#_ENREF_45)) #26; Geisler et al., ([2011](#_ENREF_33)) #189; Murakami et al. ([2012a](#_ENREF_57), [2012b](#_ENREF_58)) #153).

(157) Vomer: posterior edge terminating on or at anterior edge of basisphenoid (0); terminating on basioccipital, covering basioccipital**-**basisphenoid suture ventrally (1). (Barnes, ([1984b](#_ENREF_5)); Geisler and Sanders, ([2003](#_ENREF_34)) #190; Geisler et al., ([2011](#_ENREF_33)) #190, ([2012](#_ENREF_31)) #190; Murakami et al. ([2012a](#_ENREF_57), [2012b](#_ENREF_58)) #154; Tanaka and Fordyce, (2014, 2015) #153).

(158) Rectus capitus anticus muscle fossa: absent or poorly developed (0); present with well-defined anterior edge (1). (Geisler and Sanders, ([2003](#_ENREF_34)) #192; Geisler et al., ([2011](#_ENREF_33)) #192, ([2012](#_ENREF_31)) #192; Murakami et al. ([2012a](#_ENREF_57), [2012b](#_ENREF_58)) #155; Tanaka and Fordyce, (2014, 2015) #155).

(159) Posteroventral-most point of basioccipital crest: rounded over (0); forming closely appressed separate flange, with narrow crease separating exoccipital dorsally from rest of basioccipital crest (1); projecting distinct flange posteriorly (2); distinct but separated by pronounced notch, interrupting basioccipital crest (3). (Geisler and Sanders, ([2003](#_ENREF_34)) #193; Geisler et al., ([2011](#_ENREF_33)) #193, ([2012](#_ENREF_31)) #193; Murakami et al. ([2012a](#_ENREF_57), [2012b](#_ENREF_58)) #156; Tanaka and Fordyce, (2014, 2015) #156).

(160) Angle formed by basioccipital crests in ventral view: parallel with no angle formed (0); ca. 15–40° (1); ca. 42–68° (2); ca. 70–90° (3); >100° (4). (Murakami et al. ([2012a](#_ENREF_57), [2012b](#_ENREF_58)) #157; modified from Geisler and Sanders, ([2003](#_ENREF_34)) #194; Geisler et al., ([2011](#_ENREF_33)) #194, ([2012](#_ENREF_31)) #194; Tanaka and Fordyce, (2014, 2015) #157).

(161) Basioccipital width compared with maximum width of skull in ventral view: narrow, less than 50% (0), wider, larger than 51% (1). (Tanaka and Fordyce, (2014, 2015) #283)

(162) Hypoglossal foramen: separated from jugular foramen, or jugular notch, by thick bone (0); separated by very thin bone or absent, in latter case hypoglossal foramen becoming confluent with jugular foramen (1). (Geisler and Sanders, ([2003](#_ENREF_34)) #195; Geisler et al., ([2011](#_ENREF_33)) #195, ([2012](#_ENREF_31)) #195; Murakami et al. ([2012a](#_ENREF_57), [2012b](#_ENREF_58)) #158; Tanaka and Fordyce, (2014, 2015) #158).

(163) Jugular notch, gap between paroccipital process and basioccipital crest: open notch, width of opening and depth of notch about equal (0); narrow and almost slit-like, depth much greater than width of opening (1). (Geisler and Sanders, ([2003](#_ENREF_34)) #196; Geisler et al., ([2011](#_ENREF_33)) #196, ([2012](#_ENREF_31)) #196; Murakami et al. ([2012a](#_ENREF_57), [2012b](#_ENREF_58)) #159; Tanaka and Fordyce, (2014, 2015) #159; Tanaka and Fordyce, (2014, 2015) #159).

(164) Paroccipital process, skull in ventral view: posterior edge located well anterior to the posterior edge of condyle (0); posterior edge in transverse line with posterior edge of condyle (1). (Geisler and Sanders, ([2003](#_ENREF_34)) #197; Geisler et al., ([2011](#_ENREF_33)) #197, ([2012](#_ENREF_31)) #197; Murakami et al. ([2012a](#_ENREF_57), [2012b](#_ENREF_58)) #160; Tanaka and Fordyce, (2014, 2015) #160).

(165) Fossa for posterior sinus in exoccipital: absent or slightly concave (0); moderately concave (1); forming deep sack-like structure (2). (Murakami et al. ([2012a](#_ENREF_57), [2012b](#_ENREF_58)) #161; modified from Muizon, ([1991](#_ENREF_18)); Lambert, ([2005](#_ENREF_45)) #38; Tanaka and Fordyce, (2014, 2015) #161).

(166) Occipital condyles; on pedicle (0); lacking pedicle, unified with occipital (1). (Tanaka and Fordyce, (2014, 2015) #284)

**Malleus**

(167) Tuberculum of malleus: unreduced (0); highly reduced, almost indistinguishable from articular head (1). (Muizon, ([1985](#_ENREF_15)); Messenger and McGuire, ([1998](#_ENREF_54)) #1499; Geisler and Sanders, ([2003](#_ENREF_34)) #198; Geisler et al., ([2011](#_ENREF_33)) #198, ([2012](#_ENREF_31)) #198; Murakami et al. ([2012a](#_ENREF_57), [2012b](#_ENREF_58)) #162; modified from Lambert, ([2005](#_ENREF_45)) #69. derived from Doran, ([1878](#_ENREF_21)) ; Tanaka and Fordyce, (2014, 2015) #162).

(168) Processus muscularis of malleus: shorter than manubrium of malleus (0); sub-equal or longer than manubrium (1). (Murakami et al. ([2012a](#_ENREF_57), [2012b](#_ENREF_58)) #163; modified from Muizon, ([1985](#_ENREF_15); [1988](#_ENREF_17)); Messenger and McGuire, ([1998](#_ENREF_54)) #1550; Geisler and Sanders, ([2003](#_ENREF_34)) #199; Lambert, ([2005](#_ENREF_45)) #70; Geisler et al., ([2011](#_ENREF_33)) #199, ([2012](#_ENREF_31)) #199; Tanaka and Fordyce, (2014, 2015) #163).

**Periotic**

(169) Length of anterior process of periotic as percent length of pars cochlearis: short, <59% (0); long, >60% (1). (Murakami et al. ([2012a](#_ENREF_57), [2012b](#_ENREF_58)) #164; modified from Muizon, ([1988](#_ENREF_17)); Heyning, ([1989](#_ENREF_36)) #5; Messenger and McGuire, ([1998](#_ENREF_54)) #1489; Geisler and Luo, ([1996](#_ENREF_32)) #1; Luo and Marsh, ([1996](#_ENREF_48)) #24; Geisler and Sanders, ([2003](#_ENREF_34)) #203; Lambert, ([2005](#_ENREF_45)) #39; Geisler et al., ([2011](#_ENREF_33)) #203, ([2012](#_ENREF_31)) #203; derived from Kellogg, ([1936](#_ENREF_42)); Yamada, ([1953](#_ENREF_75)); Kasuya, ([1973](#_ENREF_40)) ; Tanaka and Fordyce, (2014, 2015) #164).

(170) Apex of anterior process of periotic in dorsal view: pointed (0); dorsal edge of anterior process showing highly rounded or oblique edge due to its reduction with or without pointed apex (1); thickened by prominent dorsal tubercle giving apex rectangular section in plane of body of periotic (2). (Murakami et al. ([2012a](#_ENREF_57), [2012b](#_ENREF_58)) #165; modified from Fordyce, ([1994](#_ENREF_28)) #53; Lambert, ([2005](#_ENREF_45)) #40; Tanaka and Fordyce, (2014, 2015) #165).

(171) Lateral groove or depression affecting profile of periotic as viewed dorsally: no obvious vertical groove dorsal to hiatus epitympanicus (0); groove present with overall profile of periotic becoming slightly to markedly sigmoidal in dorsal view (1). (Fordyce, ([1994](#_ENREF_28)) #35; Murakami et al. ([2012a](#_ENREF_57), [2012b](#_ENREF_58)) #166; Tanaka and Fordyce, (2014, 2015) #166).

(172) Profile of anterior process of periotic ventrally deflected in lateral view: no, has crudely rectangular profile (0); smoothly deflected (1); abruptly deflected (2). (Fordyce ([1994](#_ENREF_28)) #25; (Tanaka and Fordyce, (2014, 2015) #288)

(173) Anteroposterior ridge on dorsal side: undeveloped (0); developed on anterior process and body of periotic, associated with development of depression adjacent to groove for tensor tympani (1). (Fordyce, ([1994](#_ENREF_28)) #55; Murakami et al. ([2012a](#_ENREF_57), [2012b](#_ENREF_58)) #167; Tanaka and Fordyce, (2014, 2015) #167).

(174) Articulation of anterior process of periotic to outer lip of tympanic bulla: contact of ventral surface of anterior process of periotic with outer lip of tympanic bulla (0); contact with thickened rim of outer lip of tympanic bulla and additionally with accessory ossicle (1); contact only with accessory ossicle (2). (Luo and Marsh, ([1996](#_ENREF_48)) #7; Lambert, ([2005](#_ENREF_45)) #46; Murakami et al. ([2012a](#_ENREF_57), [2012b](#_ENREF_58)) #168; Tanaka and Fordyce, (2014, 2015) #168).

(175) Parabullary sulcus: absent (0); strongly curved, C-shape (1); weakly curved (2); strongly curved, V-shape (3). (modified from Fordyce, ([1994](#_ENREF_28)) #56 Anteroexternal sulcus; Tanaka and Fordyce, (2014, 2015) #169).

(176) Parabullary ridge of periotic: absent (0); present (1); present with a fossa between anterior process and parabullary ridge (2). (modified from Murakami et al. ([2012a](#_ENREF_57), [2012b](#_ENREF_58)) #171; Bianucci, ([2005](#_ENREF_9)) #15; Tanaka and Fordyce, (2014, 2015) #170).

(177) Articulation of anterior process with squamosal: extensive, most of lateral side contacting squamosal (0); large centrally-oriented ovoid region contacting squamosal, free around edges (1); small area of contact with squamosal (2); contact absent, articulation via ligaments (3). (Geisler and Sanders, ([2003](#_ENREF_34)) #207; Geisler et al., ([2011](#_ENREF_33)) #207, ([2012](#_ENREF_31)) #207; Murakami et al. ([2012a](#_ENREF_57), [2012b](#_ENREF_58)) #172; modified from Heyning, ([1997](#_ENREF_37)) #32; Messenger and McGuire, ([1998](#_ENREF_54)) #1490; derived from Heyning, ([1989](#_ENREF_36)) ; Tanaka and Fordyce, (2014, 2015) #171).

(178) Anterior bullar facet: present (0); absent (1). (Muizon, ([1984](#_ENREF_14), [1988](#_ENREF_17), [1991](#_ENREF_18)); Messenger and McGuire, ([1998](#_ENREF_54)) #1496; Lambert, ([2005](#_ENREF_45)) #42; Murakami et al. ([2012a](#_ENREF_57), [2012b](#_ENREF_58)) #173; modified from Fordyce, ([1994](#_ENREF_28)) #4; derived from Kellogg, ([1936](#_ENREF_42)) ; Tanaka and Fordyce, (2014, 2015) #172).

(179) Anterior incisure: deep, pocket-like fossa with anterior groove (0); anterior groove only (1). (Geisler and Luo, ([1996](#_ENREF_32)) #7; Luo and Marsh, ([1996](#_ENREF_48)) #15; Geisler and Sanders, ([2003](#_ENREF_34)) #217; Geisler et al., ([2011](#_ENREF_33)) #217, ([2012](#_ENREF_31)) #217; Murakami et al. ([2012a](#_ENREF_57), [2012b](#_ENREF_58)) #174; Tanaka and Fordyce, (2014, 2015) #173).

(180) Fenestra rotunda: oval to subrounded (0); shaped like teardrop with fissure directed toward aperture for cochlear aqueduct (1). (Fordyce, ([1994](#_ENREF_28)) #22; Geisler and Sanders, ([2003](#_ENREF_34)) #222; Lambert, ([2005](#_ENREF_45)) #49; Geisler et al., ([2011](#_ENREF_33)) #222, ([2012](#_ENREF_31)) #222; Murakami et al. ([2012a](#_ENREF_57), [2012b](#_ENREF_58)) #175; Tanaka and Fordyce, (2014, 2015) #174).

(181) Dorsal surface of periotic in lateral view: convex dorsally (0); pyramidal process convex dorsally (1); nearly flat (2). (Murakami et al. ([2012a](#_ENREF_57), [2012b](#_ENREF_58)) #176; modified from Luo and Marsh, ([1996](#_ENREF_48)) #18; Tanaka and Fordyce, (2014, 2015) #175).

(182) Relative position of dorsal depth of stapedial muscle fossa and fenestra rotunda: ventral to, or in line with, dorsal edge of fenestra rotunda (0); well dorsal to fenestra rotunda (1). (Geisler and Sanders, ([2003](#_ENREF_34)) #223; Geisler et al., ([2011](#_ENREF_33)) #223, ([2012](#_ENREF_31)) #223; Murakami et al. ([2012a](#_ENREF_57), [2012b](#_ENREF_58)) #177; Tanaka and Fordyce, (2014, 2015) #176).

(183) Posterodorsal edge of stapedial muscle fossa: absent, rounded lip (0); present (1). (Geisler and Luo, ([1996](#_ENREF_32)) #14; Geisler and Sanders, ([2003](#_ENREF_34)) #217; Geisler et al., ([2011](#_ENREF_33)) #217, ([2012](#_ENREF_31)) #217; Murakami et al. ([2012a](#_ENREF_57), [2012b](#_ENREF_58)) #178; Tanaka and Fordyce, (2014, 2015) #177).

(184) Caudal tympanic process of periotic: low, its ventral and posterior edges drawing smooth curve (0); Elevated, its ventral and posterior edges forming a right angle in medial view (1). (Geisler and Sanders, ([2003](#_ENREF_34)) #225; Geisler et al., ([2011](#_ENREF_33)) #225, ([2012](#_ENREF_31)) #225; Murakami et al. ([2012a](#_ENREF_57), [2012b](#_ENREF_58)) #179; Tanaka and Fordyce, (2014, 2015) #178).

(185) Position of aperture for cochlear aqueduct: dorsomedial (0); medial (1). (Lambert, ([2005](#_ENREF_45)) #51; Murakami et al. ([2012a](#_ENREF_57), [2012b](#_ENREF_58)) #180; Tanaka and Fordyce, (2014, 2015) #179).

(186) Aperture for cochlear aqueduct: smaller than aperture for vestibular aqueduct (0); approximately same size as aperture for vestibular aqueduct (1); much larger than aperture for vestibular aqueduct, with narrow posterior edge (2). (Geisler and Sanders, ([2003](#_ENREF_34)) #227; Geisler et al., ([2011](#_ENREF_33)) #227, ([2012](#_ENREF_31)) #227; Murakami et al. ([2012a](#_ENREF_57), [2012b](#_ENREF_58)) #181; modified from Muizon, ([1987](#_ENREF_16)); Fordyce, ([1994](#_ENREF_28)); Lambert, ([2005](#_ENREF_45)) #52; Tanaka and Fordyce, (2014, 2015) #180).

(187) Excavation of tegmen tympani at base of anterior process: absent (0); present, with fossa on dorsolateral side of tegmen tympani (1). (Geisler and Sanders, ([2003](#_ENREF_34)) #231; Geisler et al., ([2011](#_ENREF_33)) #231, ([2012](#_ENREF_31)) #231; Murakami et al. ([2012a](#_ENREF_57), [2012b](#_ENREF_58)) #182; Tanaka and Fordyce, (2014, 2015) #181).

(188) Fundus of internal acoustic meatus: funnel-like, smaller at blind end and wider near rim (0); tubular (1). (Luo and Marsh, ([1996](#_ENREF_48)) #31; Geisler and Sanders, ([2003](#_ENREF_34)) #234; Geisler et al., ([2011](#_ENREF_33)) #234, ([2012](#_ENREF_31)) #234; Murakami et al. ([2012a](#_ENREF_57), [2012b](#_ENREF_58)) #183; Tanaka and Fordyce, (2014, 2015) #182).

(189) Internal acoustic meatus: pyriform (0); circular (1). (Muizon, ([1984](#_ENREF_14)); Messenger and McGuire, ([1998](#_ENREF_54)) #1498; Bianucci, ([2005](#_ENREF_9)) #21; Murakami et al. ([2012a](#_ENREF_57), [2012b](#_ENREF_58)) #184; Tanaka and Fordyce, (2014, 2015) #183).

(190) Lateral wall of internal acoustic meatus: high, with wedge-shaped area of elevated bone occurring between dorsal edge of tegmen tympani and internal acoustic meatus, the latter extending ventrally and increasing its depth (0); low, not protruding noticeably from fossa and surrounding bone (1). (Murakami et al. ([2012a](#_ENREF_57), [2012b](#_ENREF_58)) #185; modified from Geisler and Sanders, ([2003](#_ENREF_34)) #235; Geisler et al., ([2011](#_ENREF_33)) #235, ([2012](#_ENREF_31)) #235; Tanaka and Fordyce, (2014, 2015) #184).

(191) Cochlear aqueduct on periotic with a thin edge: no (0); yes (1). (Fordyce ([1994](#_ENREF_28)) #28; Tanaka and Fordyce, (2014, 2015) #289)

(192) Thickness dorsoventral of pars cochlearis on periotic; thick (0) thin (1) (Gutstein et al ([2014](#_ENREF_35)); Tanaka and Fordyce, (2014, 2015) #290)

(193) Profile of cochlear on periotic in dorsoventral; rounded (0), sub-rectangular (1), squared (2). (modified from Fordyce ([1994](#_ENREF_28)) #61 and Bianucci et al ([2013](#_ENREF_10)) #2; Tanaka and Fordyce, (2014, 2015) #291)

(194) Aperture for vestibular aqueduct, in dorsal view: at transverse level of spiral cribriform tract (0); more lateral than spiral cribriform tract (1) (Lambert, ([2005](#_ENREF_45)) #53; Murakami et al. ([2012a](#_ENREF_57), [2012b](#_ENREF_58)) #186; Tanaka and Fordyce, (2014, 2015) #185).

(195) Articular rim: absent (0); present but small, forming ridge anterolateral to articulation surface of posterior process of periotic and separated from it by sulcus (1); present, sigmoidal and laterally elongated with hook-like process (2). (Geisler and Sanders, ([2003](#_ENREF_34)) #239; Geisler et al. ([2012](#_ENREF_31); [2011](#_ENREF_33)) #239; modified from Murakami et al. ([2012a](#_ENREF_57), [2012b](#_ENREF_58)) #187; modified from Muizon ([1987](#_ENREF_16)); Messenger ([1994](#_ENREF_53)); Messenger and McGuire ([1998](#_ENREF_54)) #1494; Fordyce, ([1994](#_ENREF_28)) #33; Lambert, ([2005](#_ENREF_45)) #55; Tanaka and Fordyce, (2014, 2015) #186).

(196) Bony connection between posterior process of periotic and squamosal/occipital bones: present (0); absent (ligamentous). (1). (Muizon, ([1984](#_ENREF_14)); Arnold and Heinsohn,([1996](#_ENREF_3)) #34; Messenger and McGuire, ([1998](#_ENREF_54)) #1491; Murakami et al. ([2012a](#_ENREF_57), [2012b](#_ENREF_58)) #188; derived from Fraser and Purves, ([1960](#_ENREF_30)); Kasuya, ([1973](#_ENREF_40)); Heyning, ([1989](#_ENREF_36)) ; Tanaka and Fordyce, (2014, 2015) #187).

(197) Posterior process of periotic in lateral view: ventrally bent (0); in same plane as body of periotic (1). (Bianucci, ([2005](#_ENREF_9)) #19; Murakami et al. ([2012a](#_ENREF_57), [2012b](#_ENREF_58)) #189; modified from Arnold and Heinsohn,([1996](#_ENREF_3)) #28; Lambert, ([2005](#_ENREF_45)) #54; Tanaka and Fordyce, (2014, 2015) #188).

(198) Angle between posterior process of periotic and long axis of pars cochlearis from dorsal or ventral views: >135° (0); ≤135° (1). (Murakami et al. ([2012a](#_ENREF_57), [2012b](#_ENREF_58)) #190; modified from Geisler and Sanders, ([2003](#_ENREF_34)) #246; Lambert, ([2005](#_ENREF_45)) #54; Geisler et al., ([2011](#_ENREF_33)) #246, ([2012](#_ENREF_31)) #246; derived from Kasuya, ([1973](#_ENREF_40)); Barnes, ([1990](#_ENREF_8)); Luo and Marsh, ([1996](#_ENREF_48)) ; Tanaka and Fordyce, (2014, 2015) #189).

(199) Facet for bulla on posterior process of periotic, parallel-sided; no (0); yes (1). (modified from Fordyce, ([1994](#_ENREF_28)) #63; Tanaka and Fordyce, (2014, 2015) #190).

(200) Ventral surface of posterior process of periotic, along a straight path perpendicular to its long axis: flat (0); concave (1); convex (2). (Murakami et al. ([2012a](#_ENREF_57), [2012b](#_ENREF_58)) #191; modified from Geisler and Sanders, ([2003](#_ENREF_34)) #242; Geisler et al., ([2011](#_ENREF_33)) #242, ([2012](#_ENREF_31)) #242; Tanaka and Fordyce, (2014, 2015) #191).

(201) Posterior bullar facet of periotic: with many long deep grooves and low ridges (0); with some shallow grooves and/or low ridges (1); without grooves or ridges (2). (Bianucci, ([2005](#_ENREF_9)) #20; Murakami et al. ([2012a](#_ENREF_57), [2012b](#_ENREF_58)) #192; Tanaka and Fordyce, (2014, 2015) #192).

(202) Length of posterior process of periotic as percent length of pars cochlearis: long, ≥85% (0); short, ≤84% (1). (Murakami et al. ([2012a](#_ENREF_57), [2012b](#_ENREF_58)) #193; modified from Barnes, ([1990](#_ENREF_8)); Luo and Marsh, ([1996](#_ENREF_48)) #24; Geisler and Sanders, ([2003](#_ENREF_34)) #245; Geisler et al., ([2011](#_ENREF_33)) #245, ([2012](#_ENREF_31)) #245; Tanaka and Fordyce, (2014, 2015) #193).

(203) Mastoid exposure of posterior process of periotic on outside of skull: exposed externally (0); not exposed, enclosed by exoccipital and squamosal (1). (Geisler and Luo, ([1996](#_ENREF_32)) #28; Luo and Marsh, ([1996](#_ENREF_48)) #28; Geisler and Sanders, ([2003](#_ENREF_34)) #249; Geisler et al., ([2011](#_ENREF_33)) #249, ([2012](#_ENREF_31)) #249; Murakami et al. ([2012a](#_ENREF_57), [2012b](#_ENREF_58)) #194; Tanaka and Fordyce, (2014, 2015) #194).

**Tympanic Bulla**

(204) Anterior spine of tympanic bulla: absent (0); present but short (1); present and long (2). (Muizon, ([1987](#_ENREF_16)); Fordyce, ([1994](#_ENREF_28)) #45; Geisler and Sanders, ([2003](#_ENREF_34)) #250; Lambert, ([2005](#_ENREF_45)) #62; Geisler et al., ([2011](#_ENREF_33)) #250, ([2012](#_ENREF_31)) #250;Murakami et al. ([2012a](#_ENREF_57), [2012b](#_ENREF_58)) #195; modified from Messenger and McGuire, ([1998](#_ENREF_54)) #1484; derived from Kasuya, ([1973](#_ENREF_40)) ; Tanaka and Fordyce, (2014, 2015) #195).

(205) Anterolateral convexity of tympanic bulla with anterolateral notch: absent (0); present (1). (Muizon, ([1987](#_ENREF_16)); Fordyce, ([1994](#_ENREF_28)) #46; Lambert, ([2005](#_ENREF_45)) #63; Murakami et al. ([2012a](#_ENREF_57), [2012b](#_ENREF_58))#196; Tanaka and Fordyce, (2014, 2015) #196).

(206) Articulation of posterior process of tympanic bulla with squamosal: process contacting post-tympanic process of squamosal and posterior process of periotic (0); process contacting periotic only (1). (Muizon, ([1984](#_ENREF_14)); Fordyce, ([1994](#_ENREF_28)) #29; Arnold and Heinsohn,([1996](#_ENREF_3)) #34; Messenger and McGuire, ([1998](#_ENREF_54)) #1481; Lambert, ([2005](#_ENREF_45)) #56; Murakami et al. ([2012a](#_ENREF_57), [2012b](#_ENREF_58)) #197; derived Kasuya, ([1973](#_ENREF_40)) ; Tanaka and Fordyce, (2014, 2015) #197).

(207) Width of tympanic bulla as percentage of its length along its long axis: wide, ≥65% (0); narrow and long, ≤64% (1). (Geisler and Sanders, ([2003](#_ENREF_34)) #251; Bianucci, ([2005](#_ENREF_9)) #23; Geisler et al., ([2011](#_ENREF_33)) #251, ([2012](#_ENREF_31)) #251; Murakami et al. ([2012a](#_ENREF_57), [2012b](#_ENREF_58)) #198; derived from Kasuya, ([1973](#_ENREF_40)) ; Tanaka and Fordyce, (2014, 2015) #198).

(208) Accessory ossicle or homologous region on lip of bulla: not fused to anterior process of periotic (0); fused to anterior process of periotic (1). (Barnes, ([1990](#_ENREF_8)); Fordyce, ([1994](#_ENREF_28)); Luo and Marsh, ([1996](#_ENREF_48)); Geisler and Sanders, ([2003](#_ENREF_34)) #255; Geisler et al., ([2011](#_ENREF_33)) #255, ([2012](#_ENREF_31)) #255; Murakami et al. ([2012a](#_ENREF_57), [2012b](#_ENREF_58)) #199; Tanaka and Fordyce, (2014, 2015) #199).

(209) Lateral furrow of tympanic bulla: shallow groove (0); absent (1); deep, well-defined groove (2). (Murakami et al. ([2012a](#_ENREF_57), [2012b](#_ENREF_58)) #200; modified from Muizon, ([1984](#_ENREF_14), [1988](#_ENREF_17)); Arnold and Heinsohn,([1996](#_ENREF_3)) #31; Messenger and McGuire, ([1998](#_ENREF_54)) #1485; Fajardo-Mellor et al., (2006) #17; Lambert, ([2008](#_ENREF_46)) #17; derived from Kasuya, ([1973](#_ENREF_40)) ; Tanaka and Fordyce, (2014, 2015) #200).

(210) Sigmoid process: directed laterally to posterolaterally (0); directed anteriorly to anterolaterally (1). (Murakami et al. ([2012a](#_ENREF_57), [2012b](#_ENREF_58)) #201; modified from Messenger and McGuire, ([1998](#_ENREF_54)) #1486; Lambert, ([2005](#_ENREF_45)) #67, Kasuya, ([1973](#_ENREF_40)); Tanaka and Fordyce, (2014, 2015) #201).

(211) Dorsomedial edge of sigmoid process: expanded anteriorly to appose lateral tuberosity of periotic (0); not articulating with squamosal or periotic (1) (Murakami et al. ([2012a](#_ENREF_57), [2012b](#_ENREF_58)) #202; modified from Geisler and Sanders, ([2003](#_ENREF_34)) #260; Geisler et al., ([2011](#_ENREF_33)) #260, ([2012](#_ENREF_31)) #260; modified from Luo and Marsh, ([1996](#_ENREF_48)) #10; Tanaka and Fordyce, (2014, 2015) #202).

(212) Ventral margin of tympanic bulla in lateral view: convex (0); concave (1). (Lambert, ([2005](#_ENREF_45)) #66; Murakami et al. ([2012a](#_ENREF_57), [2012b](#_ENREF_58)) #203; Tanaka and Fordyce, (2014, 2015) #203).

(213) Elliptical foramen of tympanic bulla: present (0); absent or close (1). (Geisler and Sanders, ([2003](#_ENREF_34)) #261; Geisler et al., ([2011](#_ENREF_33)) #261, ([2012](#_ENREF_31)) #261; Murakami et al. ([2012a](#_ENREF_57), [2012b](#_ENREF_58)) #204; derived from Kasuya, ([1973](#_ENREF_40)) ; Tanaka and Fordyce, (2014, 2015) #204).

(214) Size of posterior process of tympanic bulla: equal to or greater than total length of tympanic bulla (0); much smaller than total length of tympanic bulla (1). (Muizon, ([1984](#_ENREF_14), [1991](#_ENREF_18)); Heyning, ([1989](#_ENREF_36)) #23, 29, ([1997](#_ENREF_37)) #55, 61; Messenger and McGuire, ([1998](#_ENREF_54)) #1482; Murakami et al. ([2012a](#_ENREF_57), [2012b](#_ENREF_58)) #205; modified from Lambert, ([2005](#_ENREF_45)) #57; derived from Yamada, ([1953](#_ENREF_75)); Kasuya, ([1973](#_ENREF_40)) ; Tanaka and Fordyce, (2014, 2015) #205).

(215) Surface of posterior process of tympanic bulla: spiny or irregular edges (0); cauliflower-like bony growth (1); rounded and pachyostotic (2). (Muizon, ([1991](#_ENREF_18)); Messenger and McGuire, ([1998](#_ENREF_54)) #1483; Murakami et al. ([2012a](#_ENREF_57), [2012b](#_ENREF_58)) #206; derived from Kasuya, ([1973](#_ENREF_40)) ; Tanaka and Fordyce, (2014, 2015) #206).

(216) Median furrow: short extension on ventral face anterior to interprominental notch (0); anterolateral curvature of median groove to connect to long lateral furrow on outer lip (1); median groove reaching an anterior level beyond lateral furrow, and often slightly curved laterally (2); long and deep rectilinear median groove reaching at least to base of anterior tip of tympanic bulla (3). (Lambert, ([2005](#_ENREF_45)) #64; Murakami et al. ([2012a](#_ENREF_57), [2012b](#_ENREF_58)) #207; Tanaka and Fordyce, (2014, 2015) #207).

(217) Median furrow on posterior side of bulla: divided by a transverse ridge originating from involucrum (0); transverse ridge absent (1). (Geisler and Sanders, ([2003](#_ENREF_34)) #267; Geisler et al., ([2011](#_ENREF_33)) #267, ([2012](#_ENREF_31)) #267; Murakami et al. ([2012a](#_ENREF_57), [2012b](#_ENREF_58)) #208; Tanaka and Fordyce, (2014, 2015) #208).

(218) Posterior edge of medial prominence of involucrum: approximately in line with posterior edge of lateral prominence (0); distinctly anterior to posterior edge of lateral prominence (1). (Muizon, ([1987](#_ENREF_16)); Geisler and Sanders, ([2003](#_ENREF_34)) #269; Geisler et al., ([2011](#_ENREF_33)) #269, ([2012](#_ENREF_31)) #269; Murakami et al. ([2012a](#_ENREF_57), [2012b](#_ENREF_58)) #209; derived from Kasuya, ([1973](#_ENREF_40)) ; Tanaka and Fordyce, (2014, 2015) #209).

(219) Dorsal margin of involucrum of tympanic bulla: not excavated (0); excavated just anterior to posterior process (1); excavated at mid-part of involucrum (2). (Muizon, ([1988](#_ENREF_17)); Messenger and McGuire, ([1998](#_ENREF_54)) #1487; Murakami et al. ([2012a](#_ENREF_57), [2012b](#_ENREF_58)) #210, 211; modified from Lambert, ([2005](#_ENREF_45)) #60; Geisler and Sanders, ([2003](#_ENREF_34)) #271; Geisler et al., ([2011](#_ENREF_33)) #271, ([2012](#_ENREF_31)) #271; Tanaka and Fordyce, (2014, 2015) #210).

(220) Ridge on inside of bulla: present, as transverse ridge extending laterally from involucrum and partially dividing cavum tympani into anterior and posterior portions (0); absent (1). (Geisler and Sanders, ([2003](#_ENREF_34)) #272; Geisler et al., ([2011](#_ENREF_33)) #272, ([2012](#_ENREF_31)) #272; Murakami et al. ([2012a](#_ENREF_57), [2012b](#_ENREF_58)) #212; Tanaka and Fordyce, (2014, 2015) #211).

(221) Ventromedial keel of tympanic bulla: present along entire length (0); terminating approximately at level of lateral furrow or mid-point of the tympanic bulla (1); poorly defined along entire length (2). (Geisler and Sanders, ([2003](#_ENREF_34)) #273; Geisler et al., ([2011](#_ENREF_33)) #273, ([2012](#_ENREF_31)) #273; Murakami et al. ([2012a](#_ENREF_57), [2012b](#_ENREF_58)) #213; derived from Kasuya, ([1973](#_ENREF_40)) ; Tanaka and Fordyce, (2014, 2015) #212).

(222) Posterior end of ventromedial keel: not protruding and directed medially (0); protruding and directed medially (1). (Geisler and Sanders, ([2003](#_ENREF_34)) #275; Geisler et al., ([2011](#_ENREF_33)) #275, ([2012](#_ENREF_31)) #275; Murakami et al. ([2012a](#_ENREF_57), [2012b](#_ENREF_58)) #214; Tanaka and Fordyce, (2014, 2015) #213).

**Hyals**

(223) Basihyal and thyrohyal connection: unfused (0); fused (1). (Murakami et al. ([2012a](#_ENREF_57), [2012b](#_ENREF_58)) #215; modified from Bianucci, ([2005](#_ENREF_9)) #25; Tanaka and Fordyce, (2014, 2015) #214).

(224) Basihyal and thyrohyal shape: arched (0); angled (1). (Murakami et al. ([2012a](#_ENREF_57), [2012b](#_ENREF_58)) #216; modified from Bianucci, ([2005](#_ENREF_9)) #25; Tanaka and Fordyce, (2014, 2015) #215).

**Vertebrae**

(225) Dorsal transverse process of atlas: developed dorsolaterally (0); fused with ventral transverse process, with height of process greater than width (1); absent or rudimentary obtuse angle (2). (Murakami et al. ([2012a](#_ENREF_57), [2012b](#_ENREF_58)) #217; modified from Muizon, ([1988](#_ENREF_17)); Barnes, ([1990](#_ENREF_8)) ; Tanaka and Fordyce, (2014, 2015) #216).

(226) Roof of neural canal of atlas: arched (0); convex (1); straight (2). (Murakami et al. ([2012a](#_ENREF_57), [2012b](#_ENREF_58)) #218; Tanaka and Fordyce, (2014, 2015) #217).

(227) Postzygapophysis of axis in anterior view: appearing as crest, elongated dorsolaterally (0); appearing as rudimentary crest (1); not appearing (2). (Murakami et al. ([2012a](#_ENREF_57), [2012b](#_ENREF_58)) #219; Tanaka and Fordyce, (2014, 2015) #218).

(228) Cervical vertebrae: unfused (0); atlas and axis fused (1); C1–C3 or C1–C4 fused (2); C1–C6 or C1–C7 fused (3); C2–C7 fused (4). (Murakami et al. ([2012a](#_ENREF_57), [2012b](#_ENREF_58)) #220; modified from Arnold and Heinsohn,([1996](#_ENREF_3)) #9; Messenger and McGuire, ([1998](#_ENREF_54)) #1501; Geisler and Sanders, ([2003](#_ENREF_34)) #278, 279; Fajardo-Mellor et al., (2006) #18; Lambert, ([2008](#_ENREF_46)) #18; Geisler et al., ([2011](#_ENREF_33)) #278, 279, ([2012](#_ENREF_31)) #278, 279; derived from Allen, ([1923](#_ENREF_2)); Miller, ([1923](#_ENREF_55)); Fraser and Noble, ([1971](#_ENREF_60)); De Smet, ([1977](#_ENREF_20)); Rommel, ([1990](#_ENREF_65)) ; Tanaka and Fordyce, (2014, 2015) #219).

(229) Length of cervicals (C1–C7) as percent of height of vertebral body plus neural canal of atlas: long, >150% (0); short, <150% (1). (Murakami et al. ([2012a](#_ENREF_57), [2012b](#_ENREF_58)) #221; Tanaka and Fordyce, (2014, 2015) #220).

(230) Capitular articulation facets of posterior vertebrae: facets gradually shift downward on sequential vertebrae to fuse with tubercular facets (0); facets abruptly shift from a position on neural arch to a pedestal, originating from centrum on subsequent vertebra (1). (Geisler and Sanders, ([2003](#_ENREF_34)) #282; Geisler et al., ([2011](#_ENREF_33)) #282, ([2012](#_ENREF_31)) #282; Murakami et al. ([2012a](#_ENREF_57), [2012b](#_ENREF_58)) #223; derived from Flower, ([1868](#_ENREF_24)); Miller, ([1923](#_ENREF_55)) ; Tanaka and Fordyce, (2014, 2015) #222).

(231) Transverse processes of lumbar vertebrae: extend parallel to anterior and posterior borders (0); triangular (1). (Muizon, ([1984](#_ENREF_14); [1985](#_ENREF_15); [1988](#_ENREF_17)); Messenger and McGuire, ([1998](#_ENREF_54)) #1502; Geisler and Sanders, ([2003](#_ENREF_34)) #285; Geisler et al., ([2011](#_ENREF_33)) #285, ([2012](#_ENREF_31)) #285; Murakami et al. ([2012a](#_ENREF_57), [2012b](#_ENREF_58)) #224; Tanaka and Fordyce, (2014, 2015) #223).

(232) Transverse processes of lumbar vertebrae: oriented ventrolaterally (0); oriented laterally and horizontally (1). (Geisler and Sanders, ([2003](#_ENREF_34)) #284; Geisler et al., ([2011](#_ENREF_33)) #284, ([2012](#_ENREF_31)) #284; Murakami et al. ([2012a](#_ENREF_57), [2012b](#_ENREF_58)) #225; derived from Sanders and Barnes, ([2002](#_ENREF_66)) ; Tanaka and Fordyce, (2014, 2015) #224).

(233) Ratio of greatest breadth of transverse process to width of centrum at anterior face in lumbar vertebrae: some or all lumbar vertebrae >2.5 (0); no lumbar vertebrae >2.5 (1). (Murakami et al. ([2012a](#_ENREF_57), [2012b](#_ENREF_58)) #226; Tanaka and Fordyce, (2014, 2015) #225).

**Sternum and Sternal Ribs**

(234) Sternum: consists of four or five parts (0); consists of two or three parts (1); consists of single bone (2). (Murakami et al. ([2012a](#_ENREF_57), [2012b](#_ENREF_58)) #229; modified from Geisler and Sanders, ([2003](#_ENREF_34)) #290; Geisler et al., ([2011](#_ENREF_33)) #290; derived from Yablokov, ([1964](#_ENREF_74)); Van Valen, ([1968](#_ENREF_72)) ; Tanaka and Fordyce, (2014, 2015) #228).

(235) Ventrolateral processes on manubrium of sternum: absent (0); present but small, occur ventral to articulation surface of first costal cartilage or rib (1). (Muizon, ([1988](#_ENREF_17)); Messenger and McGuire, ([1998](#_ENREF_54)) #1503; Geisler and Sanders, ([2003](#_ENREF_34)) #289; Geisler et al., ([2011](#_ENREF_33)) #289, ([2012](#_ENREF_31)) #289; Murakami et al. ([2012a](#_ENREF_57), [2012b](#_ENREF_58)) #230; derived from Klima et al., ([1980](#_ENREF_43)) ; Tanaka and Fordyce, (2014, 2015) #229).

(236) Sternal ribs: unossified or ossification of fewer than five pairs (0); ossification of five pairs or more (1). (Murakami et al. ([2012a](#_ENREF_57), [2012b](#_ENREF_58)) #231; derived from Flower, ([1867](#_ENREF_23)) ; Tanaka and Fordyce, (2014, 2015) #230).

**Scapula**

(237) Anterodorsal part of scapula: rounded (0); rounded and anterior edge pointed (1); almost rectilinear (2). (Murakami et al. ([2012a](#_ENREF_57), [2012b](#_ENREF_58)) #232; Tanaka and Fordyce, (2014, 2015) #232).

(238) Ventral projection on anterior border of scapula: absent (0); present (1). (Fajardo-Mellor et al., (2006) #26; Murakami et al. ([2012a](#_ENREF_57), [2012b](#_ENREF_58)) #233; derived from Noble and Fraser, ([1971](#_ENREF_60)) ; Tanaka and Fordyce, (2014, 2015) #233).

(239) Anterior slope on scapula between anterior angle and midpoint of glenoid fossa with anterior and posterior margin of glenoid fossa on a plane: shallow, <35° (0); steeper, >35° (1). (modified from Murakami et al. ([2012a](#_ENREF_57), [2012b](#_ENREF_58)) #234; modified from Bianucci, ([2005](#_ENREF_9)) #31; Tanaka and Fordyce, (2014, 2015) #234).

(240) Posterior slope on scapula, between scapula and midpoint of glenoid fossa with anterior and posterior margin of glenoid fossa on a plane: shallow, <25° (0); steeper, >25° (1). (modified from Murakami et al. ([2012a](#_ENREF_57), [2012b](#_ENREF_58)) #235; modified from Bianucci, ([2005](#_ENREF_9)) #32; Tanaka and Fordyce, (2014, 2015) #235).

(241) Crest between infraspinous fossa and teres fossa: weakly developed (0); strongly developed (1). (Murakami et al. ([2012a](#_ENREF_57), [2012b](#_ENREF_58)) #236; Tanaka and Fordyce, (2014, 2015) #236).

(242) Coracoid process of scapula: not expanded distally (0); expanded distally (1); notably reduced or absent (2). (Murakami et al. ([2012a](#_ENREF_57), [2012b](#_ENREF_58)) #237; modified from Muizon, ([1987](#_ENREF_16), [1994](#_ENREF_19)); Messenger and McGuire, ([1998](#_ENREF_54)) #1504; Geisler and Sanders, ([2003](#_ENREF_34)) #292; Lambert, ([2005](#_ENREF_45)) #73; Bianucci, ([2005](#_ENREF_9)) #33; Geisler et al., ([2011](#_ENREF_33)) #292; derived from True, ([1904](#_ENREF_71)) ; Tanaka and Fordyce, (2014, 2015) #237).

(243) Coracoid process of scapula, with glenoid fossa: directed horizontally (0); directed nearly anterodorsally (1); directed anteroventrally (2). (modified from Murakami et al. ([2012a](#_ENREF_57), [2012b](#_ENREF_58)) #238; modified Barnes, ([1990](#_ENREF_8)) ; Tanaka and Fordyce, (2014, 2015) #238).

(244) Acromion of scapula: narrow and not expanded distally (0); expanded distally (1). (Murakami et al. ([2012a](#_ENREF_57), [2012b](#_ENREF_58)) #239; modified from Bianucci, ([2005](#_ENREF_9)) #34; Tanaka and Fordyce, (2014, 2015) #239).

(245) Acromion of scapula, when glenoid fossa direct ventrally: directed horizontally (0); directed anterodorsally (1); directed anteroventrally (2). (Murakami et al. ([2012a](#_ENREF_57), [2012b](#_ENREF_58)) #240; modified from Barnes, ([1990](#_ENREF_8)) ; Tanaka and Fordyce, (2014, 2015) #240).

(246) Supraspinous fossa of scapula: present (0); absent or nearly absent (1). (Muizon, ([1987](#_ENREF_16), [1994](#_ENREF_19)); Geisler and Sanders, ([2003](#_ENREF_34)) #293; Lambert, ([2005](#_ENREF_45)) #72; Geisler et al., ([2012](#_ENREF_31); [2011](#_ENREF_33)) #293; Murakami et al. ([2012a](#_ENREF_57), [2012b](#_ENREF_58)) #241; Tanaka and Fordyce, (2014, 2015) #241).

(247) Acromion process of scapula lies on anterior edge, with loss of supraspinous fossa: no (0); yes (1). (Muizon ([1987](#_ENREF_16)); Fordyce ([1994](#_ENREF_28)) #48; Tanaka and Fordyce, (2014, 2015) #292)

(248) Coracoid process of scapula: present (0); absent (1). (Muizon 1987; Fordyce 1994 #49; Tanaka and Fordyce, (2014, 2015) #293)

**Forelimb (except scapula)**

(249) Ratio of length of humerus to length of radius: long, >1.1 (0); short, <0.8 (1). (Murakami et al. ([2012a](#_ENREF_57), [2012b](#_ENREF_58)) #242; modified from Sanders and Barnes, ([2002](#_ENREF_66)); Geisler and Sanders, ([2003](#_ENREF_34)) #297; Geisler et al., ([2012](#_ENREF_31); [2011](#_ENREF_33)) #297; Tanaka and Fordyce, (2014, 2015) #242).

(250) Location of apex of deltopectoral tuberosity of humerus: within proximal 65% of humerus (0); within distal 35% of humerus (1). (Murakami et al. ([2012a](#_ENREF_57), [2012b](#_ENREF_58)) #243; modified from Muizon, ([1988](#_ENREF_17)); Messenger and McGuire, ([1998](#_ENREF_54)) #1506; Geisler and Sanders, ([2003](#_ENREF_34)) #295; Bianucci, ([2005](#_ENREF_9)) #35; Geisler et al., ([2011](#_ENREF_33)) #295, ([2012](#_ENREF_31)) #295; Tanaka and Fordyce, (2014, 2015) #243).

(251) Prominent deltoid crest on anterior edge of humerus: present, forms greatest anteroposterior diameter along shaft (0); forming a knob-like tuberosity (1); tuberosity or crest absent (2). (Geisler and Sanders, ([2003](#_ENREF_34)) #294; Geisler et al., ([2011](#_ENREF_33)) #294, ([2012](#_ENREF_31)) #294; Murakami et al. ([2012a](#_ENREF_57), [2012b](#_ENREF_58)) #244; derived from Sanders and Barnes, ([2002](#_ENREF_66)) ; Tanaka and Fordyce, (2014, 2015) #244).

(252) Radial and ulnar facets of humerus in lateral view: facets forming a semicircular articulation surface (0); facets forming an obtuse angle (1). (Barnes, [Barnes (1990](#_ENREF_8)); Geisler and Sanders, ([2003](#_ENREF_34)) #296; Geisler et al., ([2011](#_ENREF_33)) #296, ([2012](#_ENREF_31)) #296; Murakami et al. ([2012a](#_ENREF_57), [2012b](#_ENREF_58)) #245; Tanaka and Fordyce, (2014, 2015) #245).

(253) Olecranon process: present as a distinct process (0); present as a slightly raised proximal posterior edge (1); absent (2). (Messenger and McGuire, ([1998](#_ENREF_54)) #1507; Geisler and Sanders, ([2003](#_ENREF_34)) #296; Geisler et al., ([2011](#_ENREF_33)) #284, ([2012](#_ENREF_31)) #284; Murakami et al. ([2012a](#_ENREF_57), [2012b](#_ENREF_58)) #246; modified from Muizon, ([1984](#_ENREF_14)); Barnes, ([1990](#_ENREF_8)); Arnold and Heinsohn,([1996](#_ENREF_3)) #10; Fajardo-Mellor et al., (2006) #28; derived from Howell, ([1927](#_ENREF_38)); [Bianucci (2005](#_ENREF_9)) #37; Tanaka and Fordyce, (2014, 2015) #246).

**Soft Tissues**

(254) Spermaceti organ: absent (0); present (1). (Fordyce, ([1994](#_ENREF_28)) #17; Messenger and McGuire, ([1998](#_ENREF_54)) #1511; Geisler and Sanders, ([2003](#_ENREF_34)) #97; Geisler et al., ([2011](#_ENREF_33)) #97, ([2012](#_ENREF_31)) #97; Murakami et al. ([2012a](#_ENREF_57), [2012b](#_ENREF_58)) #247; derived from Norris and Harvey, ([1972](#_ENREF_62)); Cranford et al., ([1996](#_ENREF_12)) ; Tanaka and Fordyce, (2014, 2015) #247).

(255) Museau de singe: absent (0); present (1). (Messenger and McGuire, ([1998](#_ENREF_54)) #1512; Murakami et al. ([2012a](#_ENREF_57), [2012b](#_ENREF_58)) #248; derived from Norris, ([1964](#_ENREF_61)); Cranford et al., ([1996](#_ENREF_12)) ; Tanaka and Fordyce, (2014, 2015) #248).

(256) Lateral lips of nasal plug: present (0); absent (1). (Messenger and McGuire, ([1998](#_ENREF_54)) #1523; Murakami et al. ([2012a](#_ENREF_57), [2012b](#_ENREF_58)) #249; Tanaka and Fordyce, (2014, 2015) #249).

(257) Proximal sac: single frontal sac (0); sac complex, with nasofrontal sacs and vestibule (1). (Heyning, ([1989](#_ENREF_36)) #6, 11, 17, ([1997](#_ENREF_37)) #33, 43, 49; Fordyce, ([1994](#_ENREF_28)) #16; Messenger and McGuire, ([1998](#_ENREF_54)) #1531, 1532; Lambert, ([2005](#_ENREF_45)) #18; Murakami et al. ([2012a](#_ENREF_57)); [Murakami et al. (2012b](#_ENREF_58)) #250; Tanaka and Fordyce, (2014, 2015) #250).

(258) Posterior nasal sacs: absent (0); present (1). (Heyning, ([1989](#_ENREF_36)) #41, ([1997](#_ENREF_37)) #73; Arnold and Heinsohn,([1996](#_ENREF_3)) #6; Messenger and McGuire, ([1998](#_ENREF_54)) #1534; Murakami et al. ([2012a](#_ENREF_57), [2012b](#_ENREF_58)) #251; Tanaka and Fordyce, (2014, 2015) #251).

(259) Posterior nasal sacs: single (0); divided (1). (Messenger and McGuire, ([1998](#_ENREF_54)) #1535; Murakami et al. ([2012a](#_ENREF_57)) #252; Tanaka and Fordyce, (2014, 2015) #252).

(260) Anterior section of nasofrontal sac: absent (0); present (1). (Messenger and McGuire, ([1998](#_ENREF_54)) #1536; Murakami et al. ([2012a](#_ENREF_57), [2012b](#_ENREF_58)) #253; Tanaka and Fordyce, (2014, 2015) #253).

(261) Anterior part of nasofrontal sac: smooth (0); trabeculate (1). (Messenger and McGuire, ([1998](#_ENREF_54)) #1537; Fajardo-Mellor et al., (2006) #35; Murakami et al. ([2012a](#_ENREF_57), [2012b](#_ENREF_58)) #254; derived from Heyning, ([1989](#_ENREF_36)) ; Tanaka and Fordyce, (2014, 2015) #254).

(262) Vestibular sac: absent (0); present (1); hypertrophied (2). (Heyning, ([1989](#_ENREF_36)) #28, ([1997](#_ENREF_37)) #60; Fordyce, ([1994](#_ENREF_28)) #31; Arnold and Heinsohn,([1996](#_ENREF_3)) #1, 3; Messenger and McGuire, ([1998](#_ENREF_54)) #1541; Lambert, ([2005](#_ENREF_45)) #17; Fajardo-Mellor et al., (2006) #39; Murakami et al. ([2012a](#_ENREF_57), [2012b](#_ENREF_58)) #255; Tanaka and Fordyce, (2014, 2015) #255).

(263) Floor of vestibular sac (nasal sac): not rigid (0); rigid (1). (Heyning, ([1989](#_ENREF_36)) #38, ([1997](#_ENREF_37)) #70; Arnold and Heinsohn,([1996](#_ENREF_3)) #2; Messenger and McGuire, ([1998](#_ENREF_54)) #1543; Fajardo-Mellor et al., (2006) #38; Murakami et al. ([2012a](#_ENREF_57), [2012b](#_ENREF_58)) #256; Tanaka and Fordyce, (2014, 2015) #256).

(264) Vestibular sac (nasal sac): undivided (0); bilaterally divided (1). (Messenger and McGuire, ([1998](#_ENREF_54)) #1544; Fajardo-Mellor et al., (2006) #36; Murakami et al. ([2012a](#_ENREF_57), [2012b](#_ENREF_58)) #257; derived from Heyning, ([1989](#_ENREF_36)) ; Tanaka and Fordyce, (2014, 2015) #257).

(265) Right and left sides of vestibular sac (nasal sac): same size (0); right side larger than left (1). (Heyning, ([1989](#_ENREF_36)) #30, ([1997](#_ENREF_37)) #62; Messenger and McGuire, ([1998](#_ENREF_54)) #1545; Murakami et al. ([2012a](#_ENREF_57), [2012b](#_ENREF_58)) #258; Tanaka and Fordyce, (2014, 2015) #258).

(266) Intrinsic muscle in vestibular sac (nasal sac): absent (0); present (1). (Messenger and McGuire, ([1998](#_ENREF_54)) #1546; Fajardo-Mellor et al., (2006) #37; Murakami et al. ([2012a](#_ENREF_57), [2012b](#_ENREF_58)) #259; derived from Mead, ([1975](#_ENREF_51)) ; Tanaka and Fordyce, (2014, 2015) #259).

(267) Floor of vestibular sac (nasal sac): smooth (0); wrinkled (1). (Heyning, ([1997](#_ENREF_37)) #70; Arnold and Heinsohn,([1996](#_ENREF_3)) #2; Messenger and McGuire, ([1998](#_ENREF_54)) #1543; Murakami et al. ([2012a](#_ENREF_57), [2012b](#_ENREF_58)) #260; Tanaka and Fordyce, (2014, 2015) #260).

(268) Diagonal membrane: absent (0); present (1). (Messenger and McGuire, ([1998](#_ENREF_54)) #1550; Murakami et al. ([2012a](#_ENREF_57), [2012b](#_ENREF_58)) #261; Heyning, ([1989](#_ENREF_36)) ; Tanaka and Fordyce, (2014, 2015) #261).

(269) Spiracular cavity: slit-like (0); rounded (1). (Messenger and McGuire, ([1998](#_ENREF_54)) #1552; Murakami et al. ([2012a](#_ENREF_57), [2012b](#_ENREF_58)) #262; Tanaka and Fordyce, (2014, 2015) #262).

(270) Pars posteroexternus muscle: absent (0); present (1). (Messenger and McGuire, ([1998](#_ENREF_54)) #1553; Murakami et al. ([2012a](#_ENREF_57), [2012b](#_ENREF_58)) #263; Tanaka and Fordyce, (2014, 2015) #263).

(271) Pars intermedius muscle: absent (0); present (1). (Messenger and McGuire, ([1998](#_ENREF_54)) #1554; Murakami et al. ([2012a](#_ENREF_57), [2012b](#_ENREF_58)) #264; Tanaka and Fordyce, (2014, 2015) #264).

(272) Pars posterointerus muscle: absent (0); present (1). (Messenger and McGuire, ([1998](#_ENREF_54)) #1556; Murakami et al. ([2012a](#_ENREF_57), [2012b](#_ENREF_58)) #265; Tanaka and Fordyce, (2014, 2015) #265).

(273) Pars anterointerus muscle: one insertion (0); two insertions (1). (Messenger and McGuire, ([1998](#_ENREF_54)) #1557; Murakami et al. ([2012a](#_ENREF_57), [2012b](#_ENREF_58)) #266; Tanaka and Fordyce, (2014, 2015) #266).

(274) Blowhole shape: longitudinal slit, may be slightly sigmoidal or angled (0); crescent, with apices pointed anteriorly (1); crescent, with apices pointed posteriorly, may be skewed (2); rectangular (3). (Murakami et al. ([2012a](#_ENREF_57), [2012b](#_ENREF_58)) #267; modified from Messenger and McGuire, ([1998](#_ENREF_54)) #1525; Tanaka and Fordyce, (2014, 2015) #267).

(275) Soft tissues of nasal passages distal to bony external nares: separated for most of their length but confluent just proximal to blowhole (0); confluent (1). (Heyning, ([1989](#_ENREF_36)); Fordyce, ([1994](#_ENREF_28)) #20; Messenger and McGuire, ([1998](#_ENREF_54)) #1529; Geisler and Sanders, ([2003](#_ENREF_34)) #95; Lambert, ([2005](#_ENREF_45)) #16; Geisler et al., ([2011](#_ENREF_33)) #95, ([2012](#_ENREF_31)) #95; Murakami et al. ([2012a](#_ENREF_57), [2012b](#_ENREF_58)) #268; Tanaka and Fordyce, (2014, 2015) #268).

(276) Distal sac: absent (0); present, situated immediately distal to museau de singe (1). (Murakami et al. ([2012a](#_ENREF_57), [2012b](#_ENREF_58)) #269; modified from Heyning, ([1989](#_ENREF_36)) #12, ([1997](#_ENREF_37))#44; Fordyce, ([1994](#_ENREF_28)) #14; Messenger and McGuire, ([1998](#_ENREF_54)) #1533; Geisler and Sanders, ([2003](#_ENREF_34)) #99, ([2012](#_ENREF_31)) #99; Lambert, ([2005](#_ENREF_45)) #19; Geisler et al., ([2011](#_ENREF_33)) #99; Tanaka and Fordyce, (2014, 2015) #269).

(277) Blowhole ligament: absent (0); present (1). (Heyning, ([1989](#_ENREF_36)) #15, ([1997](#_ENREF_37)) #44; Fordyce, ([1994](#_ENREF_28)) #13; Messenger and McGuire, ([1998](#_ENREF_54)) #1527; Geisler and Sanders, ([2003](#_ENREF_34)) #101; Lambert, ([2005](#_ENREF_45)) #20; Geisler et al., ([2011](#_ENREF_33)) #101, ([2012](#_ENREF_31)) #101; Murakami et al. ([2012a](#_ENREF_57), [2012b](#_ENREF_58)) #270; Tanaka and Fordyce, (2014, 2015) #270).

(278) Blowhole ligament: not appressed against skull (0); appressed against skull (1). (Messenger and McGuire, ([1998](#_ENREF_54)) #1528; Murakami et al. ([2012a](#_ENREF_57), [2012b](#_ENREF_58)) #271; Tanaka and Fordyce, (2014, 2015) #271).

(279) Cartilage on blowhole ligament: absent (0); present (1). (Messenger and McGuire, ([1998](#_ENREF_54)) #1529; Murakami et al. ([2012a](#_ENREF_57), [2012b](#_ENREF_58)) #272; Tanaka and Fordyce, (2014, 2015) #272).

(280) Accessory sac: absent (0); present, forms small diverticulum of inferior vestibule and extends anterolaterally around the attachment of blowhole ligament to the premaxilla (1). (Messenger and McGuire, ([1998](#_ENREF_54)) #1549; Geisler and Sanders, ([2003](#_ENREF_34)) #106; Fajardo-Mellor et al., (2006) #40; Geisler et al., ([2011](#_ENREF_33)) #106, ([2012](#_ENREF_31)) #106; Murakami et al. ([2012a](#_ENREF_57), [2012b](#_ENREF_58)) #274; derived from Schenkkan, ([1971](#_ENREF_67)); Mead, ([1975](#_ENREF_51)); Heyning, ([1989](#_ENREF_36)) ; Tanaka and Fordyce, (2014, 2015) #273).

(281) Esophageal forestomach: present (0); absent (1). (Geisler and Sanders, ([2003](#_ENREF_34)) #300; Geisler et al., ([2011](#_ENREF_33)) #300, ([2012](#_ENREF_31)) #300; Murakami et al. ([2012a](#_ENREF_57), [2012b](#_ENREF_58)) #275; derived from Mead, ([1989](#_ENREF_50)); Rice and Wolman, ([1990](#_ENREF_64)) ; Tanaka and Fordyce, (2014, 2015) #274).

(282) External throat grooves: absent (0); one pair converged anteriorly (1); irregular in number and shape (2). (Murakami et al. ([2012a](#_ENREF_57)) #276; modified from Messenger and McGuire, ([1998](#_ENREF_54)) #1512, 1513; Geisler and Sanders, ([2003](#_ENREF_34)) #301; Geisler et al., ([2011](#_ENREF_33)) #301, ([2012](#_ENREF_31)) #301; Tanaka and Fordyce, (2014, 2015) #276).

(283) Dorsal fin: present (0); dorsal hump (1); absent (2). (Murakami et al. ([2012a](#_ENREF_57)) #277; modified from Messenger and McGuire, ([1998](#_ENREF_54)) #1562; Geisler and Sanders, ([2003](#_ENREF_34)) #304; Geisler and Sanders, 2003 #304, ([2012](#_ENREF_31)) #304; derived from Leatherwood and Reeves, ([1983](#_ENREF_47)); Jefferson and Newcomer ([1993](#_ENREF_39)), Reeves et al. ([2002](#_ENREF_63)) ; Tanaka and Fordyce, (2014, 2015) #277).

(284) Shape of flipper: fan shaped (0); rounded at tip (1); sharply pointed at tip (2); entire flipper rounded (3). (Murakami et al. ([2012a](#_ENREF_57)) #278; modified from Fajardo-Mellor et al., (2006) #31; Lambert, ([2008](#_ENREF_46)) #25; derived from Leatherwood and Reeves ([1983](#_ENREF_47)), Brownell et al. ([1987](#_ENREF_11)) ; Tanaka and Fordyce, (2014, 2015) #278).

References

Aguirre-Fernández, G., Barnes, L.G., Aranda-Manteca, F.J., and Fernández-Rivera, J.R. 2009. *Protoglobicephala mexicana*, a new genus and species of Pliocene fossil dolphin (Cetacea; Odontoceti; Delphinidae) from the Gulf of California, Mexico. *Boletin de la Sociedad Geologica Mexicana,* 61(2):245-265.

Allen, G.M. 1923. The black finless porpoise, Meomeris. *Bulletin of the Museum of Comparative Zoology,* 65(7):233-256.

Arnold, P.W., and Heinsohn, G.E. 1996. Phylogenetic status of the Irrawaddy dolphin *Orcaella brevirostris* (Owen in Gray): a cladistic analysis. *Memoirs of the Queensland Museum,* 39(2):141-204.

Barnes, L.G. 1984a. Fossil odontocetes (Mammalia: Cetacea) from the Almejas Formation, Isla Cedros, Mexico. *Paleobios,* 42:1-46.

Barnes, L.G. 1984b. Whales, dolphins and porpoises; origin and evolution of the Cetacea. In Gingerich, P.D., and Badgle, C.E. (eds.), *Mammals. Notes for a short course*, University of Atennessee, Department of Geological Science.

Barnes, L.G. 1985a. Evolution, taxonomy and antitropical distributions of the porpoises (Phocoenidae, Mammalia). *Marine Mammal Science,* 1(2):149-165.

Barnes, L.G. 1985b. Fossil pontoporiid dolphins (Mammalia: Cetacea) from the Pacific coast of North America. *Contributions to Science, Natural History Museum of Los Angeles County,* 363:1-34.

Barnes, L.G. 1990. The fossil record and evolutionary relationships of the genus *Tursiops*, p. 3-26. In Leatherwood, S., and Reeves, R.R. (eds.), *The bottlenose dolphin.* Academic Press Inc, San Diego, New York.

Bianucci, G. 2005. *Arimidelphis sorbinii* a new small killer whale-like dolphin from the Pliocene of Marecchia River (Central eastern Italy) and a phylogenetic analysis of the Orcininae (Cetacea: Odontoceti). *Rivista Italiana di Paleontologia e Stratigrafia,* 111(2):329-344.

Bianucci, G., Lambert, O., Salas-Gismondi, R., Tejada, J., Pujos, F., Urbina, M., and Antoine, P.-O. 2013. A Miocene relative of the Ganges River dolphin (Odontoceti, Platanistidae) from the Amazonian Basin. *Journal of Vertebrate Paleontology,* 33(3):741-745.

Brownell, R.L., Findley, L.T., Vidal, O., Robles, A., and Silvia Manzanilla, N. 1987. External morphology and pigmentation of the vaquita, *Phocoena sinus* (Cetacea: Mammalia). *Marine Mammal Science,* 3(1):22-30.

Cranford, T.W., Amundin, M., and Norris, K.S. 1996. Functional morphology and homology in the odontocete nasal complex: implications for sound generation. *Journal of Morphology,* 228(3):223-285.

Curry, B.E. 1992. Facial anatomy and potential function of facial structures for sound production in the harbor porpoise (*Phocoena phocoena*) and Dall's porpoise (*Phocoenoides dalli*). *Canadian Journal of Zoology,* 70(11):2103-2114.

de Muizon, C. 1984. Les vertébrés fossiles de la Formation Pisco (Pérou). deuxiéme partie: les Odontocétes (Cetacea, Mammalia) du Pliocéne inférieur de Sud-Sacaco. *Travaux de l’Institut Français d’Études Andines,* 27:1-188.

de Muizon, C. 1985. Nouvelles données sur le diphylétisme des Dauphins de rivière (Odontoceti, Cetacea, Mammalia). *Comptes rendus l'Academie des Sciences series 2,,* 301:359-362.

de Muizon, C. 1987. The affinities of *Notocetus vanbenedeni*, an Early Miocene platanistoid (Cetacea, Mammalia) from Patagonia, southern Argentina. *American Museum novitates,* 2904:1-27.

de Muizon, C. 1988. Les relations phylogenetiques des Delphinida (Cetacea, mammalia). *Annales de paléontologie,* 74(4):159-227.

de Muizon, C. 1991. A new Ziphiidae (Cetacea) from the Early Miocene of Washington State (USA) and phylogenetic analysis of the major groups of odontocetes. *Bulletin du Muséum National d'Histoire Naturelle,* 12(3-4):279-326.

de Muizon, C. 1994. Are the squalodonts related to the platanistoids? *Proceedings of the San Diego Society of Natural History,* 29:135-146.

De Smet, W.M.A. 1977. The regions of the cetacean vertebral column volume 3, p. 59-80. In Harrison, R.J. (ed.), *Functional anatomy of marine mammals*. Academic Press, London.

Doran, A.H. 1878. Morphology of the mammalian ossicula auditus. *Transactions of the Linnaean Society, Series 2, Zoology,* 1(18):371-497.

Fajardo‐Mellor, L., Berta, A., Brownell, R.L., Boy, C.C., and Goodall, N.P. 2006. The phylogenetic relationships and biogeography of true porpoises (Mammalia: Phocoenidae) based on morphological data. *Marine Mammal Science,* 22(4):910-932.

Flower, W.H. 1867. Description of the skeleton of *Inia geoffrensis* and the skull of *Pontoporia blainvillii*, with remarks on the systematic position of these animals in the Order Cetacea. *Transactions of the Zoological Society of London,* 6(3):87-116.

Flower, W.H. 1868. On the osteology of the cachalot or sperm-whale (Physeter macrocephalus). *Trans Zool Soc Lond,* 6(6):309-372.

Flower, W.H. 1872. On the recent ziphioid whales, with a description of the skeleton of *Berardius arnouxi*. *The Transactions of the Zoological Society of London,* 8(3):203-234.

Flower, W.H. 1884. On the characters and divisions of the Family Delphinidae. *Proceedings of the Zoological Society of London,* 1883:466-513.

Flower, W.H. 1885. *List of the specimens of Cetacea in the Zoological Department of the British Museum*. British Museum, London.

Fordyce, R.E. 1994. *Waipatia maerewhenua*, new genus and new species (Waipatiidae, new family), an archaic Late Oligocene dolphin (Cetacea: Odontoceti: Platanistoidea) from New Zealand. *Proceedings of the San Diego Society of Natural History,* 29:147-176.

Fordyce, R.E. 2002. *Simocetus rayi* (Odontoceti: Simocetidae, new family): A bizarre new archaic Oligocene dolphin from the eastern North Pacific. *Smithsonian Contributions to Paleobiology,* 93:185-222.

Fraser, F.C., and Purves, P.E. 1960. Hearing in cetaceans: evolution of the accessory air sacs and the structure of the outer and middle ear in recent cetaceans. *Bulletin of the British Museum of Natural History (Zoology)* 7:1-140.

Geisler, J.H., Godfrey, S.J., and Lambert, O. 2012. A new genus and species of late Miocene inioid (Cetacea, Odontoceti) from the Meherrin River, North Carolina, USA. *Journal of Vertebrate Paleontology,* 32(1):198-211.

Geisler, J.H., and Luo, Z. 1996. The petrosal and inner ear of *Herpetocetus* sp.(Mammalia: Cetacea) and their implications for the phylogeny and hearing of archaic mysticetes. *Journal of Paleontology*:1045-1066.

Geisler, J.H., McGowen, M.R., Yang, G., and Gatesy, J. 2011. A supermatrix analysis of genomic, morphological, and paleontological data from crown Cetacea. *BMC Evolutionary Biology,* 11:1-33.

Geisler, J.H., and Sanders, A.E. 2003. Morphological evidence for the phylogeny of Cetacea. *Journal of Mammalian Evolution,* 10(1/2):23-129.

Gutstein, C.S., Figueroa-Bravo, C.P., Pyenson, N.D., Yury-Yañez, R.E., Cozzuol, M.A., and Canals, M. 2014. High frequency echolocation, ear morphology, and the marine–freshwater transition: A comparative study of extant and extinct toothed whales. *Palaeogeography, Palaeoclimatology, Palaeoecology,* 400:62-74.

Heyning, J.E. 1989. Comparative facial anatomy of beaked whales (Ziphiidae) and a systematic revision among the families of extant Odontoceti. *Contributions in Science, Natural History Museum of Los Angeles County.,* 405:1-64.

Heyning, J.E. 1997. Sperm whale phylogeny revisited: analysis of the morphological evidence. *Marine Mammal Science,* 13(4):596-613.

Howell, A.B. 1927. Contribution to the anatomy of the Chinese finless porpoise *Neomeris phocaenoides*. *Proceedings of The United States National Museum,* 70(13):1-43.

Jefferson, T.A., and Newcomer, M.W. 1993. *Lissodelphis borealis*. *Mammalian Species*:1-6.

Kasuya, T. 1973. Systematic consideration of recent toothed whales based on the morphology of tympano-periotic bone. *Scientific Reports of the Whales Research Institute, Tokyo,* 25:1-103.

Kellogg, R. 1923. Description of an apparently new toothed cetacean from South Carolina. *Smiths Misc Coll,* 76:1-7.

Kellogg, R. 1936. A review of the Archaeoceti. *Carnegie Institution of Washington publication,* 482:1-366.

Klima, M., Oelschlaeger, H.A., and Wünsch, D. 1980. Morphology of the pectoral girdle in the Amazon dolphin *Inia geoffrensis* with special reference to the shoulder joint and the movements of the flippers. *Zeitschrift Saugertierkunde,* 45:288-309.

Kuzmin, A.A. 1976. Embryogenesis of the osseous skull of the sperm whale (Physeter macrocephalus Linnaeus, 1758). *Investigations on Cetacea,* 7:187-202.

Lambert, O. 2005. Phylogenetic affinities of the long-snouted dolphin *Eurhinodelphis* (Cetacea, Odontoceti) from the Miocene of Antwerp, Belgium. *Palaeontology,* 48(3):653-679.

Lambert, O. 2008. A new porpoise (Cetacea, Odontoceti, Phocoenidae) from the Pliocene of the North Sea. *Journal of Vertebrate Paleontology,* 28(3):863-872.

Leatherwood, S., Reeves, R.R., and Foster, L. 1983. *Sierra Club handbook of whales and dolphins*. Sierra Club Books, San Francisco.

Luo, Z., and Marsh, K. 1996. Petrosal (periotic) and inner ear of a Pliocene kogiine whale (Kogiinae, Odontoceti): implications on relationships and hearing evolution of toothed whales. *Journal of Vertebrate Paleontology,* 16(2):328-348.

Marsh, H., Lloze, R., Heinsohn, G.E., and Kasuya, T. 1989. Irrawaddy dolphin - *Orcaella brevirostris*, (Gray, 1866), p. 101-118. In Ridgway, S.H., and Harrison, S. (eds.), *Handbook of marine mammals. Volume 4: river dolphins and the larger toothed whales.*

Mead, J. 1989. Shepherd’s beaked whale *Tasmacetus shepherdi* Oliver, 1937. *Handbook of marine mammals. Volume 4: river dolphins and the larger toothed whales.,* 4:309-320.

Mead, J.G. 1975. Anatomy of the external nasal passages and facial complex in the Delphinidae(Mammalia, Cetacea). *Smithsonian Contributions to Zoology,* 207:1-72.

Mead, J.G., and Fordyce, R.E. 2009. The therian skull: a lexicon with emphasis on the odontocetes. *Smithsonian Contributions to Zoology,* 627:1-248.

Messenger, S. 1994. Phylogenetic relationships of platanistoid river dolphins (Odontoceti, Cetacea): assessing the significance of fossil taxa. *Contributions in Marine Mammal Paleontology Honoring Frank Whitmore Jr.Proceedings of the San Diego Society of Natural History*:125-133.

Messenger, S.L., and McGuire, J.A. 1998. Morphology, molecules, and the phylogenetics of cetaceans. *Systematic Biology,* 47(1):90-124.

Miller, G.S. 1923. The telescoping of the cetacean skull. *Smithsonian Miscellaneous Collections,* 76(5):1-70.

Moore, J.C. 1968. Relationships among the living genera of beaked whales with classifications, diagnoses and keys. *Fieldiana: Zoology,* 53(4):509-598.

Murakami, M., Shimada, C., Hikida, Y., and Hirano, H. 2012a. A new basal porpoise, *Pterophocaena nishinoi* (Cetacea, Odontoceti, Delphinoidea), from the upper Miocene of Japan and its phylogenetic relationships. *Journal of Vertebrate Paleontology,* 32(5):1157-1171.

Murakami, M., Shimada, C., Hikida, Y., and Hirano, H. 2012b. Two new extinct basal phocoenids (Cetacea, Odontoceti, Delphinoidea), from the upper Miocene Koetoi Formation of Japan and their phylogenetic significance. *Journal of Vertebrate Paleontology,* 32(5):1172-1185.

Murakami, M., Shimada, C., Hikida, Y., Soeda, Y., and Hirano, H. 2014. *Eodelphis kabatensis*, a new name for the oldest true dolphin *Stenella kabatensis* Horikawa, 1977 (Cetacea, Odontoceti, Delphinidae), from the upper Miocene of Japan, and the phylogeny and paleobiogeography of Delphinoidea. *Journal of Vertebrate Paleontology,* 34(3):491-511.

Noble, B., and Fraser, F. 1971. Description of a skeleton and supplementary notes on the skull of a rare porpoise *Phocoena sinus* Norris & McFarland 1958. *Journal of Natural History,* 5(4):447-464.

Norris, K.S. 1964. Some problems of echolocation in cetaceans, p. 317-336. In Tavolga, W.N. (ed.), *Marine bio-acoustics*. MacMillan, New York.

Norris, K.S., and Harvey, G.W. 1972. A theory for the function of the spermaceti organ of the sperm whale (*Physeter catodon L*). *NASA Special Publication,* 262:397.

Reeves, R., Stewart, B., Clapham, P., and Powell, J. 2002. Marine mammals of the world. Chanticleer Press, Inc, New York.

Rice, D.W., and Wolman, A.A. 1990. The stomach of *Kogia breviceps*. *Journal of Mammalogy*:237-242.

Rommel, S. 1990. Osteology of the bottlenose dolphin, p. 29-49. In Leatherwood, S., and Reeves, R.R. (eds.), *The bottlenose dolphin*. Academic Press Inc, San Diego, New York.

Sanders, A.E., and Barnes, L.G. 2002. Paleontology of the late Oligocene Ashley and Chandler Bridge formations of South Carolina, 2: *Micromysticetus rothauseni*, a primitive cetotheriid mysticete (Mammalia: Cetacea). *Smithsonian Contributions to Paleobiology*(93):271-293.

Schenkkan, E. 1971. The occurrence and position of the “connecting sac” in the nasal tract complex of small odontocetes (Mammalia, Cetacea). *Beaufortia,* 19(246):37-43.

Schulte, H.v.W. 1917. The skull of *Kogia breviceps* Blainv. *Bulletin of the American Museum of Natural History*(37):361-404.

Tanaka, Y., and Fordyce, R.E. 2014. Fossil dolphin *Otekaikea marplesi* (latest Oligocene, New Zealand) expands the morphological and taxonomic diversity of Oligocene cetaceans. *PLoS ONE,* 9(9):e107972.

Tanaka, Y., and Fordyce, R.E. 2015. A new Oligo-Miocene dolphin from New Zealand: *Otekaikea huata* expands diversity of the early Platanistoidea. *Palaeontologia Electronica,* 18(2.23A):1-71.

True, F.W. 1904. The whalebone whales of the western North Atlantic compared with those occurring in European waters with some observations on the species of the North Pacific. *Smithson Contribution to Knowledge,* 33:1-332.

Van Valen, L. 1968. Monophyly or diphyly in the origin of whales. *Evolution*:37-41.

Whitmore, F.C., and Sanders, A.E. 1977. Review of the Oligocene Cetacea. *Systematic Zoology,* 25(4):304-320.

Yablokov, A. 1964. Convergence or parallelism in the evolution of cetaceans. *International Geology Review,* 7(8):1461-1468.

Yamada, M. 1953. Contribution to the anatomy of the organ of hearing of whales. *Scientific Reports of the Whales Research Institute,* 8:1-79.

Zhou, K. 1982. Classification and phylogeny of the superfamily Platanistoidea, with notes on evidence of the monophyly of the Cetacea. *Scientific Reports of the Whales Research Institute Tokyo,* 34:93-108.
