# Supplementary material for: A new tropical Oligocene dolphin from Montañita/Olón, Santa Elena, Ecuador
Source: PLoS One. 2017 Dec 20;12(12):e0188380. doi: 10.1371/journal.pone.0188380 (PMC5737981; doi:10.1371/journal.pone.0188380)
Supplement: S5 File — (DOCX) [file pone.0188380.s007.docx]

Appendix 5.

List of modifications to the original codings and character descriptioons of [Tanaka and Fordyce (2016](#_ENREF_1)). The initial number refers to the character number in Appendix 3. Character numbers are identified by a hatch #.

(177) Articulation of anterior process with squamosal: extensive, most of lateral side contacting squamosal (0); large centrally-oriented ovoid region contacting squamosal, free around edges (1); small area of contact with squamosal (2); contact absent, articulation via ligaments (3).

*Otekaikea huata* ? to 0

(193) Profile of cochlear on periotic in dorsoventral; rounded (0), sub-rectangular (1), squared (2).

ZMT 73 ? to 0

(195) Articular rim: absent (0); present but small, forming ridge anterolateral to articulation surface of posterior process of periotic and separated from it by sulcus (1); present, sigmoidal and laterally elongated with hook-like process (2).

ZMT 73 ? to 0

(208) Accessory ossicle or homologous region on lip of bulla: not fused to anterior process of periotic (0); fused to anterior process of periotic (1).

“to anterior process of periotic” is added for state (0).

References

Tanaka, Y., and R. E. Fordyce. 2016. *Papahu*-like fossil dolphin from Kaikoura, New Zealand, helps to fill the Early Miocene gap in the history of Odontoceti. New Zealand Journal of Geology and Geophysics.
